# Asymmetric Diphosphane Dioxides With A–π–A–π’–D Scaffolds for High‐Purity Deep‐Blue Luminescence

**DOI:** 10.1002/chem.71232

**Published:** 2026-06-10

**Authors:** Eetu Hakkarainen, Zong‐Ying Liu, Jhon Sebastian Oviedo Ortiz, Nicolas Vanthuyne, Jeanne Crassous, Ondřej Mrózek, Fabien B. L. Cougnon, Wen‐Yi Hung, Pi‐Tai Chou, Igor O. Koshevoy, Andrey Belyaev

**Affiliations:** ^1^ Department of Chemistry and Sustainable Technology University of Eastern Finland Joensuu Finland; ^2^ Department of Chemistry National Taiwan University Taipei Taiwan; ^3^ CNRS ISCR‐UMR 6226 Institut Des Sciences Chimiques De Renne Université De Rennes Rennes France; ^4^ CNRS Centrale Med, FSCM, Chiropole Aix Marseille University Marseille France; ^5^ Institute of Inorganic Chemistry of the Czech Academy of Sciences Husinec‐Řež Czech Republic; ^6^ Department of Chemistry Nanoscience Center University of Jyväskylä Jyväskylä Finland; ^7^ Department of Optoelectronics and Materials Technology National Taiwan Ocean University Keelung Taiwan

**Keywords:** chiral resolution, deep‐blue electroluminescence, luminescence, OLED, phosphane oxides

## Abstract

Phosphane oxides represent a cornerstone unit in modern optoelectronic materials due to their strong electron‐withdrawing character, high thermal stability, and ability to tune excited‐state properties. Herein, we report a streamlined design strategy toward donor–π–acceptor–π’–acceptor (*Γ*–type) systems incorporating dual phosphane oxide units as both intermediate and terminal acceptors. A series of diphosphane dioxides (**1**–**3**) was synthesized and characterized, revealing pronounced deep‐blue fluorescence (*λ*_em_ = 385–418 nm in toluene, *λ*_em_ = 415–457 nm in solid) that stems from charge‐transfer and locally excited states with high photoluminescence yields up to 95%. Structure–property relationships demonstrate that subtle variations in π‐conjugation (π = phenyl, biphenyl) and donor strength govern optical bandgap, oscillator strength, excited‐state dipole moments, and intersystem crossing pathways. Notably, **2** and **3** exhibit favorable thermal stability and high triplet energies, enabling their application as emitters in deep‐blue OLEDs. Devices based on **2** exhibit a maximum external quantum efficiency (EQE) of 4.86%, delivering high blue color purity with CIE coordinates of (0.15, 0.07), while maintaining low turn‐on voltages and minimal efficiency roll‐off. Transient electroluminescence studies indicate a significant contribution from triplet–triplet annihilation, enhancing exciton utilization beyond the conventional singlet limit.

## Graphical abstract

Diphosphane dioxide‐based luminophores with donor–π–acceptor– π’–acceptor scaffold enable efficient deep‐blue emission from tunable charge‐transfer states affording high photoluminescence up to 95%. OLEDs reach EQE 4.9%, aided by triplet–triplet annihilation, highlighting a practical route to high‐performance blue emitters.

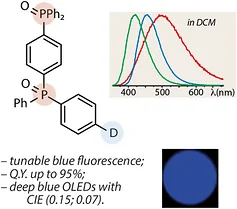

## 1 Introduction

Pentavalent phosphorus derivatives, particularly λ^5^σ^4^‐phosphane oxides (i.e., R_3_P = O, where *λ* and *σ* denote the valence and coordination numbers of the phosphorus center, respectively), have emerged as highly versatile structural motifs in functional organic materials, as the polarized P═O bond simultaneously imparts strong electron‐withdrawing character, high thermal stability, and a rigid arrangement [1, 2, 3]. These attributes enable precise control over frontier molecular orbitals, electron affinity, and molecular packing, thereby mitigating aggregation‐induced quenching pathways that often limit device performance. Consequently, phosphane‐oxide units are now widely employed across modern optoelectronic technologies and energy‐related applications, [4, 5, 6, 7, 8] serving as electron‐transporting and injection components, high‐triplet‐energy hosts, charge‐modulating substituents in donor–acceptor systems, and integral fragments of emissive chromophores [9, 10, 11, 12, 13, 14, 15]. Their dual role as both electronic regulators and structural spacers is particularly advantageous for organic light‐emitting diodes (OLEDs), where stability and precise energy‐level alignment are essential.

The growing prominence of phosphane oxides in OLEDs stems from their ability to simultaneously facilitate efficient electron transport and maintain high triplet energies. In host and electron‐transporting materials, the incorporation of P═O groups enhance electron mobility, and suppresses charge trapping while preserving sufficiently high triplet energies required for efficient blue and deep‐blue emission. Classical systems such as diphenylphosphane oxide‐based hosts (e.g., DPEPO) exemplify this behavior, enabling high‐performance phosphorescent and thermally activated delayed fluorescence (TADF) devices by preventing detrimental back energy transfer [16, 17]. Beyond host materials, the direct integration of phosphane oxide units into emissive frameworks has opened new directions in luminophore design [11, 13, 18, 19, 20]. Acting as strong acceptors, P═O fragments stabilize LUMO levels and promote intramolecular charge transfer, enabling fine‐tuning of excited‐state energies and, in favorable cases, enhancing reverse intersystem crossing (RISC) in TADF systems [18, 21, 22]. For instance, incorporation of a P═O unit into multi‐resonance emitters has led to record efficiencies approaching 37.6%, although the corresponding color purity (CIE_y_ ≈ 0.11–0.17) still falls short of the stringent deep‐blue requirements [23, 24, 25, 26].

On the other hand, the high degree of functionalization of the phosphorus center enables the construction of relatively simple donor–acceptor (D–π–A) and ambipolar (D–π)_n = 2‐3_–A architectures, combining synthetic accessibility with competitive device performance [27, 28, 29, 30]. Such molecules often deliver deep‐blue emission with CIE coordinates of (0.15, 0.05–0.07) while maintaining balanced charge transport. For example, carbazole–phosphane oxide host materials exhibit excellent ambipolar transport and high triplet energies, ensuring efficient exciton confinement and reduced device degradation [31, 32]. More elaborate ambipolar designs, such as (D–π)_3_–A systems, achieve true‐blue emission (CIE ≈ 0.15, 0.06) with EQEs up to 6.5% in non‐doped OLEDs, whereas simpler linear D–π–A phosphane oxides can function both as emitters (EQE ≈ 5.4%, CIE 0.15, 0.06) and as hosts in phosphorescent devices (EQE up to 18.1%) [33]. Furthermore, optimized linear systems incorporating extended π‐spacers, such as pyrene–carbazole derivatives, have demonstrated pure deep‐blue OLEDs with EQEs as high as 11.2%, attributed to efficient exciton harvesting via higher‐lying triplet states [30]. Importantly, recent studies highlight that not only donor–acceptor strength but also connectivity and molecular symmetry govern the excited‐state behavior; asymmetric spiro‐configured systems, for example, exhibit improved charge balance and reduced quenching compared to their symmetric counterparts [19].

Building on these insights, we posit that breaking the conventional symmetry of linear D–π–A phosphane oxide systems could provide an additional, to date underexplored, handle to modulate charge distribution and excited‐state dynamics. Thus, this work introduces a new class of asymmetrically engineered luminophores featuring an acceptor–(phenylene)–acceptor–(π‐spacer)–donor (A–π–A–π′–D) architecture (*Γ*–type), in which two phosphane oxide units serve as intermediate and terminal electron‐accepting centers.

## 2 Results and Discussion

### 2.1 Synthesis and Characterization

**SCHEME 1:**
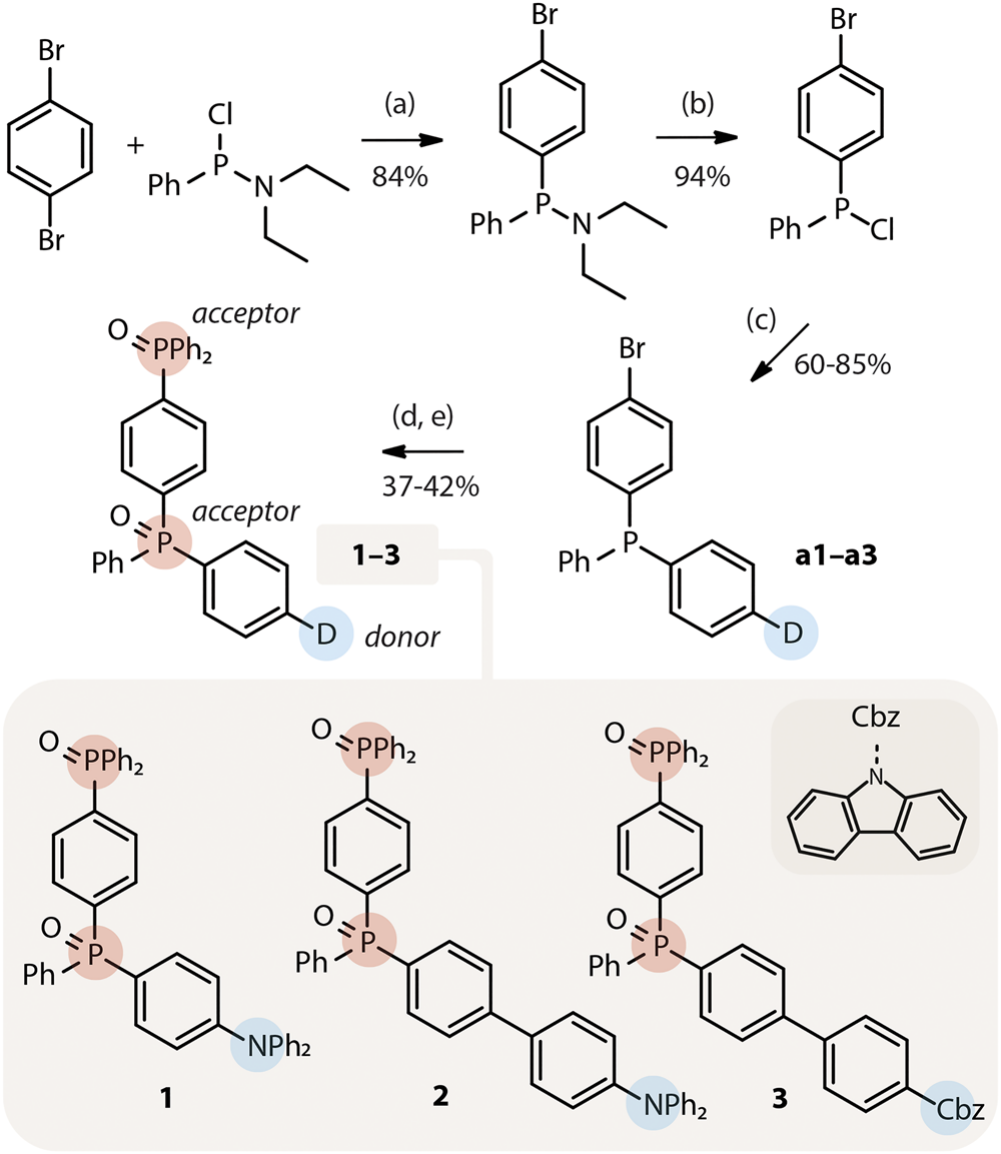
Synthetic route to diphosphane dioxides **1**–**3**. (a) *n*‐BuLi 2.5 M, THF, −85°C, 1 h, *then* PPhCl(NEt_2_), −85°C → rt, 1.5 h; (b) HCl, diethyl ether, 0°C, 2 h, distillation (10 mbar, 185°C); (c) *p*‐BrC_6_H_4_NR_2_ + *n*‐BuLi 2.5 M, THF, −78°C → −60°C, 1 h *then* (*p*‐BrC_6_H_4_)PhPCl, −78°C → rt, 1 h; (d) *i*. *n*‐BuLi 2.5 M, THF, −78°C, 1 h, *then* PPh_2_Cl, −78°C → rt, 1 h; *ii*. H_2_O_2_ (30% aq. solution), CH_2_Cl_2_, 2 h.

The target diphosphane dioxides **1–3** were synthesized through a four‐step sequence outlined in Schemes 1 and S1 (see Supporting Information). First, mono‐lithiation of 1,4‐dibromobenzene in diethyl ether was followed by the treatment with *N,N*‐diethyl‐*P*‐phenylphosphonamidous chloride [34] to afford *N,N*‐diethyl‐*P*‐(4‐bromophenyl)‐*P*‐phenylphosphinous amide as a viscous oil in 84% yield. Subsequent exposure to anhydrous HCl in diethyl ether (1.8 M) followed by vacuum distillation (10 mbar, 185°C) produced (4‐bromophenyl)phenylphosphino chloride in nearly quantitative yield. Then, a bromoaryl substrate (aryl = benzene, biphenyl) bearing a donor group (D = diphenylamine or carbazole) was lithiated in THF at –78°C with *n*‐BuLi and coupled with the chlorophosphane precursor, furnishing the corresponding mono‐phosphanes **a1–a3** in 60%–85% yields. These intermediates were used without extensive purification. Phosphanes **a1–a3** were lithiated under the aforementioned conditions and reacted with diphenylphosphino chloride to provide the diphosphane intermediates, which were oxidized using an excess of hydrogen peroxide (10 equiv., 30% aq.). Final purification by column chromatography afforded diphosphane dioxides **1–3** in satisfactory 37%–42% yields.

The purity and identity of diphosphane dioxides **1**–**3** were confirmed by multinuclear NMR spectroscopy (^1^H, ^13^C{^1^H}, ^31^P{^1^H}, and ^1^H–^1^H COSY), elemental analysis (see Supporting Information for details). While each of the phosphanes **a1–a3** show single resonance in the range from −7.7 to −8.1 ppm in their ^31^P{^1^H} spectra, diphosphane dioxides **1–3** feature significantly deshielded signals around 27.2–27.5 ppm represented as doublets with a scalar ^5^ *J*_PP_ coupling constant of ∼ 3.5–3.6 Hz.

### 2.2 Photophysical Properties

**FIGURE 1:**
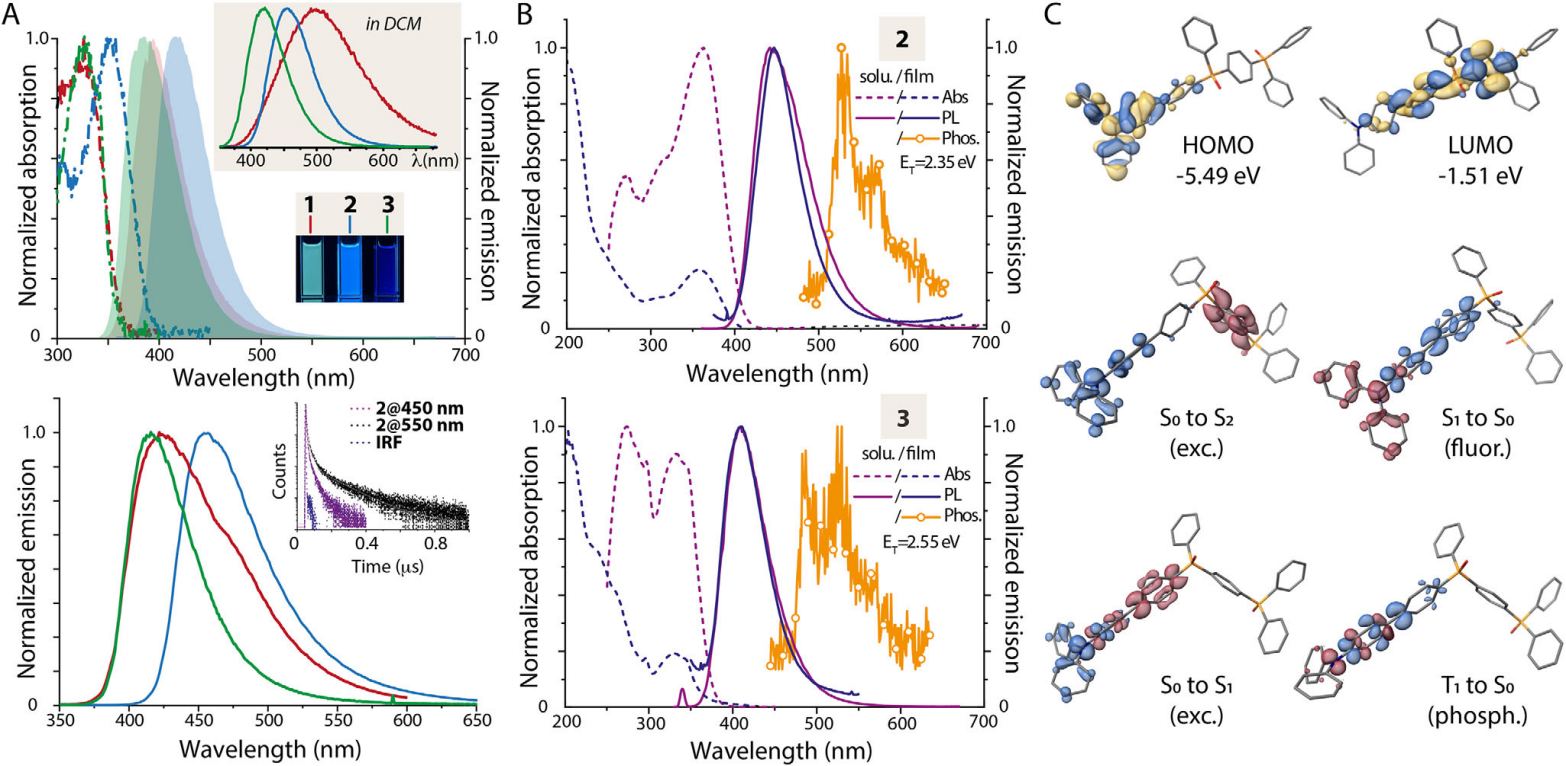
(A) Up – normalized absorption and emission profiles of **1** (red), **2** (blue) and **3** (green) in toluene solutions (*ca*. 10^−5^ M), the inset shows DCM solutions of **1**–**3** under 365 nm irradiation and their emission profiles; bottom – emission of **1**–**3** in the solid‐state, the inset shows decay profiles of **2** monitored at 450 nm and 550 nm (bottom); (B) Absorption and emission profiles of **2** and **3** measured for DCM solutions (297 K) and neat films (297 and afterglow measured at 10 K); (C) HOMO/LUMO orbitals, excitation (S_0_→S_1_/S_2_), singlet (S_1_→S_0_) and triplet emission (T_1_→S_0_) electron density difference plots for **2** (loss electron density: blue, gain: red).

The steady‐state and time‐resolved data of **1**–**3** in diluted solutions (*ca*. 10^−5^ M), solids, and neat films are shown in Figures 1, S1–5, and summarized in Tables 1 and S1–7.

**TABLE 1:** Photophysical data for samples **1**–**3** in solution, solid, and thin films.

		*λ*_abs_ (nm)	*λ*_em_ (nm)	*τ*_obs_ (*A*_i_, %) (ns)	*τ*_av_ ^a^ (ns)	*Φ*	*k*_r_ ^b^ (10^7^ s^−1^)
**1**	DCM	328	480	13.2	—	0.19	1.4
	Toluene	325	395	10.6	—	0.30	2.8
	Solid	325	421	1.1 (81), 8.2 (18), 44.2 (1)^425 nm^ 1.8 (67), 12.7 (32), 87.8 (1)^500 nm^	2.8^425 nm^ 6.2^500 nm^	0.08	—
**2**	DCM	352	456	3.6	—	0.95	26.4
	Toluene	353	418	3.6	—	0.82	22.8
	Solid	350	457	1.0 (87), 5.9 (13), 36.7 (1)^450 nm^ 2.8 (86), 15.6 (13), 150.7 (2)^550 nm^	2.0^450 nm^ 6.0^550 nm^	0.47	—
	Neat film	355	447 (532)^c^	—	—	0.64	—
**3**	DCM	327	420	3.7	—	0.95	25.7
	Toluene	327	385	3.5	—	0.95	27.1
	Solid	335	415	1.1 (84), 5.4 (15), 35.2 (1)^420 nm^ 3.1 (89), 15.2 (9), 124.4 (2)^520 nm^	2.1^420 nm^ 6.7^520 nm^	0.33	—
	Neat film	330	411 (520)^c^	—	—	0.54	—

^a^ Amplitude‐weighted average lifetime, calculated from *τ*_av_ = Σ*A*_i_ *τ*_i_, *A*_i_ = weight of the *i*‐th exponent.

^b^ k_r_ = τ_av_/*Φ*.

^c^ Time‐gated emission maxima obtained at 10 K.

In toluene, samples **1–3** exhibit intense low‐energy charge‐transfer (CT) absorption bands peaking at ca. 325, 353, and 327 nm (ε = 16.7–39.0×10^4^ M^−1^cm^−1^), respectively, in agreement with structurally similar (am)bipolar species [33, 35]. The red shift observed for **2** relative to **1** originates from the increased π‐conjugation length (phenylene → biphenylene), which raises the HOMO and lowers the LUMO energy (Figure S6) while the hypsochromic shift of **3** compared to **2** is attributed to the weaker electron‐donating character of carbazolyl vs diphenylamino moiety. Similar absorptions with maxima at ca. 328, 352, and 327 nm were observed in dichloromethane (DCM), indicating that the CT bands show negligible sensitivity to solvent polarity (Figure 1A,B). Our (TD‐)DFT calculations confirmed the expected CT character of the lowest lying singlet excited states (Figures 1 and S7–9), primarily showing transfer of electron density from the amine donor unit (diphenyl amine or carbazole) to the π* orbitals of a P = O‐decorated phenylene spacer. For **1**, the CT occurs between the donating moiety and the phenylene, which is anchored by both phosphane oxide groups, thereby providing almost perpendicular orientation of donor and acceptor (Figure S7). In contrast, the accepting phenylene is directly adjacent to the donor in **2** and **3** (Figures 1 and S8). Moreover, the S_1_ transitions of **2** and **3** have a small admixture of the locally excited (LE) state, which is absent for **1**.

In solution, samples **1**–**3** display pronounced solvent‐dependent photoluminescence characteristics of the CT excited state (Figures 1, S1–3). The emission of **1**–**3** in toluene mirrors the shape of the CT absorption and peaks at 395, 418, and 385 nm, respectively (Figure 1A). In DCM, the emission maxima are significantly red‐shifted, the compounds emit broadly between 420–480 nm, with **1** showing the most red‐shifted emission (480 nm). Notably, **1** also exhibits substantial band narrowing upon moving to the less polar medium: its FWHM decreases from *ca*. 5350 cm^−1^ in DCM to *ca*. 3500 cm^−1^ in toluene. In contrast, the emission bands of **2** and **3** are considerably narrower and show little or no solvent dependence, with FWHM values consistently in the range of *ca*. 3200–3700 cm^−1^ in both DCM and toluene. The significant solvent‐dependent FWHM observed for **1** is likely attributable to the larger changes of dipole moment upon excitation, making the excited state sensitive to the local electric‐field environment. Support of this viewpoint is given by the TD‐DFT calculations, the results of which revealed very small dipole moments (μ) for **1**–**3** at ground state geometries being 1.4, 0.8, and 2.4 D, respectively. In contrast, dipole moments at S_1_ geometries are substantially higher reaching 34.2 (**1**), 20.6 (**2**), and 20.4 D (**3**), resulting in polarized configurations, which are more stabilized by solvents of higher polarity, thereby red shifting the *λ*_max_. These considerations are in line with experimentally obtained trend for Δμ derived from Lippert‐Mataga plots (Figures S1–3), which deliver larger values for **1** (36.4 D) compared to those for **2** (26.8 D) and **3** (27.2 D). The largest increase in dipole moment calculated for **1** aligns with the strongest positive solvatochromism for this compound. The highest μ* for **1** is likely attributed to a more significant redistribution of electron density upon excitation (Figure S10) due to the nearly perpendicular orientation of donor and acceptor (*vide supra*).

For **2** and **3**, the monoexponential decays afforded excited state lifetimes in the 3.5–3.7 ns range in both solvents. Combined with near‐unity photoluminescent quantum yields (*Φ* ≈ 0.95), the large estimated radiative rate constants *k*_r_ ≈ 2.28–2.78 × 10^8^ s^−1^ are consistent with prompt emission from the singlet excited state. Noticeable elongation of the lifetime to 13.2 ns, alongside reduced quantum yields (*Φ*_toluene_ = 0.30, *Φ*_DCM_ = 0.19), was observed for **1**, leading to *k*_r_ of the order of 10^7^ s^−1^, still indicative of a prompt fluorescence. The elongated excited‐state lifetime reflects a reduced oscillator strength (ƒ) for compound **1**. As listed in Tables S2–5, the ƒ value of the S_1_ state for **1** (1.2×10^−2^) is more than one order of magnitude lower compared to **2** and **3** (0.900 and 0.534, respectively), which suppresses the fluorescence efficiency of **1**. The underlying reason behind such different ƒ values is strongly decoupled electron and hole, which are separated by the phosphane oxide group for the S_1_ state of **1**. For **2** and **3**, however, the charge transfer occurs within the donating group and the nearest phenylene in S_1_, with a small LE admixture, leading to substantial electron/hole coupling that enhances the transition dipole moment and, consequently, the oscillator strength. It is important to note that the S_2_ state of **1** has a similar character (Figure S7), with an ƒ value as high as 0.651, and is only 0.13 eV from S_1_ (Table S2).

**FIGURE 2:**
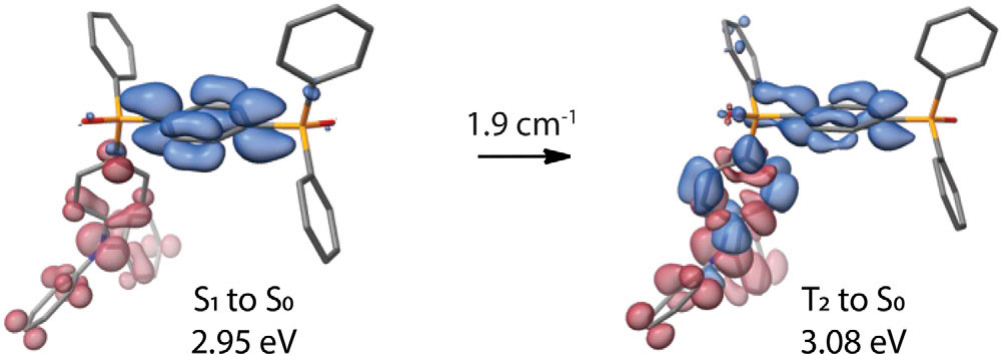
S_1_→T_2_ ISC channel of the **1**. Blue: loss of electron density (blue), red: gain.

Due to the elongated excited‐state lifetime recorded for **1**, the reduced quantum yield can be ascribed to non‐radiative deactivation from the singlet manifold (S_1_→S_0_); however, TD‐DFT predicted spin‐orbit coupling matrix elements (SOCME, Table S8) at the S_1_ state geometry point to accessibility of intersystem crossing (ISC). Specifically, the SOCME value between S_1_ and T_2_ states of 1.9 cm^−1^ (Figure 2) falls within the range calculated for benzophenone, which is known to undergo fast population of the triplet state [36].

As shown in Figure 2, the T_2_ state of **1** differs considerably from the CT configuration of the S_1_ due to a strong LE admixture. The principal difference between these two states, along with a small energy separation (Δ*E* = 0.13 eV), enables the population of the T_2_ state. Subsequently, prompt internal conversion to the T_1_ state and its relaxation yield almost pure LE excited state (Figure S7), leading to the large S_1_‐T_1_ energy gap of 0.6 eV (Tables S6,7) that prevents borrowing the oscillator strength from the singlet manifold and hinders efficient emission from the T_1_ state. However, triplet luminescence can be detected in frozen glass (Figure S11, Table S9). The obtained emission profile is vibrationally resolved and well matches the calculated T_1_ state (at the T_1_ geometry), which consists of a predominant LE transition localized on the donor moiety, as mentioned above. Moreover, the time‐resolved measurement revealed a long lifetime of hundreds of milliseconds, clearly demonstrating spin‐forbidden emission. Interestingly, the detected prompt fluorescence has a lifetime of only 2.35 ns, that is, much shorter compared to the room temperature data, which can be explained by quenching of the S_1_ excited state by the ISC process, thereby providing an approximate upper bound for the ISC rate of 4.3 × 10^8^ s^−1^. Although for **2** and **3** the TD‐DFT‐SOCME calculation revealed similar ISC channels, in these cases the population of the triplet manifold is less probable because of the high S_1_ oscillator strength and considerable gap between S_1_ and T_2_ states (up to 0.4 eV, see Table S6 for details), which makes prompt fluorescence more efficient. Indeed, although long‐lived phosphorescence can be detected in frozen glass, analogously as for **1** (Figure S11), it appears less intense, with emission spectra resembling CT transition with no signs of vibrational progression, which would be expected in the case of more intense phosphorescence due to the LE character of the T_1_ state.

In the solid state, **1**–**3** retain the same range of emission wavelengths observed in solution (*λ*_em_ = 421 nm for **1**; 457 nm for **2**; 415 nm for **3**), but their bands are slightly red‐shifted and substantially broader, reflecting an increase in inhomogeneous broadening and restricted molecular relaxation (Figure 1). Dioxide **1** exhibits the widest spectrum within this series and develops a distinct low‐energy shoulder, consistent with the presence of multiple conformers or local packing environments. Time‐resolved measurements indicate that the emission mechanism differs from that in solution: all three dioxides show multiexponential decays, implying contributions from more than one emissive pathway arising from multiple conformers and/or sites. For **2**, the observed decay monitored at 450 nm comprises components of 1.0 ns (87%), 5.9 ns (13%), and 36.7 ns (1%) with calculated *τ*_av_ = 2.0 ns, whereas monitoring at 550 nm yields longer components of 2.8 ns (86%), 15.6 ns (13%), and 150.7 ns (2%) and nearly tripled *τ*_av_ = 6.0 ns. The appearance of these relatively long‐lived decays found at lower energies suggests that the solid‐state emission may include certain phosphorescent contribution, likely facilitated by conformational heterogeneity and increased intersystem crossing in the rigid medium. The broadened spectra and extended lifetimes indicate a more complex excited‐state manifold in the solid state than in solution.

### 2.3 Chiro‐Optical Properties

**FIGURE 3:**
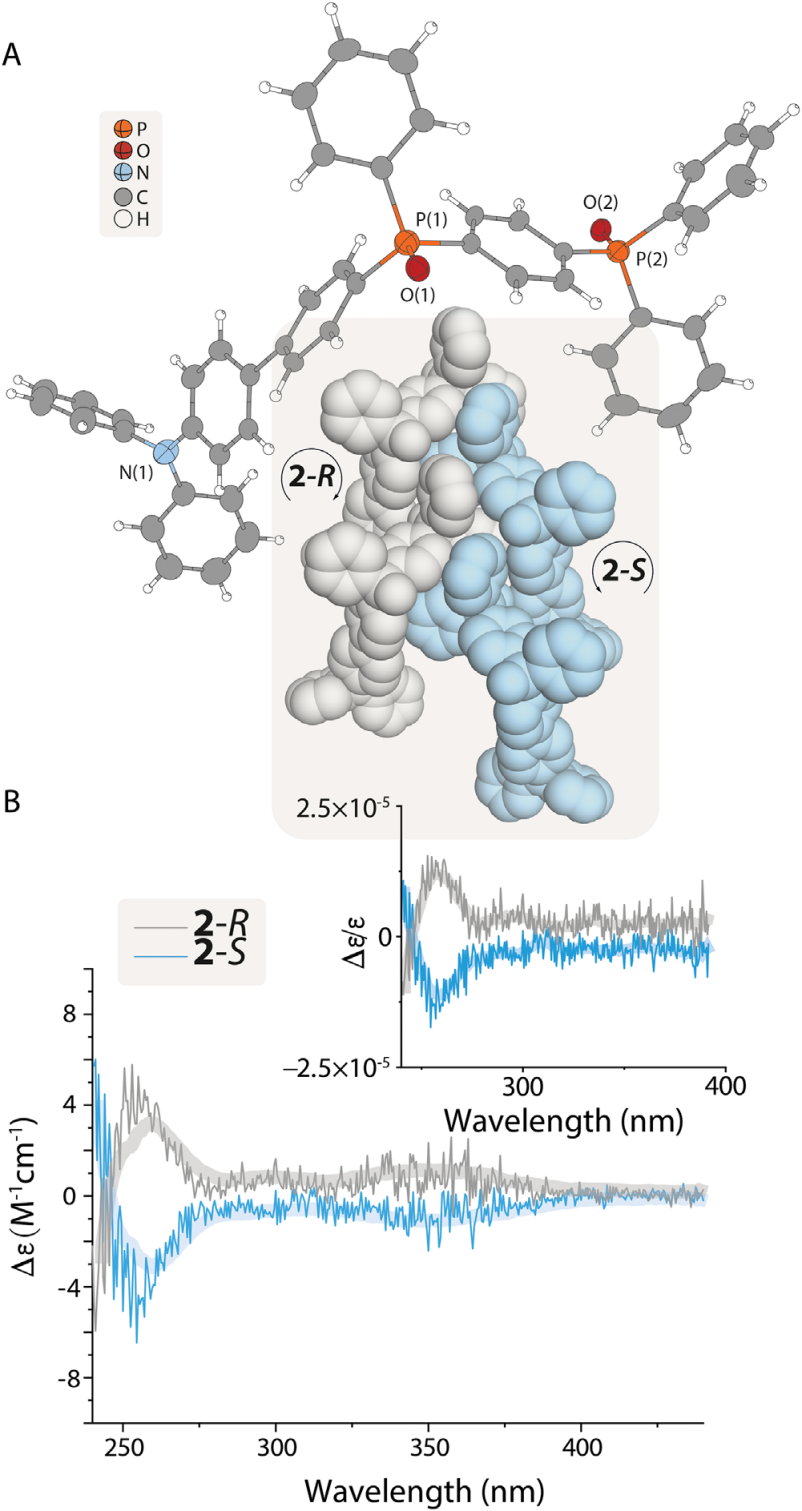
(A) Molecular view of diphosphane dioxide **2** [37]. The inset shows a fragment of a crystal packing that is composed of **2‐*R*** (gray) and **2‐*S*** (blue) enantiomers. Thermal ellipsoids are shown at the 50% probability level. (B) Electronic circular dichroism spectra of **2‐*R*** (gray) and **2‐*S*** (blue) enantiomers in DCM.

The scaffold of **2** was confirmed by single‐crystal X‐ray diffraction (scXRD); key structural parameters are summarized in Figure 3A and Table S10. The molecule adopts a unique *Γ*‐type (D–(π‐spacer)–A–(phenylene)–A) architecture, with the donor (D) and acceptor–acceptor (A–A) fragments oriented at the angle of 105.2° (P(2)–P(1)–N(1)). Importantly, scXRD confirms that the auxiliary pattern renders P(1) a stereogenic center, and both enantiomers (**2**‐***R*** and **2**‐***S***) are present in the crystal, forming infinite, symmetry‐related chains interconnected by multiple P═O⋯H─ and π(biphenyl)⋯H─C hydrogen bonds ranging from 2.377(6) to 2.750(1) Å (Figure 3A, inset), with no intermolecular π–π stacking interactions observed. The λ^5^σ^4^‐phosphorus centers are arranged in a trans fashion and display the expected tetrahedral geometry, with P─C and P─O bond lengths of 1.800(3)–1.819(2) Å and 1.485(2)/1.478(2) Å, respectively, consistent with reported values for phosphane oxides [38, 39, 40, 41].

Hence, owing to the intrinsic molecular chirality, racemic mixtures of **2** and **3** were subjected to enantiomeric separation using a Chiralpak IG column (hexane/ethanol/dichloromethane, 10:40:50 v/v/v) to evaluate their chiro‐optical properties (Figures S12,13). Enantiopure fractions **2‐*R/S*** and **3‐*R/S*** (ee > 99.5%) were obtained and subsequently examined by electronic circular dichroism (ECD) spectroscopy in DCM (Figures 3B, S14). Each pair of enantiomers displays mirror‐image ECD profiles consistent with their respective UV–vis absorption spectra in DCM. For **2‐*R/S***, the experimental ECD spectra show weak positive/negative Cotton effects at 255 nm (Δ*ε* = +4.5/−4.6 M^−1^ cm^−1^, |g_abs_| ≈ 1.4×10^−4^) and a smaller response for the CT (S_1_ → S_0_) transition at 350 nm (Δε = +1.3/−1.6 M^−1^ cm^−1^, |g_abs_| ≈ 4.2×10^−5^). For **3‐*R/S***, the Cotton effects are slightly smaller, appearing at 255 nm (Δ*ε* = +3.3/−3.2 M^−1^ cm^−1^, |g_abs_| ≈ 0.8 × 10^−4^) and 316 nm (Δ*ε* = +1.0/−0.9 M^−1^cm^−1^, |g_abs_| ≈ 3.3 × 10^−5^). The circularly polarized luminescence (CPL) signals of **2‐*R/S*** and **3‐*R/S*** are extremely weak, with estimated |g_lum_| < 10^−5^ below the detection limit of our CPL‐spectrofluorometer (|g_lum_| ≈ 0.6–0.8 × |g_abs_|) [42]. The excellent quantum efficiency, hence, the high electric transition dipole moment, combined with the intrinsically low magnetic transition dipole moment of the organic scaffold, limits the desired CPL activity. In addition, the small dissymmetry factors arise from the limited contribution of the chiral fragment to the frontier molecular orbitals (Figures 1C and S8). Altogether, the above findings constrain rotational strength and suppress both the CD and CPL responses. Nevertheless, the observed behavior aligns well with current benchmarks for chiral phosphorus‐based luminophores [43, 44]. With the given intrinsically low chiral properties, the enantiopure materials of **2** and **3** are unlikely to provide meaningful advantages in OLED performance, circularly polarized electroluminescence or asymmetric photocatalysis.

**FIGURE 4:**
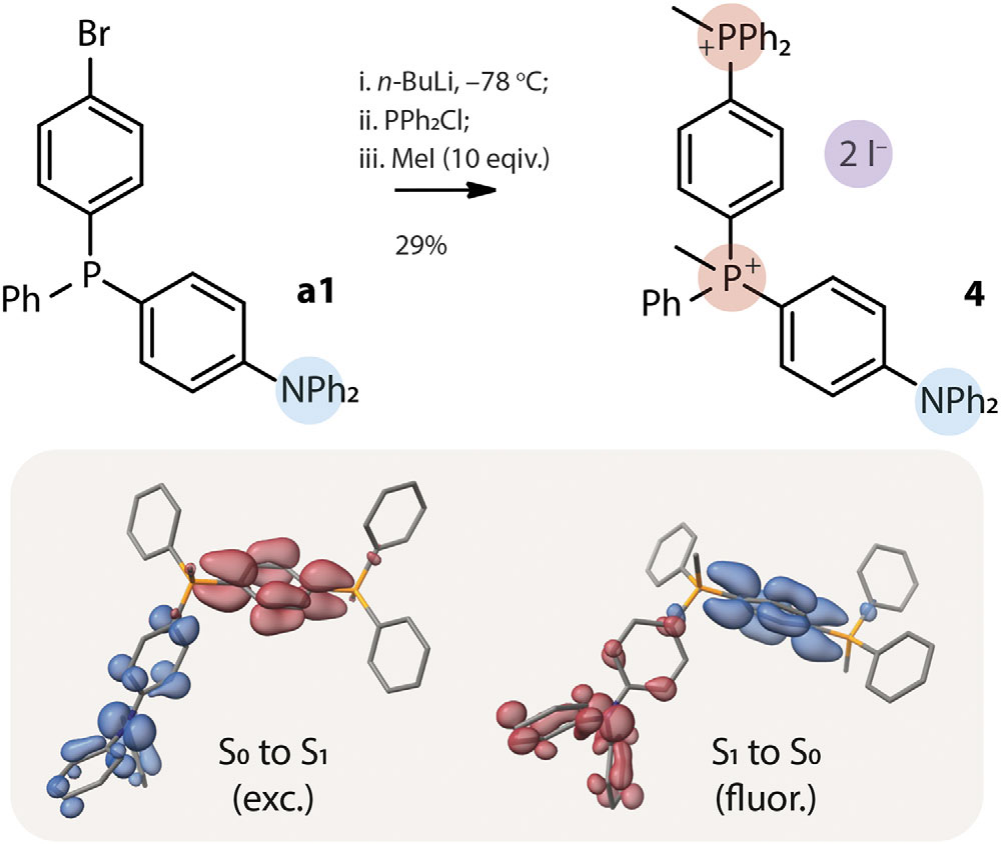
Synthetic access of the diphosphonium salt **4** (inset shows electron density difference plots for excitation (S_0_→S_1_) and emission (S_1_→S_0_) for **4** (loss electron density: blue, gain: red)).

Structural modifications such as the utilization of more electron‐demanding λ^4^σ^4^‐phosphonium groups (Figures 4, S6, S9, S15–16, Scheme S1, Table S5) instead of λ^5^σ^4^‐oxides may offer a route to enhance chiral perturbation by increasing orbital participation, as confirmed by our TD‐DFT and depicted in Figures 4 and S9. However, the obtained diiodide salt **4**, prepared via a similar synthetic route to **1** by using an excess of MeI instead of H_2_O_2_ (Figure 4), shows low photophysical performance with *Φ* < 0.01 (see Figure S15). Its poor fluorescence (S_1_→S_0_) is probably hampered by the high mobility of counterparts upon photoexcitation or ion pairing, which is especially pronounced for heavy iodide counterions, resulting in increased ISC (S_1_→T_1_) [45, 46, 47]. The enantioseparation of the racemic compound **4,** either via crystallization or chromatography, was not successful.

### 2.4 Electroluminescence Properties and Device Fabrication

Given their strong luminescence and accessible triplet states, **2** and **3** were selected as emitters for OLED fabrication. Thermogravimetric analysis revealed high thermal decomposition temperatures (*T*_d_, corresponding to 5% weight loss) of 475°C and 490°C for **2** and **3**, respectively (Figure S17), ensuring excellent stability during vacuum deposition. The absorption and photoluminescence (PL) spectra of vacuum‐deposited neat films on quartz substrates closely mirror their solution‐state behavior in dichloromethane (DCM) (Figure 1B), exhibiting slightly reduced PL quantum yields (*Φ*_PL_) of 64% and 54% for **2** and **3**, respectively. Time‐gated measurements of both films at 10 K revealed phosphorescence bands with triplet energies (*E*_T_), estimated from the emission onsets, of 2.35 and 2.55 eV for **2** and **3**. These values match those obtained from frozen DCM matrices, confirming that the triplet state appears at a molecular level rather than molecular packing or aggregation effects (Figure S11 and Table S9). Photoelectron yield spectroscopy was further conducted to obtain *E*_HOMO_ energy levels from thin neat films to emulate a solid‐state environment for constructing a correct device stack. Thus, the optical energy gap from absorption onset (*E*_g_) was used to calculate *E*_LUMO_ (*E*_HOMO_ + *E*_g_), delivering estimated *E*_HOMO_ */E*_LUMO_/*E*_g_ (eV) of −5.46/−2.44/3.02 for **2** and −5.72/−2.63/3.09 for **3**.

**FIGURE 5:**
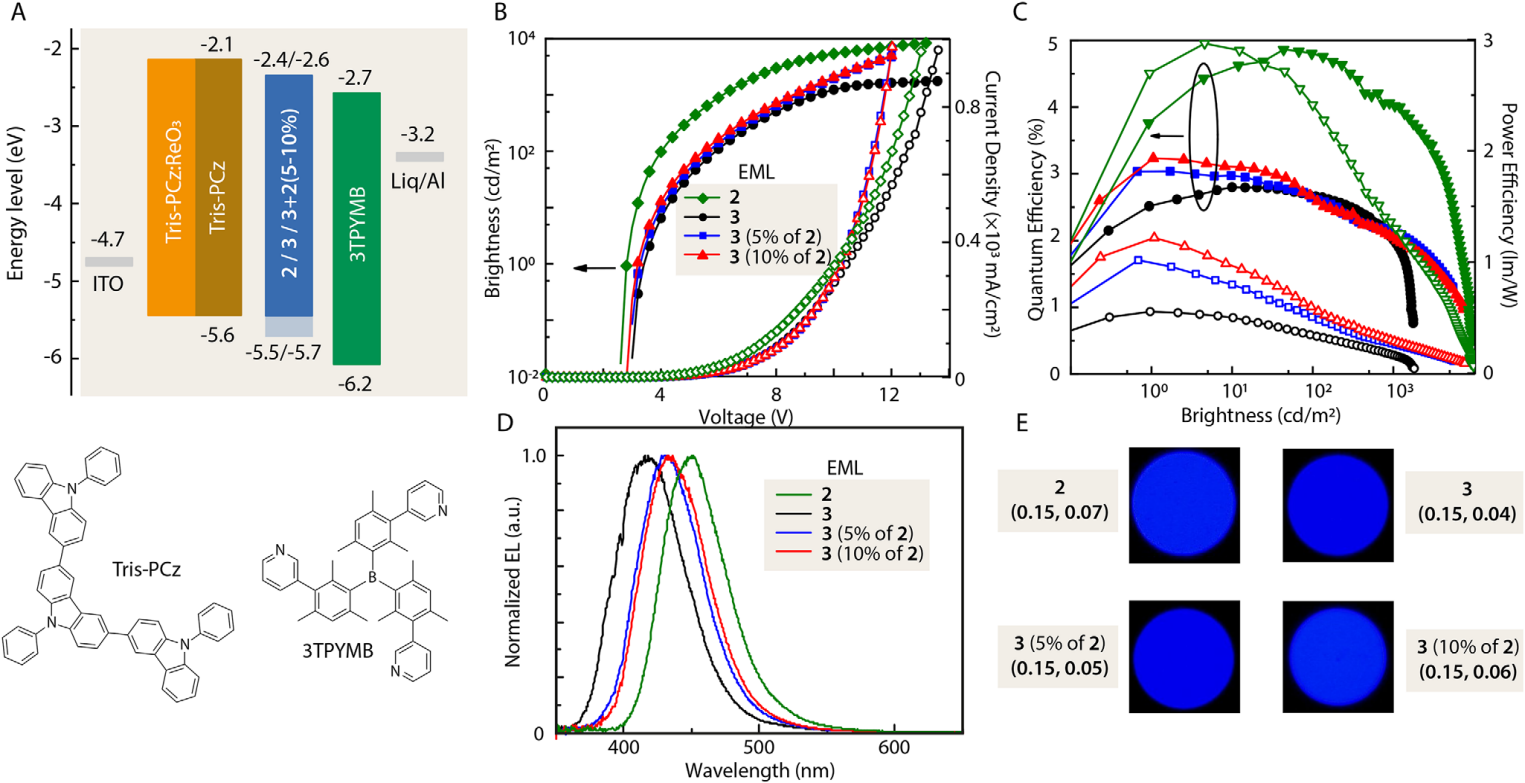
(A) Stack architecture of the OLED devices; (B) Current density–voltage–luminance (*J–V–L*) characteristics of obtained devices; (C) External quantum (EQE) and power efficiencies (PE) as a function of brightness; (D) Electroluminescence spectra of the devices; (E) Photographs of the appearance of OLED devices upon electrostimulation and their CIE color coordinates.

Initial non‐doped OLEDs were fabricated utilizing **2** as the emitting layer (EML) with the optimized configuration: ITO/Tris‐PCz: 4wt% ReO_3_ (60 nm)/Tris‐PCz (15 nm)/**2** (25 nm)/3TPYMB (X nm)/Liq/Al, where the thickness (X) of the electron‐transporting layer (ETL), tris(2,4,6‐trimethyl‐3‐(pyridin‐3‐yl)phenyl)borane (3TPYMB), was varied among 25, 35, and 50 nm (Figures 5 and S18). All devices exhibited a low turn‐on voltage (V_on_) of 2.8 V, reflecting efficient charge injection and well‐aligned energy levels throughout the stack. Increasing the 3TPYMB thickness from 25 to 35 nm leads to a pronounced improvement in device power efficiency (PE) from 2.1 to 2.71 lm/W, with the EQE_max_ rising from 4.45% to 4.86% (or from 4.37% to 4.80% at 100 cd/m^2^, respectively). This enhancement is attributed to improved electron transport and more balanced charge recombination within the EML. Further increasing the ETL thickness to 50 nm slightly reduced the EQE_max_ to 4.80%. Consequently, the 35 nm ETL represents the optimal thickness, yielding the highest PE and sustaining excellent efficiencies at practical operating voltages (3.8–4.0 V). Furthermore, the device utilizing **2** exhibited remarkable operational stability, with an efficiency roll‐off of only 17% as the luminance increased from 100 to 1000 cd/m^2^. The obtained EQE is higher by 1.5% than that reported for similar molecules with a *Γ*–type architecture, in which the intermediate acceptor is phenanthroline imidazole, pointing to a beneficial role of the secondary phosphane oxide group [48].

Concurrently, varying the 3TPYMB thickness enabled subtle, well‐controlled tuning of the electroluminescence color. The Commission Internationale de l'Éclairage (CIE) coordinates shifted from (0.15, 0.05) for the 25 nm ETL device to (0.15, 0.07) for the 35 and 50 nm devices, with the *x*‐coordinate remaining constant. This modest increase in the *y* ‐value points to minor alterations in the optical field distribution and recombination zone position, permitting fine color adjustment while preserving high‐purity deep‐blue emission without altering the emissive species.

To systematically compare the emitters, a second set of devices was fabricated with a fixed 35 nm ETL: ITO/Tris‐PCz:ReO_3_ (60 nm)/Tris‐PCz (15 nm)/Y (25 nm)/3TPYMB (35 nm)/Liq/Al (Table 2 and Figure 5). The device employing **3** exhibited comparatively lower performance, achieving a maximum luminance of 1766 cd/m^2^ and an EQE_max_ of 2.79%. In addition, doping **2** into the **3** matrix significantly enhanced the device characteristics; at doping concentrations of 5% and 10%, the EQE_max_ improved to 3.03% and 3.23%, respectively.

**TABLE 2:** Electro‐luminescent properties of the fabricated blue OLED devices.

X/Y	V_on_ (V)	*L*_max_ (cd/m^2^)	*I*_max_ (mA/cm^2^)	*η*_ext_ max (%, cd/A)	*η*_p_ max (lm/W)	At 100 nit (%, V)	CIE (x, y)
ITO/Tris‐PCz:ReO_3_(60 nm)/Tris‐PCz(15 nm)/**2**(25 nm)/**3TPYMB(X nm)**/Liq/Al							
25 nm	2.8	6517 (at 13.6 V)	993	4.45, 2.3	2.10	4.4, 3.8	(0.15, 0.05)
35 nm	2.8	8372 (at 13.2 V)	1038	4.86, 3.1	2.71	4.8, 4.0	(0.15, 0.07)
50 nm	2.8	7443 (at 14.0)	1033	4.80, 2.99	2.47	4.75, 4.3	(0.15, 0.07)
ITO/Tris‐PCz:ReO_3_(60 nm)/Tris‐PCz(15 nm)/**Y(25 nm)**/3TPYMB(35 nm)/Liq/Al							
**3**	3	1766 (13.6 V)	968	2.79, 0.66	0.45	2.00, 9.4	(0.15, 0.04)
**3**+**2**(5%)	2.8	4742 (at 12.0 V)	974	3.03, 1.03	0.95	2.13, 8.8	(0.15, 0.05)
**3**+**2**(10%)	3	4993 (at 12.0)	978	3.23, 1.24	1.22	2.05, 8.6	(0.15, 0.06)

Notably, the optimal EQE of the non‐doped device based on **2** (4.86%) is nearly double that of the device based on **3** (2.79%). To rationalize these unexpectedly high efficiencies for deep‐blue fluorescent OLEDs, we evaluated the parameters governing the EQE equation: *η*_EQE_ = *γ* × *Φ*_PL_ × *η*_out_ × *η*_ST_, where *γ* is the charge balance factor, *Φ*_PL_ is the PL quantum yield of the emitter, *η*_out_ is the light out‐coupling efficiency, and *η*_ST_ is the radiative exciton fraction [49]. To evaluate the charge‐carrier transport properties of the dioxides **2** and **3** (which govern *γ*), single‐carrier transport hole‐only devices (HODs) and electron‐only devices (EODs) were fabricated with the respective configurations of ITO/PEDOT:PSS/Tris‐PCz (20 nm)/**X** (60 nm)/Tris‐PCz (20 nm)/Liq (0.1 nm)/Al and ITO/PEDOT:PSS/3TPYMB (20 nm)/**X** (60 nm)/3TPYMB (20 nm)/Liq (0.1 nm)/Al, respectively (**X** = **2** or **3**). The current density–voltage (*J–V*) characteristics (Figure S19) indicate that electron transport predominates in both materials, although **3** exhibits a more balanced electron and hole flux compared to **2**. This behavior aligns with findings from previously reported ambipolar multibranched monophosphane oxides [33, 35], which show the most balanced charge carrier injecting/transporting properties for monoxides, complemented by two‐ or three phenylene/biphenylene donor‐amine branches.

The *Φ*_PL_ of **2** and **3** in neat film were 64% and 54%, respectively. Furthermore, angle‐dependent PL (ADPL) intensity measurements were performed to determine the horizontal emitting dipole ratios (Θ), which critically influence *η*_out_ (Figure S20). The Θ values for neat films of **2** and **3** were 0.76 and 0.71, respectively. However, the observed Θ values are lower than those of state‐of‐the‐art highly oriented emitters (Θ > 90%) [50]. Therefore, neither light out‐coupling enhancements nor the observed Θ values can fully account for the obtained EQE of the device based on **2**.

**FIGURE 6:**
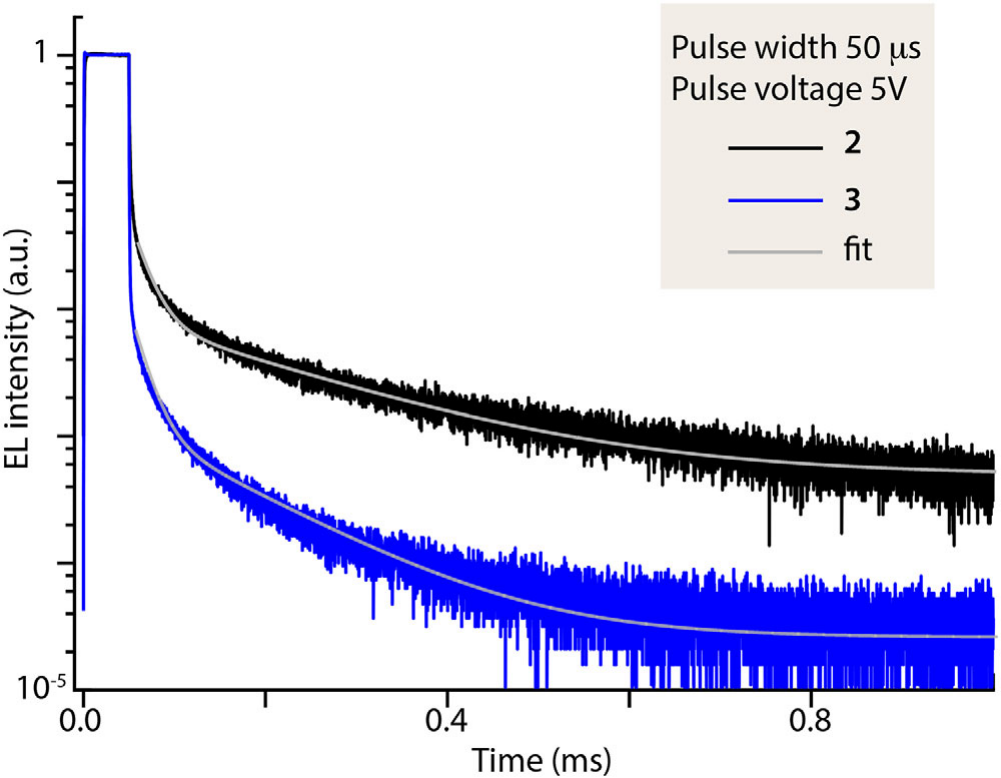
Transient electroluminescence signals for devices based on **2** and **3,** and their fits.

Although an EQE of the non‐doped device based on **2** (4.86%) sits just below the classical 5% upper limit for ideal conventional fluorescent OLEDs, it represents a noticeable enhancement given the photophysical constraints of the **2** and **3**. Considering the neat‐film *Φ* = 64% of **2**, a conventional fluorescent device limited by a 25% singlet exciton fraction would yield a maximum theoretical EQE of only ca. 3.2–4.8% (assuming *η*_ST_ = 20%–30%; using *η*_EQE_ = *γ* × *Φ*_PL_ × *η*_out_ × *η*_ST_). Thus, with the given singlet/triplet energy levels of the emitters (Figure 1), we hypothesized that the elevated EQE is primarily driven by supplementary singlet excitons generated via the triplet‐triplet annihilation (TTA) channel (enhancing *η*_ST_), as suggested by the photophysical data. Transient electroluminescence (TREL) is a highly effective technique for elucidating TTA dynamics in OLEDs by capturing the characteristic delayed fluorescence [51]. As shown in Figure 6, the TREL transient decays for devices based on **2** and **3** (recorded at 5 V with a pulse width of 50 µs) exhibit two distinct kinetic components. To eliminate the effect of trapped charges, a reverse‐pulse voltage (−3 V) was applied immediately after the forward‐pulse bias. The resulting EL decay profiles comprise a fast prompt component, originating from the rapid radiative decay of electrically generated singlet excitons, and a microsecond‐scale delayed component, which is a direct signature of TTA upconversion. Crucially, the delayed fluorescence contribution in the device based on **2** is substantially higher than that observed for **3**. Driven by this prominent TTA contribution, the device based on **2** achieves its EQE_max_ of 4.86%, significantly outperforming the 2.79% EQE_max_ of the device based on **3**.

## 3 Conclusion

In summary, we present a synthetically streamlined strategy for novel diphosphane‐dioxide‐based luminophores by integrating two phosphane oxide units within an asymmetric donor–π–acceptor–π′–acceptor framework. This design enables effective tuning of electronic structure while avoiding the complexity of conventional high‐performance deep‐blue emitters. The resulting diphosphane dioxides exhibit high thermal stability and strong charge transfer character of the fluorescence. Photophysical and theoretical analyses reveal that emission properties are governed by the balance between charge‐transfer and locally excited states. Molecule **1**, with pronounced orbital separation, shows reduced oscillator strength and efficiency, as a result of enhanced intersystem crossing, whereas **2** and **3** display improved electron–hole overlap, leading to near‐unity photoluminescence quantum yields and efficient prompt fluorescence. OLED devices based on **2** and **3** demonstrate efficient deep‐blue emission with EQE up to 4.86%, low turn‐on voltage (2.8 V), and limited efficiency roll‐off (17% as the luminance increased from 100 to 1000 cd/m^2^). The enhanced performance is attributed, in part, to triplet–triplet annihilation, which increases singlet exciton utilization beyond the statistical limit. In addition, solid‐state measurements indicate the presence of multiple emissive pathways, reflecting conformational heterogeneity and a more complex excited‐state landscape compared to solution. Overall, this work establishes asymmetrical diphosphane dioxide architecture as a viable and accessible platform for deep‐blue emitters. The combination of accessible synthesis, tunable photophysics, and competitive device performance provides a practical alternative to more elaborate molecular systems and offers clear guidelines for future material design.

## Conflicts of Interest

The authors declare no conflicts of interest.

## Supporting information



Experimental procedures, additional spectroscopic, crystallographic, and computational data. The authors have cited additional references within the Supporting Information [52, 53, 54, 55, 56, 57, 58, 59, 60, 61, 62, 63, 64, 65].
**Supporting File 1**: chem71232‐sup‐0001‐SuppMat.pdf.

**Supporting File 2**: chem71232‐sup‐0002‐Data.zip.

## Data Availability

The data that support the findings of this study are available from the corresponding author upon reasonable request.
