# Supplementary material for: Asymmetric Diphosphane Dioxides With A–π–A–π’–D Scaffolds for High‐Purity Deep‐Blue Luminescence
Source: Chemistry. 2026 Jun 10;32(36):e71232. doi: 10.1002/chem.71232 (PMC13602893; doi:10.1002/chem.71232)
Supplement: Supplementary file 1 [file CHEM-32-e71232-s001.pdf]

Supporting Information for

Asymmetric Diphosphane Dioxides with A-π-A-π′-D Scaffolds for High-Purity Deep-Blue Luminescence

Eetu Hakkarainen,[a] Zong-Ying Liu,[b] Jhon Sebastian Oviedo Ortiz,[c] Nicolas Vanthuyne,[d] Jeanne Crassous,[c] Ondrej Mrozek,[e] Fabien B.L. Cougnon,[f] Wen-Yi Hung,[g]* Pi-Tai Chou,[b]* Igor O. Koshevoy,[a] Andrey Belyaev[a]**

[a] Department of Chemistry and Sustainable Technology, University of Eastern Finland, Joensuu, 80101, Finland

[b] National Taiwan University, Department of Chemistry, Taipei 106, Taiwan

[c] CNRS ISCR-UMR 6226, Université de Rennes, 35000 Rennes, France

[d] Aix Marseille Univ, CNRS, Centrale Marseille UAR, 1739, FSCM, Chiropole Marseille, France

[e] Institute of Inorganic Chemistry of the Czech Academy of Sciences, Husinec-Řež 250 68, Czech Republic

[f] Department of Chemistry, Nanoscience Center, University of Jyväskylä, Jyväskylä, 40014, Finland

[g] Department of Optoelectronics and Materials Technology, National Taiwan Ocean University, Keelung 20224, Taiwan

E-mail: mrozek@iic.cas.cz; wenhung@mail.ntou.edu.tw; chop@ntu.edu.tw; andrei.beliaev@uef.fi

Table of Contents

**General comments, instruments, and procedures**.	4
**Synthesis**	8
**Figures S1–3.** Normalized absorbance and emission of **1**–**3** in different solvents and corresponding Lippert-Mataga plot.	13
**Table S1**. Photophysical characteristics of **1**–**3** in different solvents.	14
**Figures S4–5**. Time-resolved emission spectra of solids **1**–**3** monitored at different wavelengths.	15
**Figure S6**. Selected molecular orbitals of **1**-**4**.	17
**Figure S7–9.** Selected difference densities calculated for **1**, **3** and **4** at S_0, S_1, and T_1 geometries.	18
**Table S2–5.** Selected singlet and triplet excitations of **1–4** in the ground state geometry.	21
**Tables S6–7.** Energies of selected excited states of **1-4** at S_1/T_1 geometries.	25
**Table S8.** SOCME^{TOTAL} values between T_{1-5} and S_{0-5} states of **1-3** at S_1 geometry.	26
**Figure S10**. Superimposed coordinates of optimized S_0 (blue), S_1 (green) and T_1 (red) states of **1**.	27
**Figure S11**. Photoluminescence, fluorescence lifetimes, and phosphorescence lifetimes of frozen DCM solutions (77 K) of **1**–**3**.	27
**Table S9**. Summary of the photophysical properties of frozen DCM solutions of **1**–**3**.	28
**Table S10**. Crystal data and structure refinement for **2**.	29
**Figures S11-13**. Separation details of the **2/3-*S*** and **2/3-*R*** enantiomers.	30
**Figure S14**. Electronic circular dichroism spectra of **3-*R*** (red) and **3-*S*** (black) enantiomers in DCM.	33

Figure S15. Structure of **4** and its normalized absorption, excitation, and emission spectra in DCM solution. 33

Figure S16. ESI-MS of compound **4** and the calculated isotopic distribution for molecular cations. 33

Figure S17. TGA curves of compounds **1–3**. 34

Figure S18. Current density–voltage–luminance (*J–V–L*) characteristics of nondoped devices. External quantum (EQE) and power efficiencies (PE) as a function of brightness. EL spectra for non-doped device . 35

Figure S19. Current density versus voltage curves of the electron-only and hole-only devices for the compounds **2** and **3**. 36

Figure S20. Angle-dependent PL intensity of *p*-polarized light at peak emission for **2** and **3** compared to simulated profiles (lines) with a different ratio of horizontal dipoles (Θ), 36

Figures S22–33. Copies of ^{1}H, $^{31}P\{^{1}H\}$ and ^{1}H-^{1}H COSY NMR spectra. 37

Tables S11–22. Cartesian coordinates for **1**–**4**. 49

References 85

General comments

N,*N*-diethyl-*P*-phenylphosphonamidous chloride,[1] 4-bromo-(*N,N*-diphenyl)-aniline,[2] 4-bromo-4′-(*N,N*-diphenylamino)-biphenyl,[3] 9-(4′-bromo[1,1′-biphenyl]-4-yl)-9*H*-carbazole[4] were synthesized according to the published procedures. Tetrahydrofuran (THF), hexane and diethyl ether were distilled over Na-benzophenone ketyl under a nitrogen atmosphere before use. Other reagents and solvents were used as received. The solution ^{1}H, ^{31}P{^{1}H}, ^{13}C{^{1}H}, and ^{1}H–^{1}H COSY NMR spectra were recorded on Bruker Avance 400 and AMX-400, JEOL 500 spectrometers. Mass spectra were recorded on a Bruker micrOTOF 10223 instrument in the ESI^{+} mode. Microanalyses were carried out at the analytical laboratory of the University of Eastern Finland.

Single-crystal X-ray structure analysis (SCXRD): The single crystals of **2** (obtained by overlayering of solution **2** in dichloromethane with hexane) were immersed in a film of NVH mounted on a polyimide microloop (MiTeGen), transferred to a stream of cold nitrogen (Cryostream 700 cooling system by Oxford Cryosystems), and measured at a temperature of 150 K. The X-ray diffraction data were collected on a Rigaku XtaLAB Synergy R diffractometer with a HyPix-Arc 100 detector using mirror-monochromated radiation (INCOATEC microfocus sealed tube). The frames were integrated with the CrysAlisPro software package using a narrow-frame algorithm. The CrysAlisPro program package was used for cell refinements and data reductions. The structure was solved using the intrinsic phasing method [5,6] refined with OLEX2-1.5,[7] and visualized with the Mercury 2024.3.1 and Diamond 4.0 programs. A semiempirical absorption correction (SADABS) was applied to all data. All non-hydrogen atoms were refined anisotropically. Hydrogen atoms were included in the structure factor calculations. All Hydrogen atoms were assigned to idealized geometric positions. The crystallographic details are summarized in Table S10. CCDC **2505612** contains supplementary crystallographic data for this paper.

Thermogravimetric Analysis (TGA): TGA measurements were carried out using a Mettler Toledo TGA 2 instrument in the temperature range of 30–600 °C with a heating rate of 10 °C min^{-1}, under a nitrogen atmosphere (flow rate 50 ml min^{-1}). 70 µl aluminum oxide pans were used for the experiments. Before the experiments, the pans were tempered at 1000 °C for 10 minutes and kept in an exicator to remove and protect from humidity.

Photophysical Measurements: All measurements were performed under an inert of nitrogen. Before measurements, samples were dried under vacuum (10^{-3} mbar) and then exposed to a few drops of solvent related to a particular solvatomorph under an inert atmosphere or single crystals of the phase-pure sample were used. Both excitation and emission spectra of the samples were recorded on an Edinburgh Instrument FLS1000 spectrometer, equipped with a 450 W ozone-free Xenon arc lamp, double monochromators for the excitation and emission pathways, and red-sensitive photomultipliers

(PMT-980) as detectors. The excitation and emission spectra were corrected using the manufacturer's standard corrections for the excitation source's spectral power and the detector's sensitivity. The emission was collected at right angles to the excitation source with the emission wavelength selected using a double-grated monochromator and detected by the respective PMT. The quantum yields of the solid samples were measured using an integrating cryosphere in the presence of several drops of the solvent to maintain sample homogeneity. The luminescence lifetimes were measured using an EP-LED pulsed laser diode (377 nm) with the multichannel scaling (MCS) method. A pulsed xenon microsecond flashlamp (60 W) was used to obtain long-lived (> 1 ms) lifetimes. Variable data measurements were performed with Optistat DN cryostat, 77 K. Fluoracle and FAST spectrometer operating software and OriginPro 2025 10.2.0.188 were used for data analysis and processing.

Solvent-variable optical characteristics for **1−3** were analyzed using the Lippert−Mataga equation, which describes the dependence of the Stokes shift between the absorption and emission bands (ν_{St}, cm^{-1}) on the solvent polarity parameter (Δf):

$$\nu_{St} = \frac{2(\Delta\mu)^2}{4\pi\varepsilon_0}\frac{\Delta f}{hca_0^3} + const$$

$\Delta\mu = \mu_{es} - \mu_{gs}$ (D) is the difference between the dipole moments in the excited and the ground states, ε_0 is the permittivity of vacuum (8.85×10^{-12} C^2 J^{-1} m^{-1}), h is Planck's constant (6.63×10^{-34} J s), c is the velocity of light (3×10^{10} cm s^{-1}), a_0 is the radius of the Onsager cavity around the fluorophore (m). The Onsager radii for metalloligands were estimated from the DFT optimized structures as half of the distance between diphenylamine donor and the acceptor diphenylphosphane groups (7.8 Å for **1**, 8.7 Å for **2,** and 8.8 Å for **3**), which corresponds to the part of the molecule where charge separation takes place; Δf is the orientation polarizability (intrinsic solvent polarity), which is determined by the corresponding dielectric constant (ε) and refractive index (n):

$$\Delta f = \frac{\varepsilon - 1}{2\varepsilon + 1} - \frac{n^2 - 1}{2n^2 + 1}$$

Commission internationale de l'éclairage (CIE 1931) coordinates were calculated from photoluminescence data by using Origin 9.55b software pack.

The experimental values of HOMO levels were determined with a Riken AC-2 photoemission spectrometer (PES), and those of LUMO levels were estimated by subtracting the optical energy gap from the measured HOMO.

Electronic circular dichroism spectra (ECD, in M-1 cm-1) were recorded on a Jasco J-815 Circular Dichroism Spectrometer (IFR140 facility - Biosit - Université de Rennes 1).

Device fabrication and measurements

All materials were purified by vacuum sublimation before use. ITO-coated glass with a sheet resistance of 15 Ω square $^{-1}$ was cleaned with detergent, then underwent wet cleaning in an ultrasonic bath, beginning with deionized water, followed by acetone and isopropanol. After air plasma cleaning, the ITO glass was placed in a thermal evaporation chamber. The deposition rate was kept at ca. 1 - 2 Å s^{-1}. Subsequently, LiF was deposited at 0.1 Å s^{-1} and then capped with Al metal (ca. 5 Å s^{-1}) through shadow masking without breaking the vacuum. The J-V-L characteristics of the devices were measured simultaneously in a glovebox using a Keithley 6430 source meter and a Keithley 6487 picoammeter equipped with a calibration Si-photodiode. EL spectra were measured using a photodiode array (Ocean Optics USB2000+). The transient electroluminescence of OLEDs was measured with a photomultiplier tube PMT module (H10720, Hamamatsu) and time-correlated single photon counting (TCSPC) system (TimeHarp 260, PicoQuant) to record the signals. The devices were driven using an arbitrary function generator (Agilent 33220A) to generate appropriate excitation waveforms, with a forward bias of 5 V and a pulse width of 50 μs (the pulse was sufficiently long for the prompt EL to reach its steady-state intensity). During the signal collection of the delayed EL, reverse pulses (-3 V) were applied on the devices to prevent the residual charge carriers on device. In brief, time-resolved studies were performed using a time-correlated single photon counting (TCSPC) system (OB 900-L, Edinburgh) with the second harmonic generation (SHG, at 380 nm) of pulse-selected femtosecond laser centered at 760 nm (Tsunami and Model 3980 pulse picker, Spectra-Physics) as the photoexcitation light source.

Angle-Dependent PL Spectra Measurement and Simulation

The angular dependent *p*-polarized fluorescence intensity was measured in an attempt toanalyze the transition dipole moment of emitters. The experimental setup was composedof a motorized rotation stage, a fused silica-based half-cylindrical lens, a longpass filter tostop the excitation beam, a polarizer, and a fiber-guided spectrometer (Ocean Optics USB4000) to collect the polarized emission. A continuous-wave Nd:YAG laser (355 nm, 10 kHz) was used as the excitation source which was fixed at an incident angle of 45°. Thes ample was deposited on top of a quartz substrate and encapsulated under N2 atmosphere. Measurements were performed automatically using the programmed rotation stage. Intensity of the *p*-polarized emission was recorded from 0° to 90° in steps of 5°. The datawere analyzed using SETFOS 4.5 (Fluxim AG, Switzerland) to fit the ratio of horizontal dipoles (Θ). The optical constants(refractive index n and extinction coefficient k) of each organic layer in the device were analyzed using variable-angle spectroscopic ellipsometry (J. A. Woolam SE spectroscopic ellipsometer).

Computational details

The geometries used in the theoretical studies were obtained by optimizing molecules at the DFT level (or from the experimentally determined X-ray structure of **2**), followed by frequency calculations confirming that the obtained structures correspond to minima on the potential energy surfaces. Subsequently, TD-DFT and SOCME-TD-DFT calculations were performed to evaluate the electronic structure and selected photophysical properties, including theoretical absorption spectra, excited-state characters, transition dipole moments, and spin–orbit coupling matrix elements. Excited-state geometries were optimized starting from the corresponding ground-state equilibrium structures.

DFT and TD-DFT calculations were performed with the ORCA 6.1.0 software.[8](10.1002/wcms.1606) Geometry optimizations with tight convergence criteria and no geometric constraints were carried out using the PBE0 functional[9] with the def2-TZVP basis set.[10] Van der Waals interactions have been accounted for by employing Grimme-D3BJ empirical dispersion correction.[11,12] To accelerate calculations, the SARC/J auxiliary basis set was used together with the RI approximation and ZORA approximation to account for relativistic effects.[13] Solvent effects (DCM, ε = 9.08) were accounted for by the implicit solvent model CPCM.[14] The same level of theory, but with def2-SVP basis, was used for TD-DFT calculation of the first 20 singlet and triplet excited states. Electronic transition differences at isovalues of 0.001-0.0015 were prepared using the orca_plot module as implemented in ORCA 6.1.0. software and ChimeraX.[15] The values of $SOCME^{TOTAL}$ listed in Table S8 were calculated as:

$$SOCME^{TOTAL} = \sqrt{|X|^2 + |Y|^2 + |Z|^2}$$

where X, Y, and Z are components of $\langle T_n|\widehat{H_s}|S_n\rangle$ matrix elements obtained by TD-DFT calculation with ORCA 6.1.0 with the “DOSOC true” keyword.

Syntheses

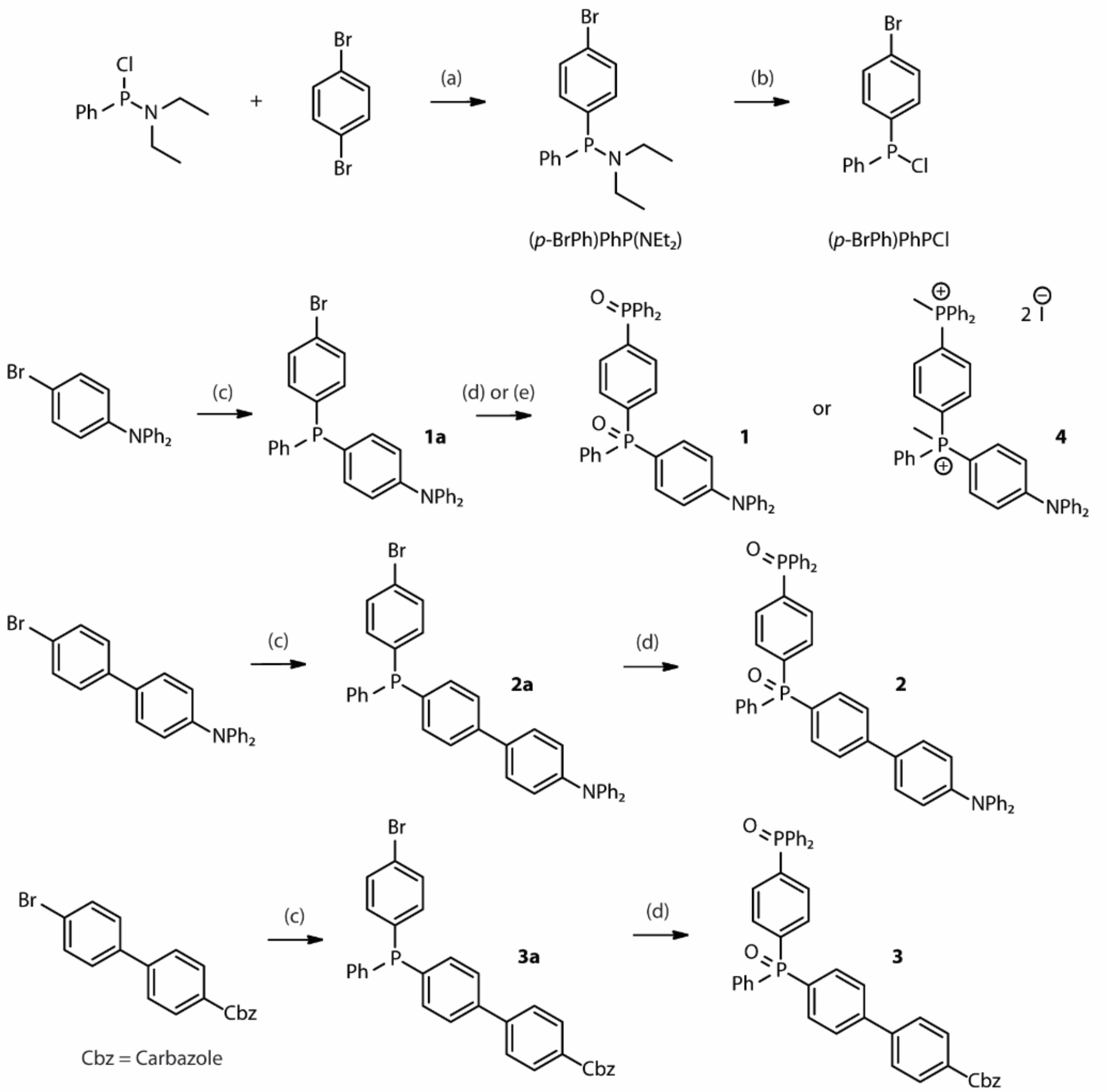

Scheme S1. Synthetic routes to diphosphane oxides **1**–**3** and phosphonium salts **4** and **5**: (**a**) *n*-BuLi 2.5 M, THF, –85 ºC, 1 h. *then* PPh_2Cl, –85 ºC → rt, 1.5 h; (**b**) HCl, diethyl ether, 0 ºC, 2 h; (**c**) *n*-BuLi 2.5 M, THF, –78 ºC → –60 ºC, 1 h *then* (*p*-BrPh)PhPCl, –78 ºC → rt, 1 h; (**d**) *i*. *n*-BuLi 2.5 M, THF, –78 ºC, 1 h *then* PPh_2Cl, –78 ºC → rt, 1 h; *ii*. H_2O_2 (30% aqueous solution), CH_2Cl_2, 2h; (**e**) *i*. *n*-BuLi 2.5 M, THF, –78 ºC, 1 h *then* PPh_2Cl, –78 ºC → rt, 1 h; *ii*. MeI, CH_2Cl_2, 2h.

***N*,*N*-diethyl-*P*-(4-bromophenyl)-*P*-phenylphosphinous amide ((*p*-BrPh)PhP(NEt_2))**. A solution of 1,4-dibromobenzene (10.30 g, 43.6 mmol) in THF (150 mL) was cooled to –85 ºC, and a 2.5 M solution of *n*-BuLi in hexanes (17.4 mL, 43.6 mmol) was added dropwise within 15 min. to give a white slurry. Then it was stirred at this temperature for 1 h. and treated dropwise with neat *N*,*N*-diethyl-*P*-phenylphosphonamidous chloride (9.40 g, 43.6 mmol). The obtained red solution was

allowed to reach room temperature and was stirred for additional 1.5 h. Then solvent was evaporated and organic part was extracted by freshly distilled hexane (5 × 40 mL) under nitrogen atmosphere. The solvent was evaporated to afford brown oily product (12.20 g, 84%). ^{1}H NMR ($CDCl_3$, 298 K; *δ*): 7.27–7.49 (m, Ph, 9H), 3.07 (dq, J_{HH} = 6.9 Hz, J_{HP} = 14.3 Hz -C$\underline{H}_2$$CH_3$, 6H), 0.96 (t, J_{HH} = 6.9 Hz, -CH_2C$\underline{H}_3$, 6H). ^{31}P{^{1}H} NMR ($CDCl_3$; 298 K; δ): 59.6 (s, 1P).

(4-bromophenyl)phenylphosphino chloride ((*p*-BrPh)PhPCl). HCl (30 mL, 54.0 mmol, 1.8 M in diethyl ether) was slowly added to an etheral solution (80 mL) of *N*,*N*-diethyl-*P*-(4-bromophenyl)-*P*-phenylphosphinous amide (12.20 g, 36.4 mmol) at 0 °C. The white suspension was stirred for 2 h. at room temperature, and then the precipitates were filtered off and washed with diethyl ether (5 × 40 mL). Combined organic layers were concentrated under vacuum, and the oily product was distilled (10 mbar, 185 °C) to afford transparent oil (10.12 g, 94%). ^{1}H NMR ($CDCl_3$, 298 K; *δ*): 7.43–7.61 (m, Ph, 9H). ^{31}P{^{1}H} NMR ($CDCl_3$; 298 K; δ): 79.3 (s, 1P).

General procedure for the preparation of a1–a3. The synthesis was carried out under a nitrogen atmosphere. A solution of a corresponding bromoaryl (1.00 eq.) in THF was cooled to –78 °C, and a solution of *n*-BuLi (2.5 M in hexanes, 1.05 eq.) was slowly added dropwise within 15 min. to give a clear colorless solution. The resulting mixture was stirred for 1 h. at –60 °C, then it was cooled to –78 °C and treated dropwise with neat (*p*-BrPh)PhPCl (1.00 eq.). The reaction mixture was allowed to reach room temperature and was stirred for an additional 2 h. Then it was quenched with methanol (15 ml) and evaporated. The solid residue was washed with methanol (3 × 15 ml) and dried *in vacuo*. Crude phosphane was passed through a pad of Silica gel (70-230 mesh, eluent dichloromethane-hexane, 1:1 v/v) and dried *in vacuo* to afford a white solid. Prepared phosphane was used in the next step without additional purification.

(4-bromophenyl)(4-(*N*,*N*-diphenylamino)phenyl))phenylphosphane, (**a1**) Prepared from 4-bromo-(*N,N*-diphenyl)-aniline (1.15 g, 3.56 mmol), *n*-BuLi (1.50 ml, 3.74 mmol) and (*p*-BrPh)PhPCl (1.07 g, 3.56 mmol) in THF (35 ml). White solid (1.12 g, 62%). ^{1}H NMR ($CDCl_3$, 298 K; *δ*): 7.47 (d, J_{HH} = 6.9 Hz, 2H), 7.25–7.37 (m, 9H), 7.12–7.21 (m, 8H), 7.07 (t, J_{HH} = 7.9 Hz, 2H), 7.02 (d, J_{HH} = 6.9 Hz, 2H). ^{31}P{^{1}H} NMR ($CDCl_3$; 298 K; δ): –8.1 (s, 1P).

(4′-(*N*,*N*-diphenylamino)-[1,1′-biphenyl]-4-yl)((4-(bromophenyl))phenyl)phenylphosphane (**a2**). Prepared from 4-bromo-4′-(*N,N*-diphenylamino)-biphenyl (1.27 g, 3.18 mmol), *n*-BuLi (1.36 ml, 3.40 mmol) and (*p*-BrPh)PhPCl (0.95 g, 3.18 mmol) in THF (30 ml). White solid (1.11g, 60%).^{1}H NMR ($CDCl_3$, 298 K; *δ*): 7.57 (d, J_{HH} = 7.9 Hz, 2H), 7.47–7.50 (m, 4H), 7.27–7.37 (m, 11H), 7.14–7.23 (m, 8H), 7.06 (t, J_{HH} = 7.3 Hz, 2H). ^{31}P{^{1}H} NMR ($CDCl_3$; 298 K; δ): –7.7 (s, 1P).

(4′-(9*H*-carbazol-9-yl)-[1,1′-biphenyl]-4-yl)(4-(bromophenyl))phenylphosphane (**a3**) Prepared from 4-bromo-4′-(carbazol-9-yl)-biphenyl (1.50 g, 3.77 mmol), *n*-BuLi (1.60 ml, 3.96 mmol) and (*p*-BrPh)PhPCl (1.13 g, 3.77 mmol) in THF (60 ml). White solid (1.86 g, 85%). ^{1}H NMR ($CDCl_3$, 298 K; *δ*): 8.18 (d, J_{HH} = 8.6 Hz, 2H), 7.82–7.84 (m, 2H), 7.63–7.69 (m, 5H), 7.30–7.50 (m, 16H). ^{31}P{^{1}H} NMR ($CDCl_3$; 298 K; δ): –7.7 (s, 1P).

General procedure for the preparation of 1–3.

(***i***). The synthesis was carried out under a nitrogen atmosphere. A solution of a **a1–a3** (1.00 eq.) in THF was cooled to –78 ºC, and *n*-BuLi (2.5 M in hexanes, 1.10 eq.) was slowly added dropwise within 15 min. to give a clear colored reaction mixture. The resulting solution was stirred at this temperature for 1 h, then it was treated dropwise with neat PPh_2Cl (1.05 eq.). The reaction mixture was allowed to reach room temperature and was stirred more for 1 h, quenched with methanol (15 ml), evaporated and the solid residue was washed with methanol (3 × 15 ml), diethyl ether (2 × 10 ml) and dried. Crude phosphane was passed through a silica pad (Silica gel 70-230 mesh, eluent dichloromethane), dried *in vacuo* to afford solid precipitate.

(***ii***)**.** Excess of hydrogen peroxide (9.8 M, 30% aqueous solution, 10.00 eq.) for **1–3** or methyl iodide (10.00 eq.) for **4** was added to a degassed solution of the diphosphane in dichloromethane (10 ml). The resulting reaction mixture was stirred under N_2 atmosphere at room temperature for 1 h, then evaporated and the solid residue was purified by column chromatography (Silica gel 70-230 mesh, eluent dichloromethane-methanol, 99:1 → 96:4 for **1–3** and 99:1 → 92:8 for **4** v/v mixture), dried *in vacuo* to afford **1**–**4** as a white solid material.

(4-(*N*,*N*-diphenylamino)phenyl))(4-(*P*,*P*-diphenylphosphoryl)phenyl)phenylphosphane oxide, (**1**). (***i***). The parent diphosphane was prepared from **a1** (0.52 g, 1.02 mmol), *n*-BuLi (0.45 ml, 1.12 mmol) and PPh_2Cl (0.24 g, 1.07 mmol) in THF (30 ml). ^{31}P{^{1}H} NMR ($CDCl_3$; 298 K; δ): –6.2 (d, J_{PP} = 2.2 Hz, 1P), –7.4 (d, J_{PP} = 2.2 Hz, 1P).

(***ii***)**.** Oxidation of the diphosphane by H_2O_2 (1.04 ml, 10.20 mmol) gave **1** as a white amorphous powder (0.27 g, 42%). ^{1}H NMR (CD_2Cl_2, 298 K; *δ*): 7.74–7.83 (m, 4H, P-$C_6\underline{H_4}$-P), 7.66–7.73 (m, 6H, P-*o*-C_6H_5), 7.57–7.63 (m, 3H, P-*p*-C_6H_5), 7.49–7.53 (m, 6H, P-*m*-C_6H_5), 7.43 (dd, *J* = 11.4, 8.8 Hz, 2H, P-$C_6\underline{H_4}$-N), 7.32–7.36 (m, 4H, N-*o*-C_6H_5), 7.14–7.18 (m, 6H, N-*m*-C_6H_5 + N-*p*-C_6H_5), 7.00 (dd, *J* = 8.8, 2.3 Hz, 2H, P-$C_6\underline{H_4}$-N). ^{13}C{^{1}H} NMR (CD_2Cl_2; 298 K; δ): 151.9 (d, *J* = 2.6 Hz), 147.1, 138.4 (d, *J* =91.7 Hz), 137.5 (d, *J* = 92.4 Hz), 133.6 (d, *J* = 11.0 Hz), 132.2–132.7 (m), 130.2, 129.0–129.2 (m), 126.5, 125.1, 123.7, 122.5, 120.6 (d, *J* = 12.8 Hz). ^{31}P{^{1}H} NMR (CD_2Cl_2, 298 K; *δ*): 27.5

(d, J_{PP} = 3.6 Hz, 1P), 27.2 (d, J_{PP} = 3.6 Hz, 1P). Anal. Calcd for $C_{42}H_{33}NO_2P_2$: C, 78.13; H, 5.15; N, 2.17. Found: C, 77.97; H, 5.20; N, 2.14.

(4′-(*N*,*N*-diphenylamino)-[1,1′-biphenyl]-4-yl)(4-(*P*,*P*-diphenylphosphoryl)phenyl)phenyl phosphane oxide (2). **(*i*)**. The parent diphosphane was prepared from **a2** (1.08 g, 1.85 mmol), *n*-BuLi (0.81 ml, 2.03 mmol) and PPh_2Cl (0.43 g, 1.94 mmol) in THF (35 ml). $^{31}P\{^1H\}$ NMR ($CDCl_3$; 298 K; δ): –6.2 (d, J_{PP} = 2.2 Hz, 1P), –7.0 (d, J_{PP} = 2.2 Hz, 1P).

(*ii*). Oxidation of the diphosphane by H_2O_2 (1.90 ml, 18.50 mmol) gave **2** as white amorphous powder (0.51 g, 38%). ^1H NMR (CD_2Cl_2, 298 K; *δ*): 7.76–7.84 (m, 4H, P-C_6<u>H_4</u>-P), 7.59–7.75 (m, 13H, P-*p,o*-C_6H_5 + <u>P-C_6H_4</u>-C_6H_4-N), 7.49–7.55 (m, 8H, P-C_6H_4-C_6H_4-N + P-*m*-C_6H_5), 7.32 (dd, *J* = 8.4, 7.4 Hz, N-*m*-C_6H_5, 4H), 7.09–7.13 (m, 6H, N-*o*-C_6H_5 + P-C_6H_4-C_6<u>H_4-N</u>), 7.06 (t, *J* = 7.4 Hz, N-*p*-C_6H_5, 2H). $^{13}C\{^1H\}$ NMR (CD_2Cl_2; 298 K; δ): 148.8, 148.0, 144.9, 138.3 (d, *J* =13.9 Hz), 137.3 (d, *J* = 16.1 Hz), 133.5, 133.3, 133.0, 132.2–132.7 (m), 131.0, 129.9, 130.2, 129.2 (d, *J* =11.8 Hz), 128.5, 127.1 (d, *J* =12.1 Hz), 125.4, 123.9 (d, *J* =19.9 Hz). $^{31}P\{^1H\}$ NMR (CD_2Cl_2, 298 K; *δ*): 27.4 (d, J_{PP} = 3.5 Hz, 1P), 27.3 (d, J_{PP} = 3.5 Hz, 1P). Anal. Calcd for $C_{48}H_{37}NO_2P_2$: C, 79.88; H, 5.17; N, 1.94. Found: C, 79.88; H, 5.34; N, 1.80.

(4′-(9*H*-carbazol-9-yl)-[1,1′-biphenyl]-4-yl)(4-(*P*,*P*-diphenylphosphoryl)phenyl)phenyl phosphane oxide (3). **(*i*)**. The parent diphosphane was prepared from **a3** (1.30 g, 2.23 mmol), *n*-BuLi (1.00 ml, 2.45 mmol) and PPh_2Cl (0.52 g, 2.34 mmol) in THF (50 ml).$^{31}P\{^1H\}$ NMR ($CDCl_3$; 298 K; δ): –6.2 (d, J_{PP} = 2.2 Hz, 1P), –7.1 (d, J_{PP} = 2.2 Hz, 1P).

(*ii*). Oxidation of the diphosphane by H_2O_2 (2.27 ml, 22.30 mmol) gave **3** as a white amorphous powder (0.60 g, 37%). ^1H NMR (CD_2Cl_2, 298 K; *δ*): 8.19 (d, J_{HH} = 8.0 Hz, 2H, Carb), 7.90 (d, J_{HH} = 8.6 Hz, 2H, Carb), 7.51–7.87 (m, 27H), 7.42–7.49 (m, 2H, Carb), 7.33 (dd, *J* = 8.0 Hz, 2H, Carb). $^{13}C\{^1H\}$ NMR (CD_2Cl_2; 298 K; δ): 148.8, 148.0, 144.9, 133.6, 133.5, 133.3, 133.0 (d, *J* =9.8 Hz), 132.3–133.0 (m), 130.0, 129.9, 129.2 (d, *J* = 11.8 Hz), 128.4, 127.1 (d, *J* = 12.2 Hz), 125.4, 123.9, 123.8. $^{31}P\{^1H\}$ NMR (CD_2Cl_2, 298 K; *δ*): 27.3 (d, J_{PP} = 3.6 Hz, 1P), 27.2 (d, J_{PP} = 3.6 Hz, 1P). Anal. Calcd for $C_{48}H_{35}NO_2P_2$: C, 80.10; H, 4.90; N, 1.95. Found: C, 79.80; H, 4.79; N, 1.75.

(4-(*N*,*N*-diphenylamino)phenyl)(methyl)(4-(methyldiphenylphosphonio)phenyl)(phenyl) phosphonium iodide (4). **(*i*)**. The parent diphosphane was prepared from **a1** (0.30 g, 0.92 mmol), *n*-BuLi (0.41 ml, 1.02 mmol) and PPh_2Cl (0.21 g, 0.97 mmol) in THF (20 ml). Spectral data identical to those from first step for **1**.

(***ii***). Alkylation of the diphosphane by MeI (0.57 ml, 9.20 mmol) gave **4** as a pale crystalline solid (0.15 g, 29%). ESI-MS (*m/z*): $[M]^{2+}$ 321.6248 (calcd 321.6273). ^{1}H NMR (CD_2Cl_2, 298 K; *δ*): 8.08–8.16 (m, 4H, P-$C_6\underline{H_4}$-P), 7.72–7.94 (m, 15H, P-*o,m,p*-C_6H_5), 7.55 (dd, J_{HH} = 12.6, 9.0 Hz, 2H, $\underline{P\text{-}C_6H_4}$-N), 7.42 (t, J_{HH} = 7.8 Hz, 4H, N-*o*-C_6H_5), 7.25–7.29 (m, 6H, N-*m*-C_6H_5 + N-*p*-C_6H_5), 7.09 (dd, J_{HH} = 9.0, 2.9 Hz, 2H, P-$C_6\underline{H_4\text{-}N}$), 3.27 (d, J_{PH} = 13.5 Hz, 3H, Me), 3.11 (d, J_{PH} = 13.5 Hz, 3H, Me). $^{13}C\{^{1}H\}$ NMR (CD_2Cl_2; 298 K; δ): 154.7, 145.6, 136.1, 135.4–135.8 (m), 134.5 (d, *J* = 11.0 Hz), 134.2 (d, *J* = 11.1 Hz), 131.2 (d, *J* = 13.2 Hz), 130.6, 129.0 (d, *J* = 319.1 Hz), 126.8, 119.8, 119.6 (d, *J* = 14.0 Hz), 118.8, 117.9, 104.3, 103.5, 12.2, 11.6. $^{31}P\{^{1}H\}$ NMR (CD_2Cl_2, 298 K; *δ*): 23.0 (d, J_{PP} = 5.0 Hz, 1P), 21.3 (d, J_{PP} = 5.0 Hz, 1P). Anal. Calcd for $C_{44}H_{39}NI_2P_2$: C, 58.88; H, 4.38; N, 1.56. Found: C, 58.85; H, 4.50; N, 1.44.

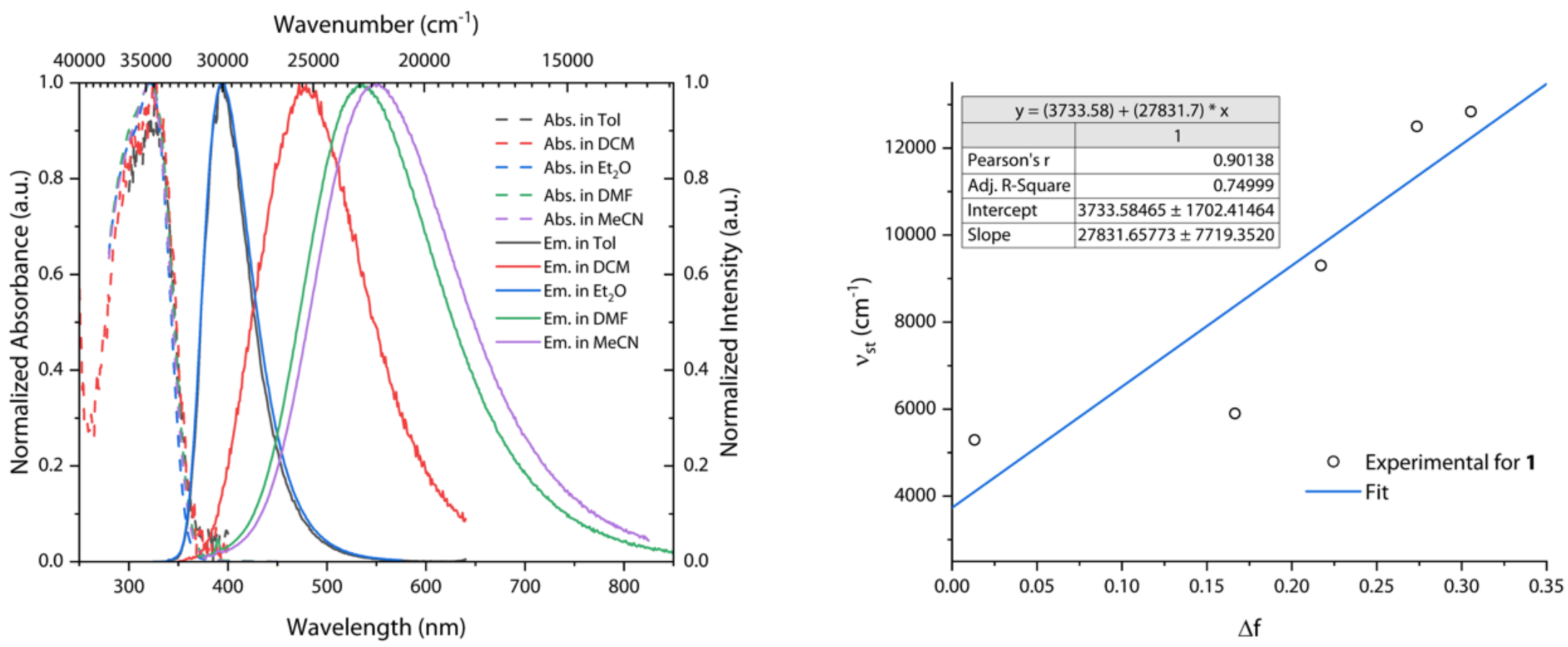

Figure S1. Normalized absorbance and emission of **1** in different solvents (left) and corresponding Lippert-Mataga plot (right).

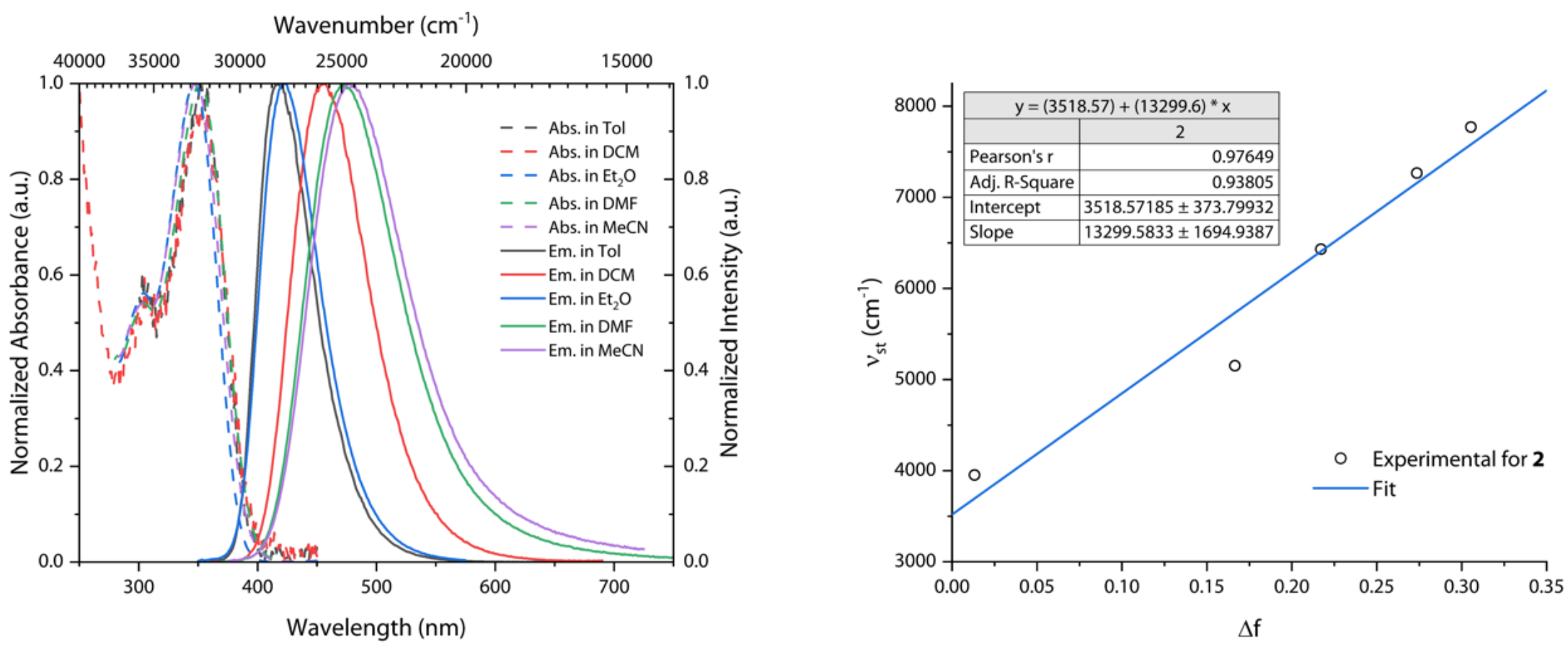

Figure S2. Normalized absorbance and emission of **2** in different solvents (left) and corresponding Lippert-Mataga plot (right).

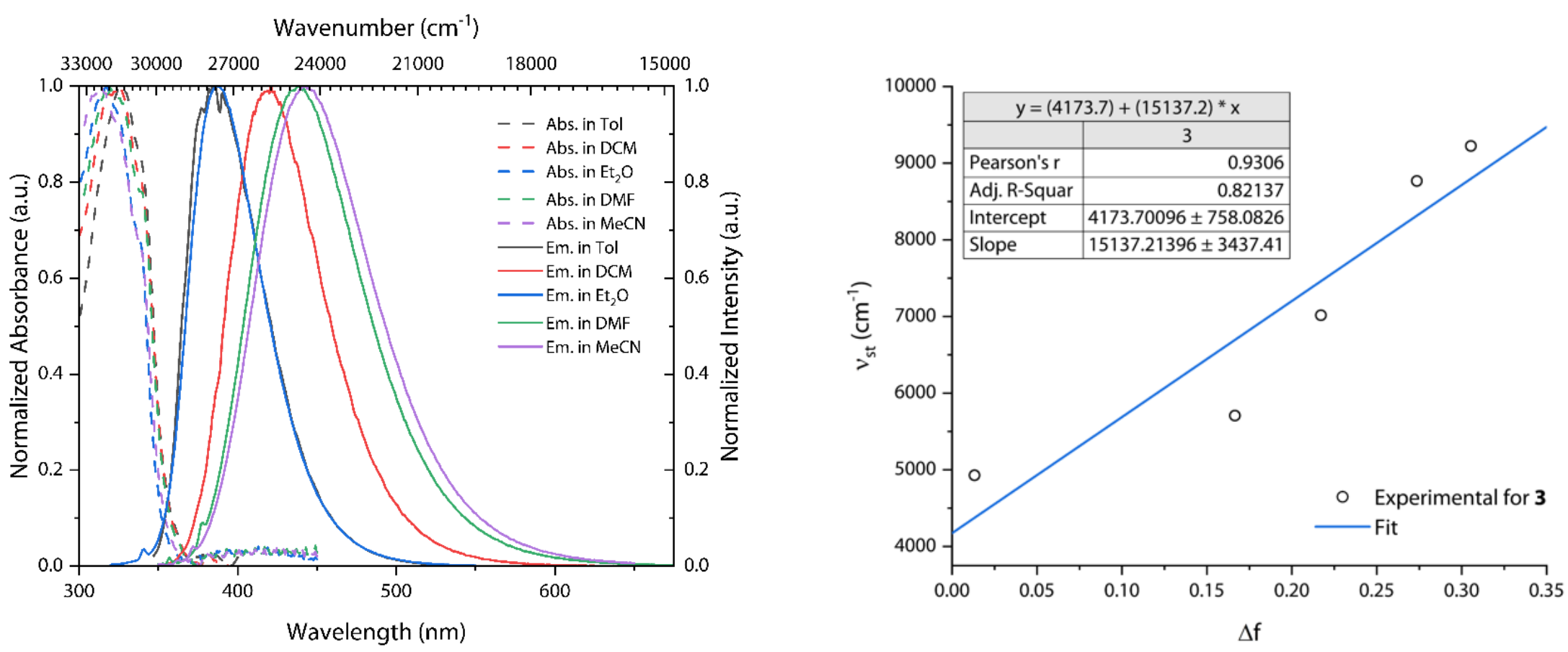

Figure S3. Normalized absorbance and emission of **3** in different solvents (left) and corresponding Lippert-Mataga plot (right).

Table S1. Photophysical characteristics of **1–3** in different solvents.

	Toluene	Et_2O	DCM	DMF	MeCN
			**1**		
λ_{abs} [nm]	326	321	328	321	321
λ_{em} [nm]	394	396	472	536	546
ν_{St} [cm^{-1}]	5294	5900	9301	12496	12838
CIE[a]	0.16, 0.04	0.16, 0.04	0.21, 0.29	0.35, 0.46	0.38, 0.47
			**2**		
λ_{abs} [nm]	303, 358	304, 348	305, 352	352	303, 348
λ_{em} [nm]	417	424	455	473	477
ν_{St} [cm^{-1}]	3952	5151	6431	7267	7771
CIE[a]	0.16, 0.04	0.16, 0.04	0.15, 0.14	0.18, 0.26	0.20, 0.29
			**3**		
λ_{abs} [nm]	325	317	325	317	314
λ_{em} [nm]	387	387	421	439	442
ν_{St} [cm^{-1}]	4929	5706	7016	8767	9223
CIE[a]	0.16, 0.02	0.16, 0.02	0.16, 0.05	0.16, 0.11	0.16, 0.12

[a](x,y), calculated from a steady-state emission spectra

1

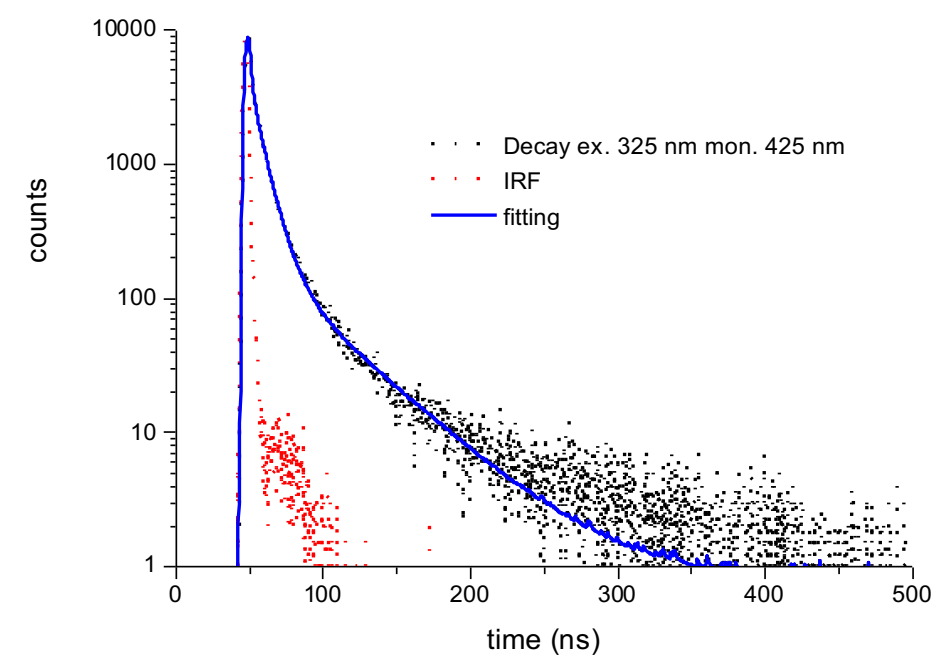

Lifetime(ns)	*Pre-exp factor*
1.11	*0.81*
8.16	*0.18*
44.22	*0.01*

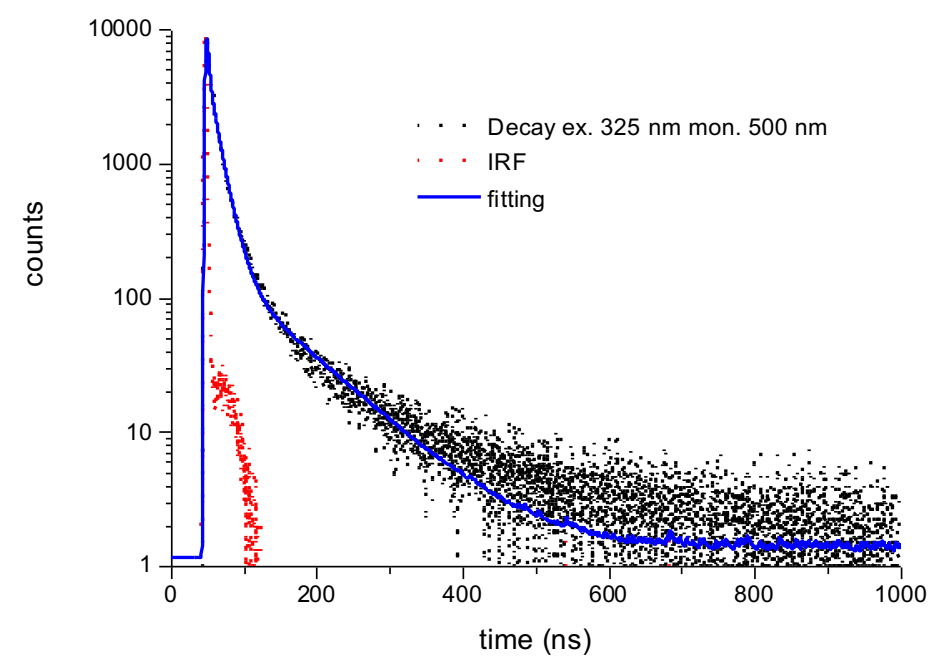

Lifetime(ns)	*Pre-exp factor*
1.83	*0.67*
12.74	*0.32*
87.8	*0.01*

2

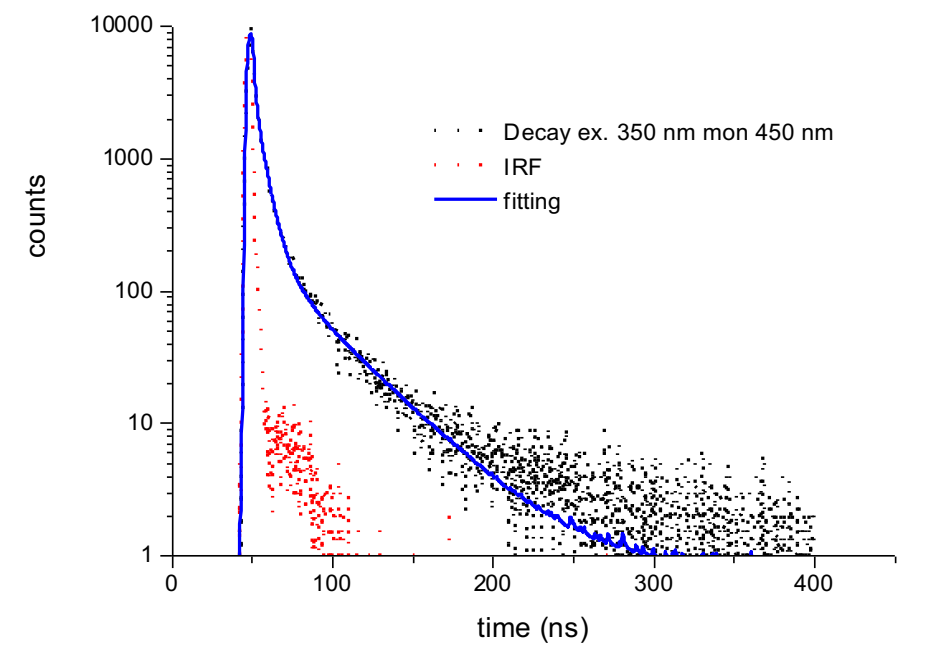

Lifetime(ns)	*Pre-exp factor*
0.99	*0.87*
5.89	*0.13*
36.73	*0.01*

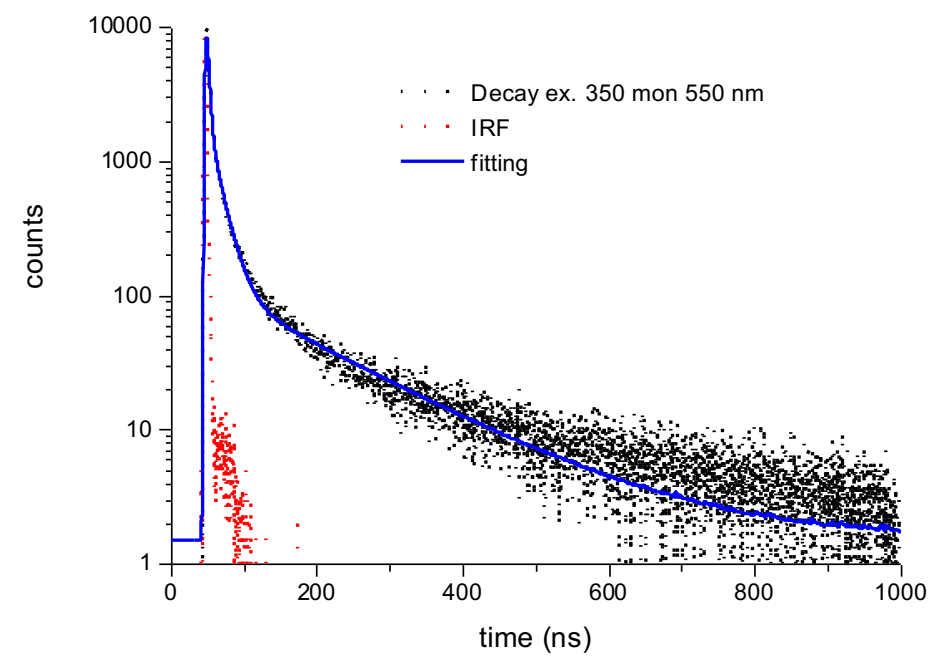

Lifetime(ns)	*Pre-exp factor*
2.81	*0.86*
15.58	*0.13*
150.68	*0.01*

Figure S4. Time-resolved emission spectra of solids **1–2** monitored at different wavelengths.

3

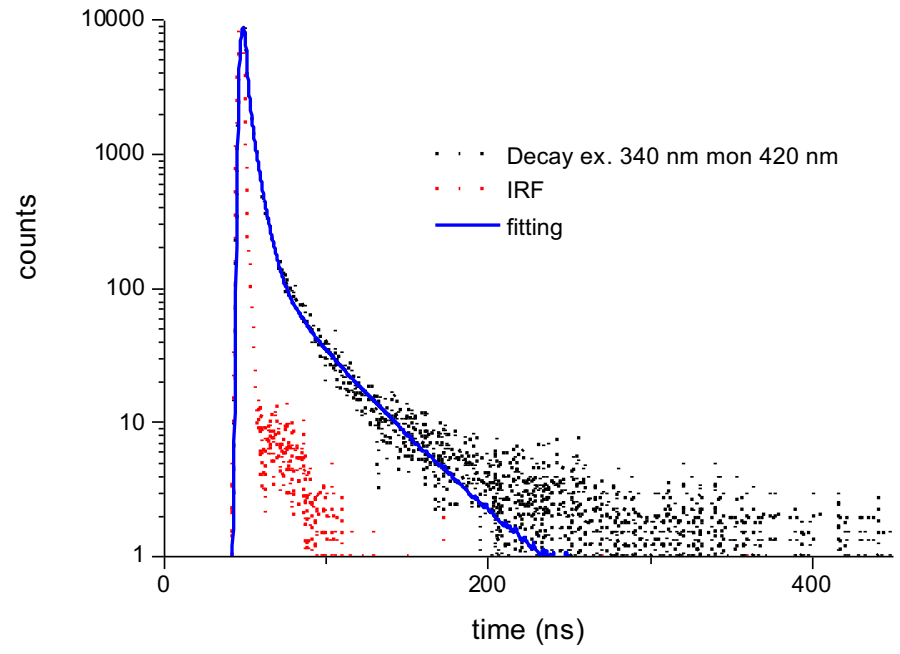

Lifetime(ns)	*Pre-exp factor*
1.07	*0.84*
5.35	*0.15*
35.15	*0.01*

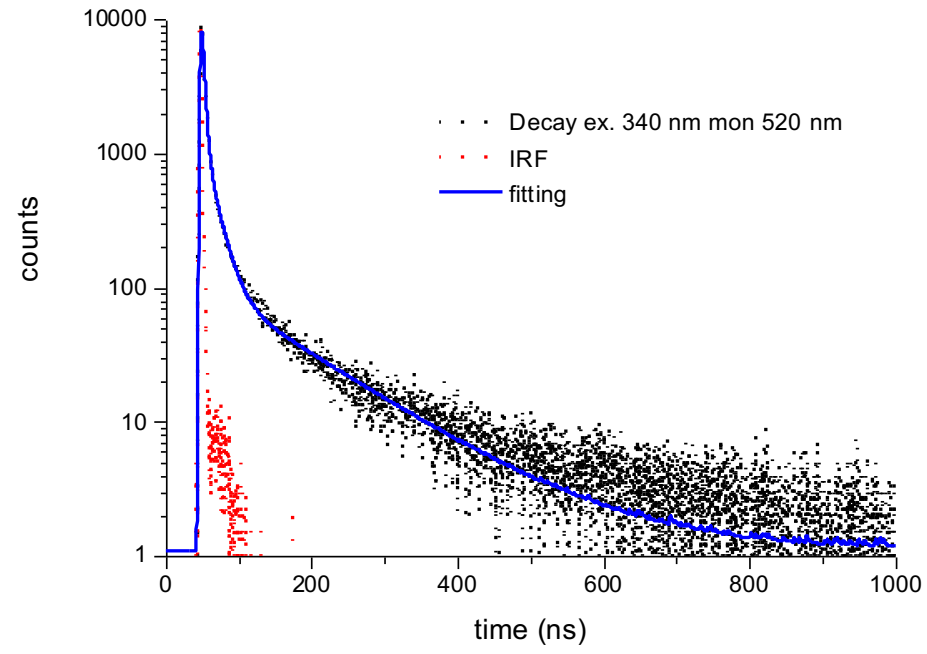

Lifetime(ns)	*Pre-exp factor*
3.14	*0.89*
15.16	*0.09*
124.35	*0.02*

Figure S5. Time-resolved emission spectra of solid **3** monitored at different wavelengths.

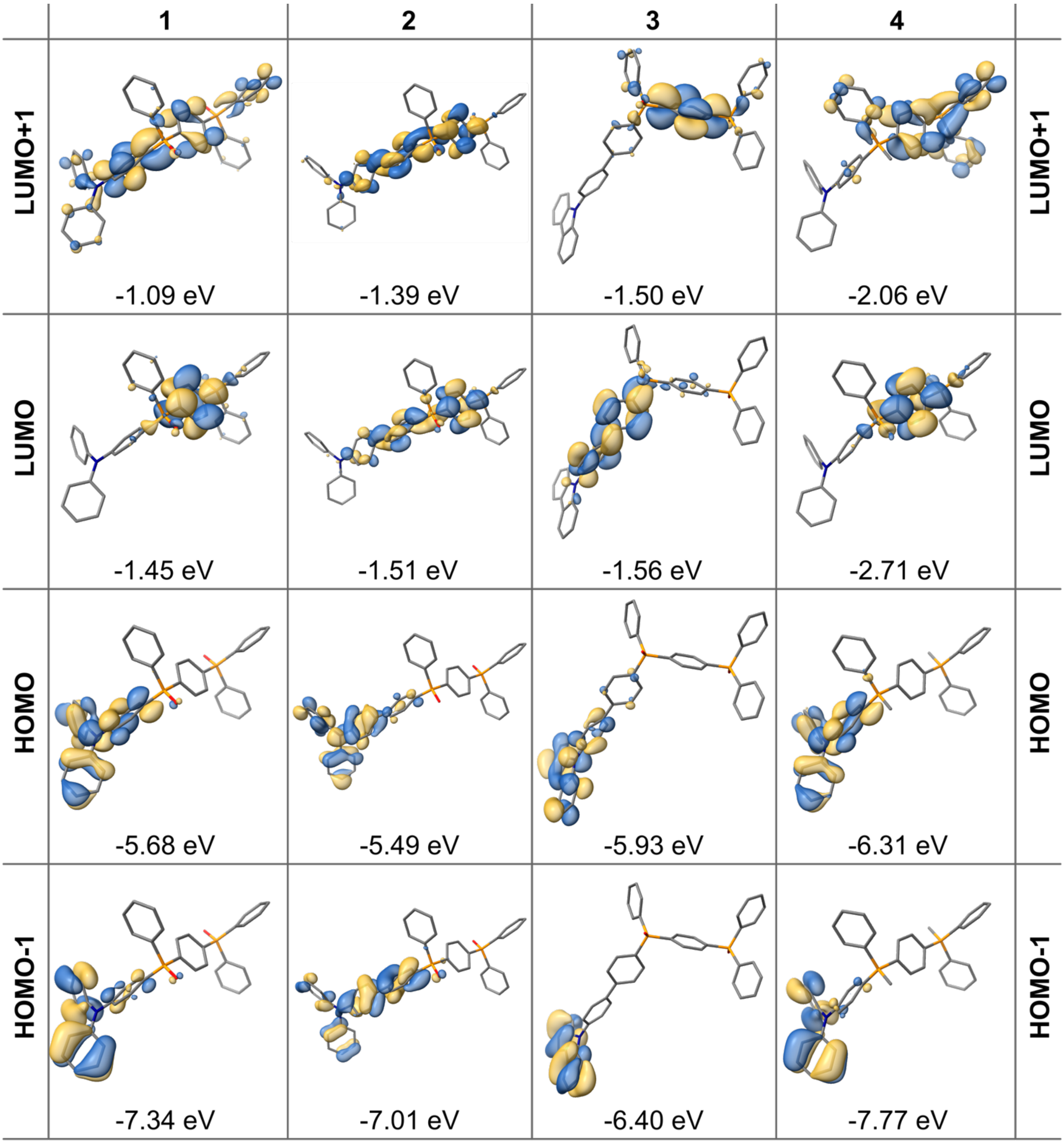

Figure S6. Selected molecular orbitals of **1**-**4**.

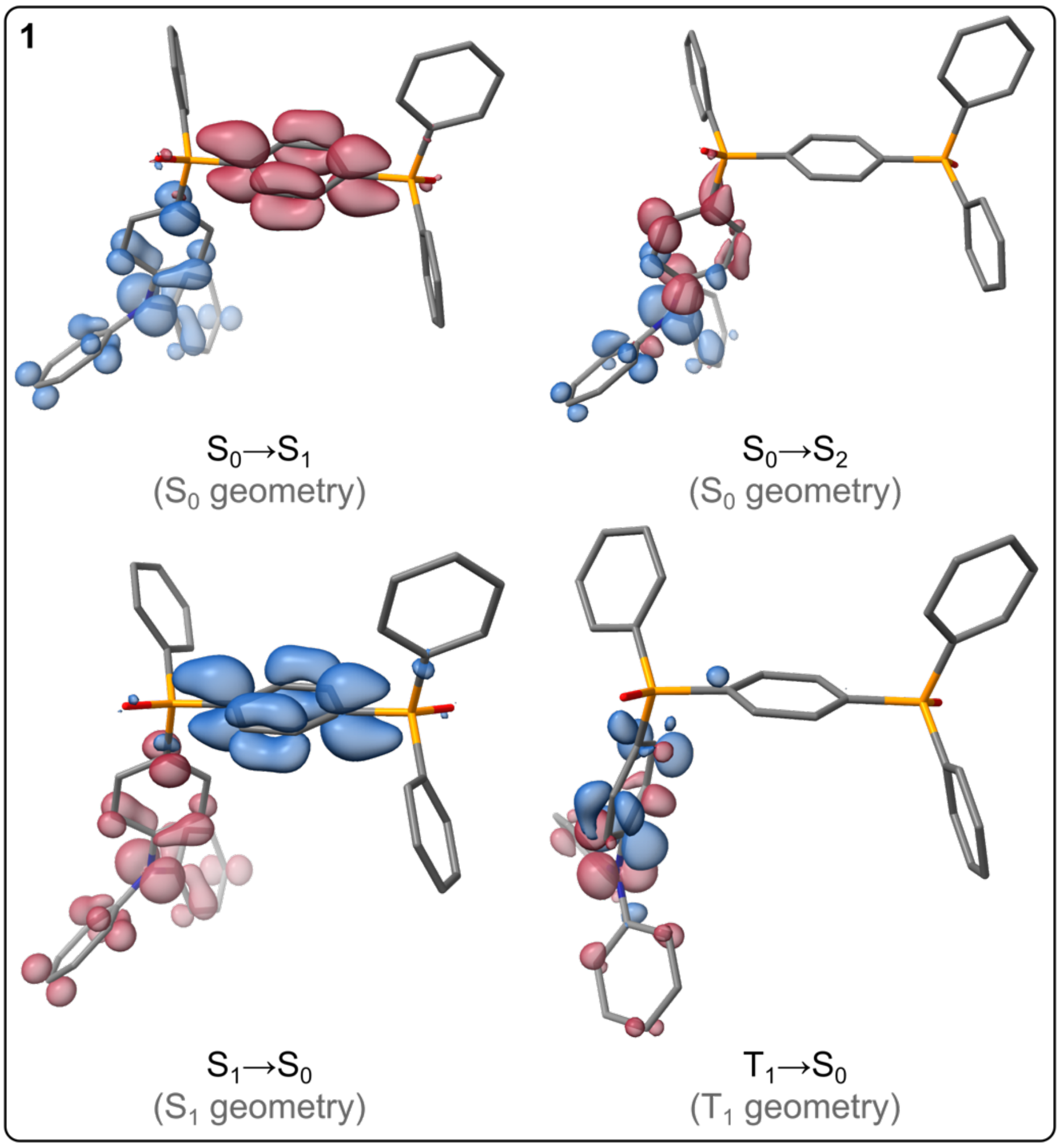

Figure S7. Selected difference densities calculated for **1** at S_0, S_1, and T_1 geometries. Blue: loss of electron density (blue), red: gain.

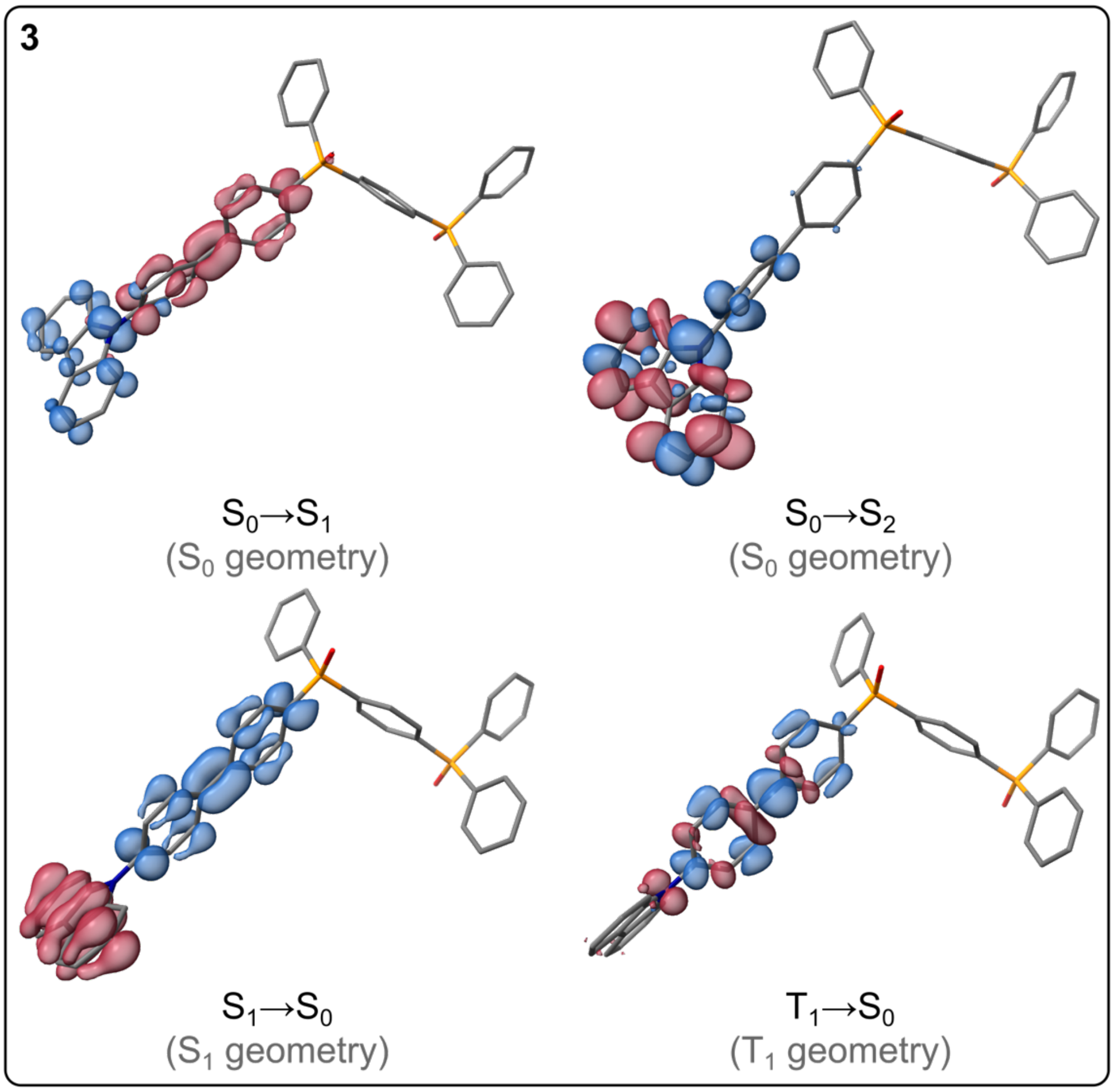

Figure S8. Selected difference densities calculated for **3** at S_0, S_1, and T_1 geometries. Blue: loss of electron density (blue), red: gain.

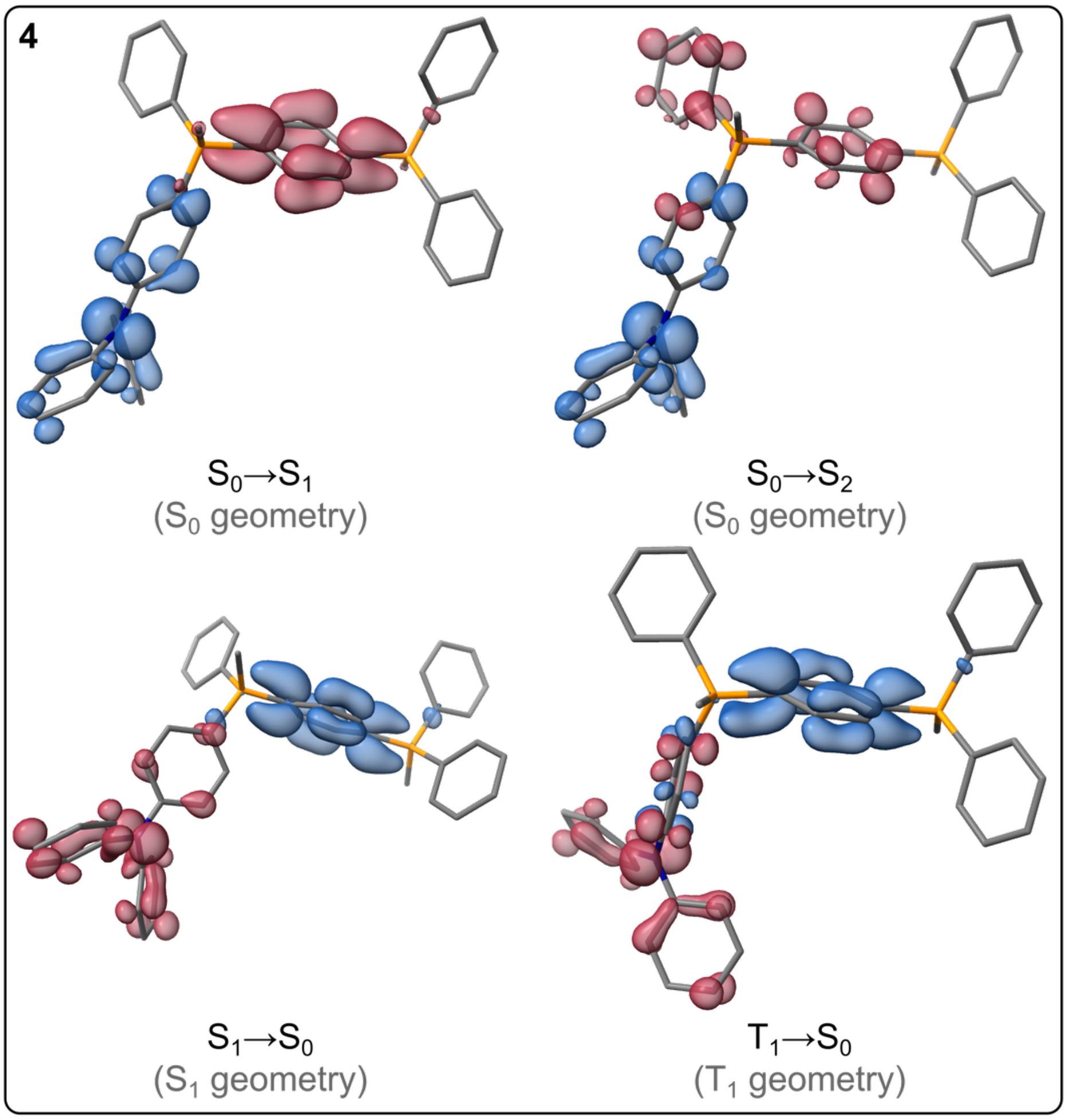

Figure S9. Selected difference densities calculated for **4** at S_0, S_1, and T_1 geometries. Blue: loss of electron density (blue), red: gain.

Table S2. Selected singlet and triplet excitations of **1** in the ground state geometry.

Excited state no.	Singlets				Triplets	
	E [eV]	λ [nm]	f×10³	Key orbital pairs [a]	E [eV]	λ [nm]
1	3.81	325.7	12.4	H→L (98%)	3.18	389.9
2	3.94	314.3	651.1	H→L+1 (80%)<br>H→L+2 (7%)<br>H→L+3 (6%)	3.48	356.3
3	4.01	309.0	73.1	H→L+2 (27%)<br>H→L+4 (30%)<br>H→L+5 (16%)<br>H→L+6 (18%)	3.62	342.3
4	4.28	289.6	256.0	H→L+3 (8%)<br>H→L+5 (6%)<br>H→L+6 (6%)<br>H→L+7 (75%)	3.80	326.3
5	4.31	287.9	35.1	H→L+1 (4%)<br>H→L+3 (42%)<br>H→L+4 (7%)<br>H→L+5 (25%)<br>H→L+7 (17%)	3.94	314.6
6	4.39	282.3	13.7	H→L+1 (11%)<br>H→L+2 (45%)<br>H→L+6 (28%)<br>H→L+8 (8%)	4.07	305.0
7	4.48	276.6	3.5	H→L+2 (11%)<br>H→L+3 (20%)<br>H→L+4 (57%)<br>H→L+6 (7%)	4.07	304.5
8	4.56	271.6	11.0	H→L+2 (8%)<br>H→L+3 (17%)<br>H→L+5 (46%)<br>H→L+6 (13%)<br>H→L+8 (8%)<br>H→L+11 (5%)	4.08	304.1
9	4.71	263.4	24.7	H→L+5 (5%)<br>H→L+6 (17%)<br>H→L+7 (4%)<br>H→L+8 (30%)<br>H→L+9 (5%)<br>H→L+11 (32%)	4.08	304.1
10	4.74	261.8	37.5	H→L+12 (89%)	4.10	302.1

[a] H = HOMO, L = LUMO.

Table S3. Selected singlet and triplet excitations of **2** in the ground state geometry.

Excited state no.	Singlets				Triplets	
	E [eV]	λ [nm]	f×10^3	Key orbital pairs [a]	E [eV]	λ [nm]
1	3.48	356.5	900.0	H→L (75%)<br>H→L+1 (21%)	2.89	428.4
2	3.81	325.7	28.1	H→L (23%)<br>H→L+1 (76%)	3.41	363.8
3	3.95	313.5	23.8	H→L+5 (4%)<br>H→L+6 (84%)<br>H→L+7 (4%)	3.55	349.5
4	4.17	297.6	285.5	H→L+7 (15%)<br>H→L+8 (77%)	3.76	329.8
5	4.22	294.1	20.7	H→L+2 (43%)<br>H→L+3 (28%)<br>H→L+4 (12%)<br>H→L+8 (7%)	3.80	326.0
6	4.30	288.3	2.9	H→L+2 (28%)<br>H→L+3 (61%)<br>H→L+5 (9%)	3.94	315.0
7	4.36	284.6	3.5	H→L+2 (21%)<br>H→L+4 (61%)<br>H→L+5 (6%)<br>H→L+7 (4%)	4.00	310.0
8	4.44	279.0	7.5	H→L+2 (4%)<br>H→L+4 (23%)<br>H→L+5 (45%)<br>H→L+7 (12%)<br>H→L+9 (5%)	4.07	304.9
9	4.51	274.8	17.7	H→L+5 (31%)<br>H→L+6 (6%)<br>H→L+7 (22%)<br>H→L+8 (5%)<br>H→L+9 (14%)<br>H→L+13 (10%)	4.07	304.3
10	4.60	269.4	21.6	H→L+7 (5%)<br>H→L+11 (7%)<br>H→L+12 (23%)<br>H→L+13 (30%)<br>H→L+14 (23%)	4.08	304.0

[a] H = HOMO, L = LUMO.

Table S4. Selected singlet and triplet excitations of **3** in the ground state geometry.

Excited state no.	Singlets				Triplets	
	E [eV]	λ [nm]	f×10^3	Key orbital pairs [a]	E [eV]	λ [nm]
1	3.80	326.6	533.5	H→L (97 %)	3.26	379.8
2	4.15	298.5	84.4	H→L+2 (91 %)	3.46	358.0
3	4.24	292.5	0.1	H→L+1 (98 %)	3.49	355.5
4	4.37	283.8	0.2	H-1→L (97 %)	3.91	317.5
5	4.52	274.2	2.7	H→L+5 (7 %)<br>H→L+6 (8 %)<br>H→L+7 (38 %)<br>H→L+8 (33 %)	3.94	314.4
6	4.72	262.7	136.6	H-1→L+2 (63 %)<br>H→L+12 (25 %)	4.06	305.1
7	4.73	262.3	7.1	H→L+3 (53 %)<br>H→L+4 (8 %)<br>H→L+5 (5 %)<br>H→L+6 (7 %)<br>H→L+8 (12 %)	4.07	304.5
8	4.74	261.6	0.4	H→L+3 (32 %)<br>H→L+4 (8 %)<br>H→L+5 (47 %)<br>H→L+8 (5 %)	4.08	303.8
9	4.77	259.8	0.0	H-1→L+1 (100 %)	4.14	299.5
10	4.82	257.0	0.0	H→L+3 (5 %)<br>H→L+4 (64 %)<br>H→L+5 (29 %)	4.19	296.2

[a] H = HOMO, L = LUMO.

Table S5. Selected singlet and triplet excitations of **4** in the ground state geometry.

Excited state no.	Singlets				Triplets	
	E [eV]	λ [nm]	f×10^3	Key orbital pairs [a]	E [eV]	λ [nm]
1	3.11	398.6	8.9	H→L (99 %)	3.03	409.1
2	3.80	326.3	79.3	H→L+1 (66 %)<br>H→L+2 (4 %)<br>H→L+3 (18 %)<br>H→L+4 (6 %)	3.11	398.0
3	3.85	322.1	704.3	H→L+2 (17 %)<br>H→L+3 (16 %)<br>H→L+4 (60 %)	3.52	352.1
4	4.01	309.4	91.9	H→L+1 (23 %)<br>H→L+2 (29 %)<br>H→L+3 (14 %)<br>H→L+4 (17 %)<br>H→L+5 (15 %)	3.70	335.5
5	4.09	303.1	1.6	H→L+2 (49 %)<br>H→L+3 (42 %)<br>H→L+5 (6 %)	3.91	317.0
6	4.14	299.2	17.6	H→L+1 (6 %)<br>H→L+5 (23 %)<br>H→L+6 (41 %)<br>H→L+7 (18 %)<br>H→L+8 (4 %)	3.95	313.7
7	4.26	291.1	15.6	H→L+3 (10 %)<br>H→L+4 (9 %)<br>H→L+5 (50 %)<br>H→L+6 (14 %)<br>H→L+7 (13 %)	3.98	311.7
8	4.52	274.4	234.1	H→L+8 (6 %)<br>H→L+10 (88 %)	4.06	305.7
9	4.64	267.5	4.2	H→L+6 (27 %)<br>H→L+7 (16 %)<br>H→L+8 (41 %)<br>H→L+10 (4 %)<br>H→L+11 (9 %)	4.06	305.3
10	4.70	263.9	1.3	H-1→L (96 %)	4.07	304.9

[a] H = HOMO, L = LUMO.

Table S6. Energies of selected excited states of **1-4** at S_1 geometry.

Excited state no.	**1** (eV)		**2** (eV)		**3** (eV)		**4** (eV)	
	Singlets	Triplets	Singlets	Triplets	Singlets	Triplets	Singlets	Triplets
1	2.95	2.89	2.85	2.34	3.18	2.55	2.11	2.10
2	3.67	3.08	3.70	3.23	3.91	3.37	3.30	2.82
3	3.84	3.28	3.80	3.32	4.02	3.44	3.56	3.30
4	4.03	3.45	4.01	3.44	4.09	3.48	3.64	3.32
5	4.15	3.46	4.08	3.59	4.21	3.86	3.77	3.39

Table S7. Energies of selected excited states of **1-4** at T_1 geometry.

Excited state no.	**1** (eV)		**2** (eV)		**3** (eV)		**4** (eV)	
	Singlets	Triplets	Singlets	Triplets	Singlets	Triplets	Singlets	Triplets
1	3.22	2.63	2.87	2.17	3.10	2.33	2.27	2.14
2	3.85	3.31	3.63	3.31	3.84	3.40	3.43	2.95
3	3.89	3.51	3.80	3.39	3.96	3.43	3.49	3.31
4	4.07	3.62	3.97	3.43	3.98	3.44	3.69	3.35
5	4.08	3.88	4.05	3.58	4.16	3.79	3.82	3.49

**Table S8**. SOCME^{TOTAL} values between T_{1-5} and S_{0-5} states of **1**-**3** at S_1 geometry.				
Root		SOCME^{TOTAL} [cm^{-1}]		
**T**	**S**	**1**	**2**	**3**
1	0	1.2	1.0	1.0
1	1	0.7	0.2	0.4
1	2	2.7	1.4	0.3
1	3	0.5	0.4	0.3
1	4	0.5	0.8	1.3
1	5	2.4	0.9	0.7
2	0	1.9	1.6	0.8
2	1	1.9	0.8	0.4
2	2	0.9	0.4	0.0
2	3	1.1	0.7	0.0
2	4	1.1	0.2	0.1
2	5	1.0	0.1	0.3
3	0	1.7	1.3	1.1
3	1	0.1	0.4	0.3
3	2	1.1	0.0	0.0
3	3	0.8	0.3	0.3
3	4	0.4	0.5	0.4
3	5	0.3	0.2	0.6
4	0	0.8	1.2	0.0
4	1	0.8	0.5	0.2
4	2	0.6	0.3	0.2
4	3	0.1	0.2	0.0
4	4	0.6	0.8	0.1
4	5	0.7	0.2	0.1
5	0	0.3	0.9	0.5
5	1	0.7	1.2	0.2
5	2	0.2	0.6	0.0
5	3	0.1	0.3	0.1
5	4	0.4	0.6	0.1
5	5	0.4	1.2	0.3

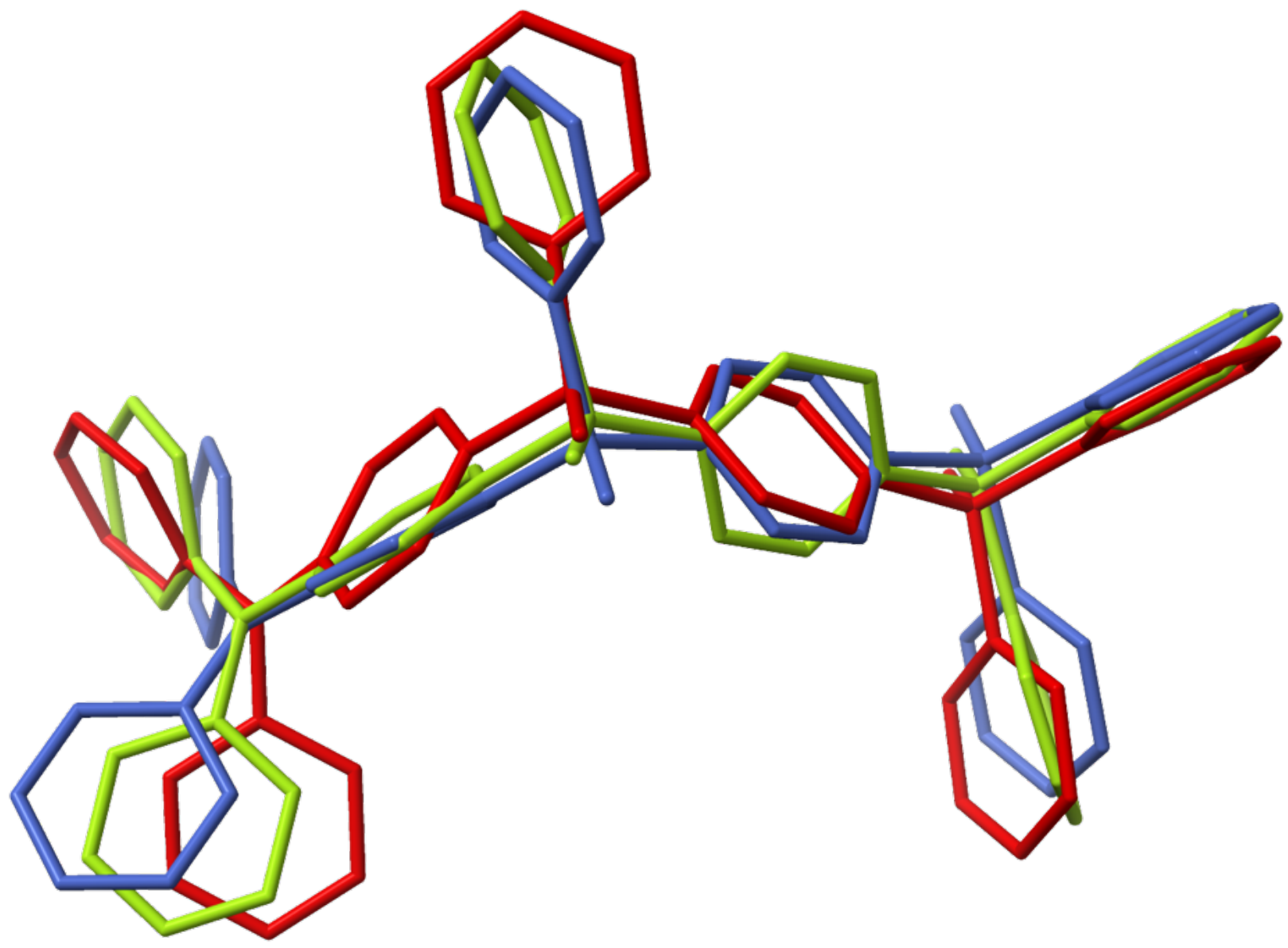

Figure S10. Superimposed coordinates of optimized S_0 (blue), S_1 (green) and T_1 (red) states of **1**.

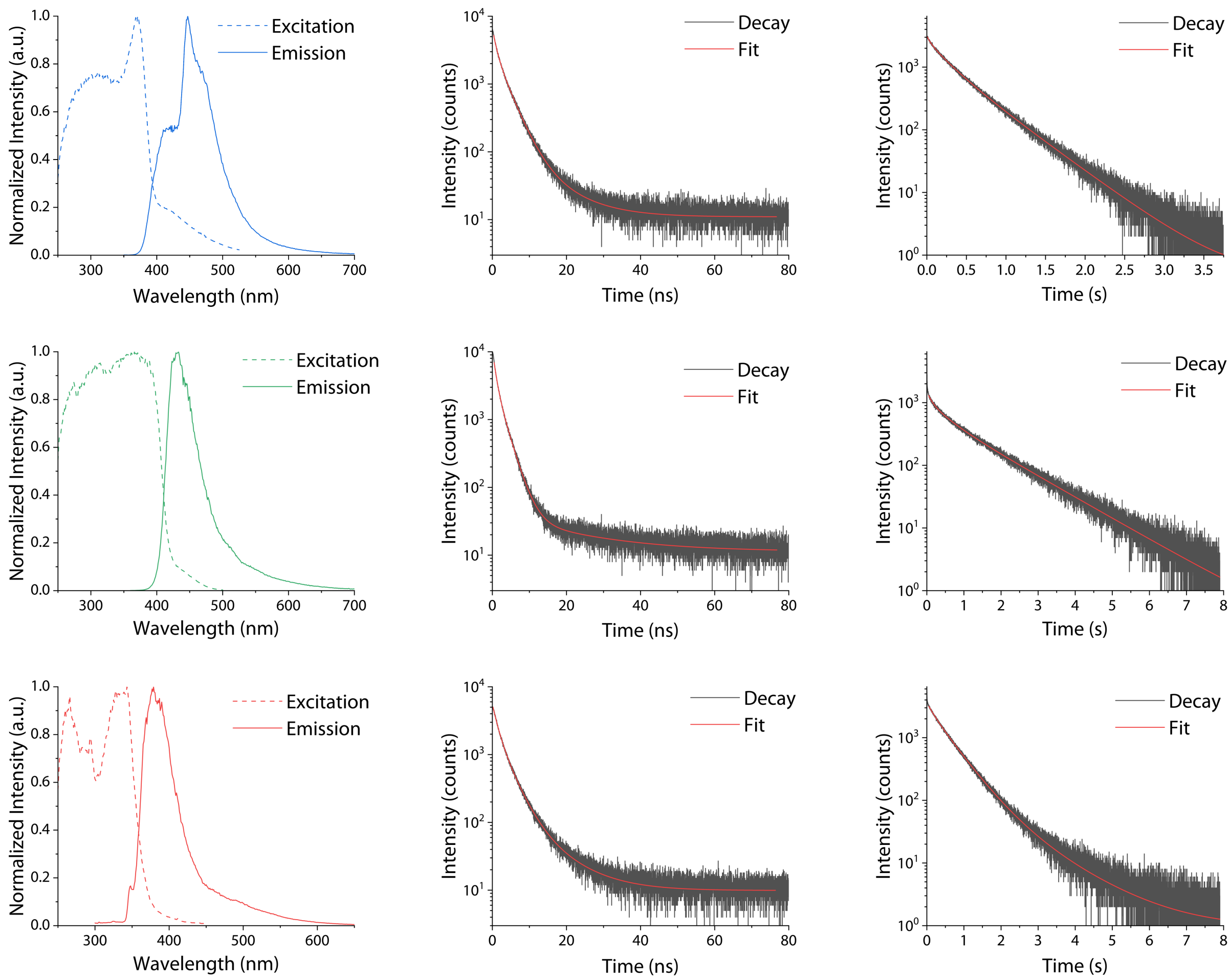

Figure S11. Photoluminescence (left), fluorescence lifetimes (middle), and phosphorescence lifetimes (right) of frozen DCM solutions (77 K) of **1** (upper line), **2** (middle line) and **3** (bottom line).

Table S9. Summary of the photophysical properties of frozen DCM solutions of **1**–**3**.

Compound	λ_{exc} [nm]	λ_{em} [nm]	τ_f (A_i) [ns]	$\tau_{f, av}$ [ns][a]	τ_p (A_i) [ms] [a]	$\tau_{p, av}$ (A_i) [ms] [a]
**1**	312, 370	421, 447	0.91 (41.36 %)	2.35	47.70 (15.38 %)	315.27
			3.09 (55.95 %)		221.16 (36.61 %)	
			9.10 (2.69 %)		472.75 (48.01 %)	
**2**	363	432	0.74 (50.58 %)	1.58	115.18 (20.93 %)	807.73
			2.30 (48.99 %)		443.94 (27.59 %)	
			19.21 (0.43 %)		1284.27 (51.48 %)	
**3**	266, 293, 336	348, 379	1.26 (50.77 %)	2.43	148.16 (12.37 %)	511.05
			3.21 (45.53 %)		512.64 (81.43 %)	
			9.00 (3.70 %)		1214.27 (6.20 %)	

[a] Amplitude-weighted average lifetimes, calculated from $\tau_{av}=\Sigma A_i \tau_i$, A_i=weight of the i-th exponent.

Table S10. Crystal data and structure refinement for **2**.

Identification code	**2**
CCDC number	2505612
Empirical formula	$C_{48}H_{37}NO_2P_2$
Formula weight	721.72
Temperature [K]	150.0
Crystal system	monoclinic
Space group	Cc
a [Å]<br>b [Å]<br>c [Å]<br>α [°]<br>β [°]<br>γ [°]	29.689(3)<br>13.7994(12)<br>11.1071(9)<br>90<br>109.551(2)<br>90
Volume [Å^3]	4288.1(6)
Z	4
ρ_{calc} [g/cm^3]	1.118
μ [mm^{-1}]	0.138
F(000)	1512.0
Crystal size [mm^3]	0.233 × 0.177 × 0.101
Radiation type	MoKα (λ = 0.71073)
2Θ range for data collection [°]	5.272 to 52
Index ranges	-36 ≤ h ≤ 34, -17 ≤ k ≤ 16, -6 ≤ l ≤ 13
Reflections collected	15355
Independent reflections	5561 [R_{int} = 0.0218, R_{sigma} = 0.0246]
Data/restraints/parameters	5561/2/478
Goodness-of-fit on F^2 [(a)]	1.031
Final R indexes [I>=2σ (I)] [(b)]	R_1 = 0.0302, wR_2 = 0.0780
Final R indexes [all data] [(b)]	R_1 = 0.0342, wR_2 = 0.0801
Largest diff. peak/hole [e/Å^{-3}]	0.27/-0.17
Flack parameter	0.51(4)

[(a)] GooF = S = $[[\sum w(F_o^2-F_c^2)^2]/(m-n)]^{1/2}$, where m = number of reflexes and n = number of parameters; [(b)] $R_1 = \sum||F_o|-|F_c||/\sum|F_o|$; $wR_2 = [\sum[w(F_o^2-F_c^2)^2/\sum[(wF_o^2)^2]]^{1/2}$; $w = 1/[\sigma^2(F_o^2)+(aP)^2+bP]$, where $P = (F_o^2+2F_c^2)$

Analytical chiral HPLC separation for **2:** The sample is dissolved in a mixture of ethanol and dichloromethane, injected on the chiral column, and detected with a UV detector at 254 nm. The flow-rate is 1 mL/min. Preparative separation for compound **2**: Sample preparation: About 40 mg of compound **2** are dissolved in 2.5 mL of a mixture of ethanol and hexane (1:1). Chromatographic conditions: Chiralpak IG (250 x 10 mm), hexane/ethanol/dichloromethane (10/40/50) as mobile phase, flow-rate = 5 mL/min, UV detection at 280 nm. Injections: 10 times 250 µL. First fraction: 18.6 mg of the first eluted enantiomer with ee > 99.5 %. Second fraction: 18.9 mg of the second eluted enantiomer with ee > 99.5 %.

Analytical chiral HPLC separation for **3.** The sample was dissolved in a mixture of ethanol and dichloromethane, injected on the chiral column, and detected with a UV detector at 254 nm. The flow rate was 1 mL/min. Preparative separation for compound **3**: Sample preparation: About 2 mg of compound **3** was dissolved in 0.5 mL of a mixture of ethanol and hexane (1:1). Chromatographic conditions: Chiralpak IG (250 x 10 mm), hexane/ethanol/dichloromethane (10/40/50) as mobile phase, flow-rate = 5 mL/min, UV detection at 280 nm. Injections: 2 times 250 µL. First fraction: 0.7 mg of the first eluted enantiomer with ee > 99.5 %. Second fraction: 0.7 mg of the second eluted enantiomer with ee > 99.5 %.

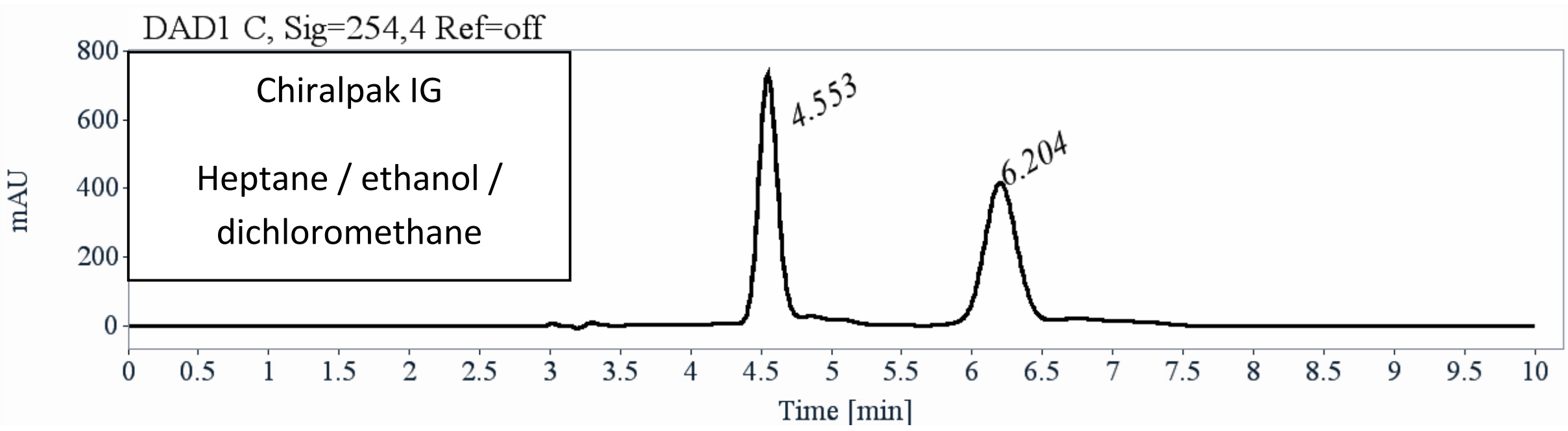

RT [min]	Area	Area%	Capacity Factor	Enantioselectivity	Resolution (USP)
4.55	6924	51.46	0.54		
6.20	6533	48.54	1.10	2.03	4.86
Sum	13457	100.00			

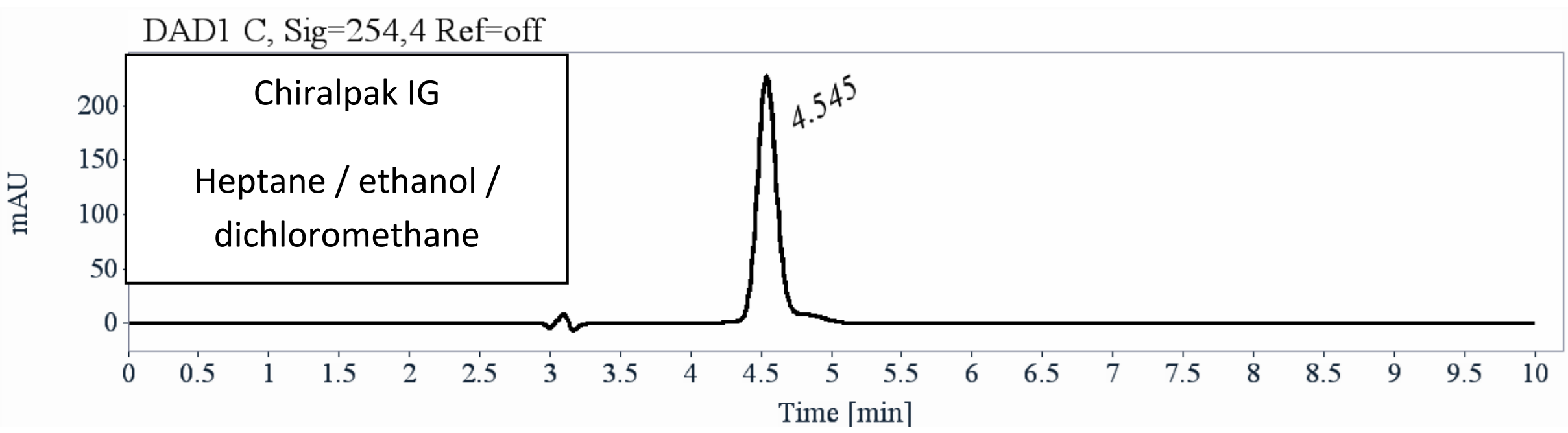

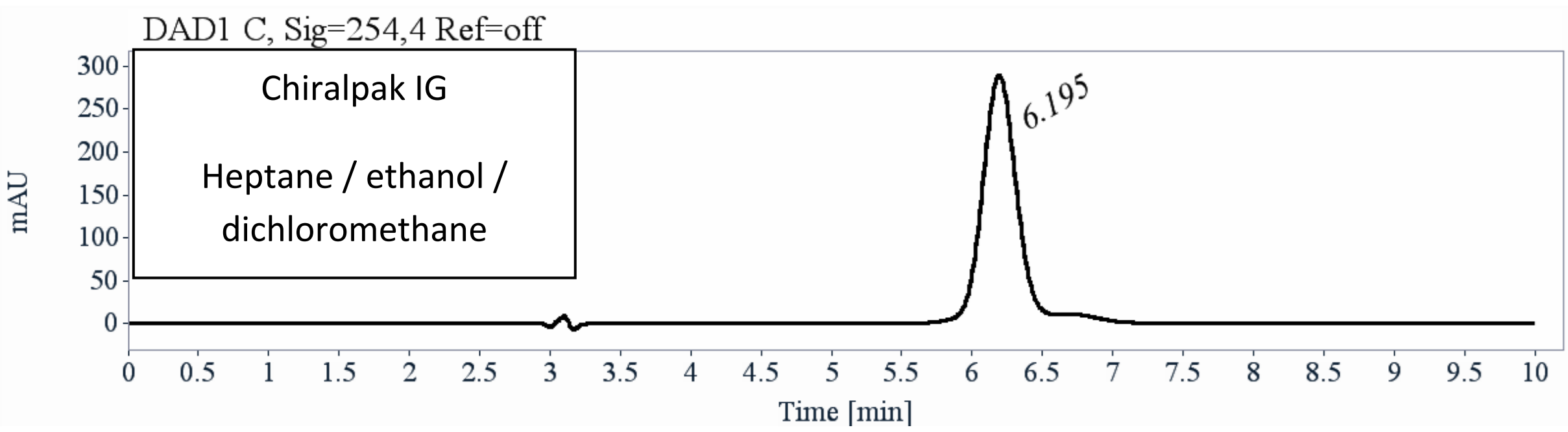

RT [min]	Area	Area%
4.54	2228	100.00
Sum	2228	100.00

RT [min]	Area	Area%
6.20	4629	100.00
Sum	4629	100.00

Figure S12. Separation details of the **2-*S*** and **2-*R*** enantiomers.

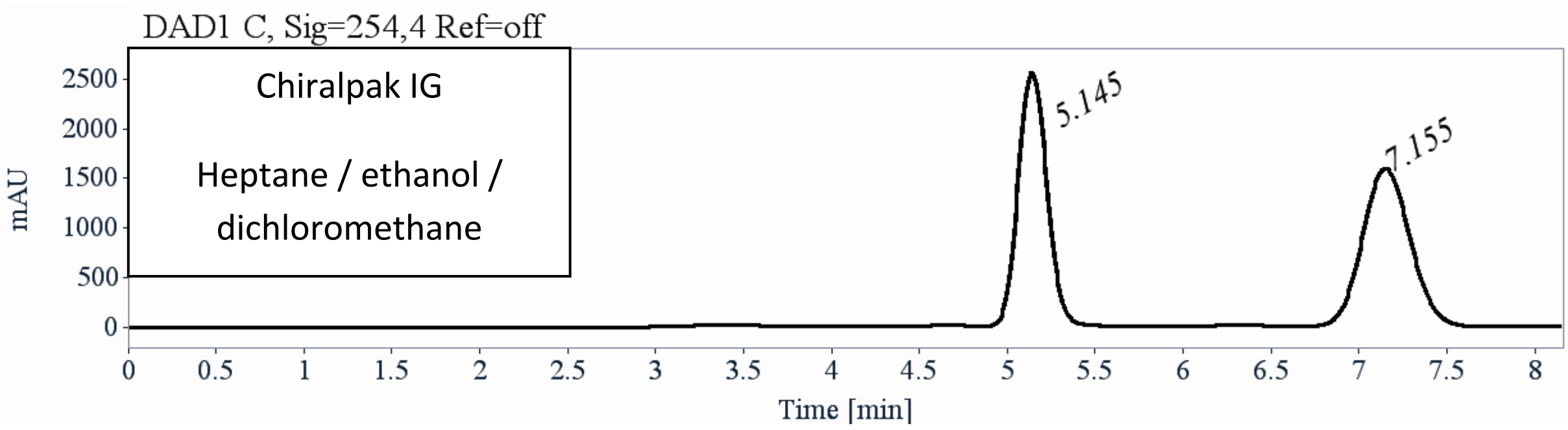

RT [min]	Area	Area%	Capacity Factor	Enantioselectivity	Resolution (USP)
5.15	29334	49.23	0.74		
7.16	30250	50.77	1.43	1.92	5.03
Sum	59584	100.00			

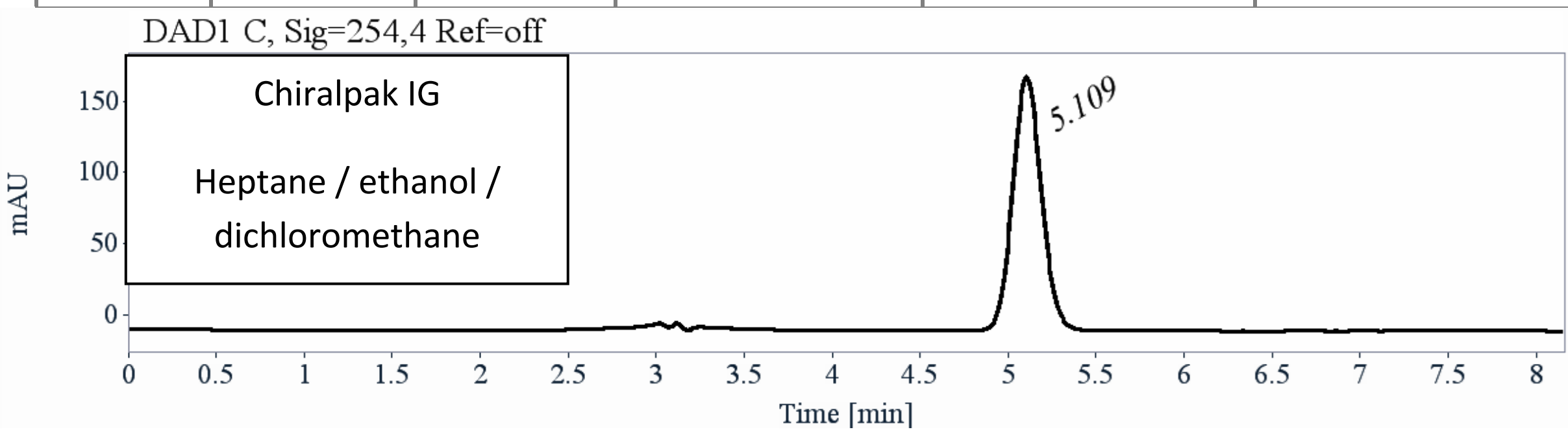

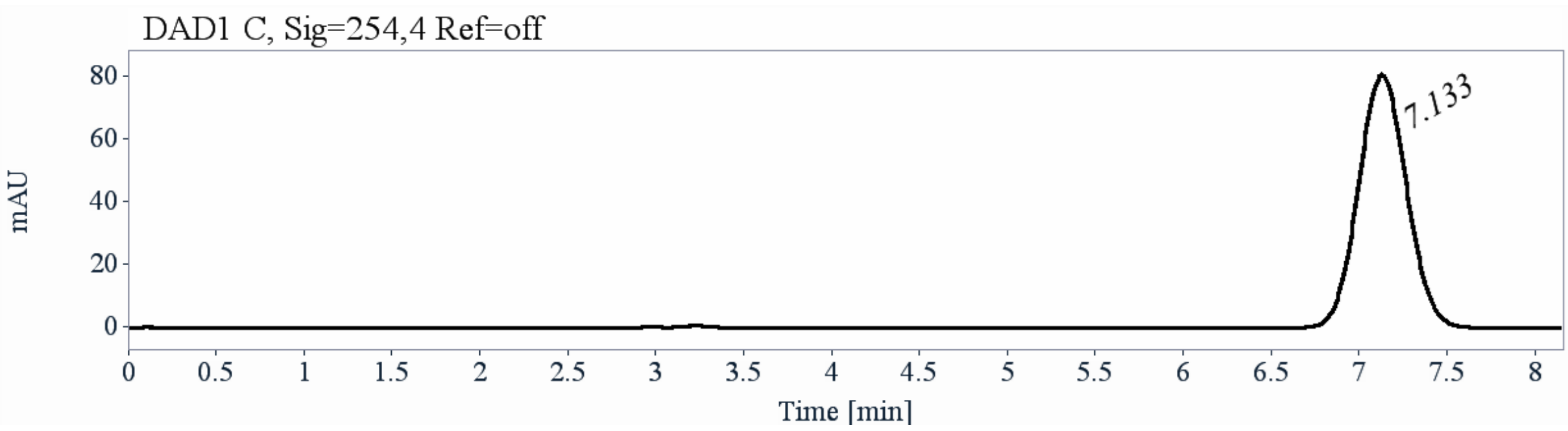

RT [min]	Area	Area%	RT [min]	Area	Area%
5.11	2036	100.00	7.13	1542	100.00
Sum	2036	100.00	Sum	1542	100.00

Figure S13. Separation details of the **3-*S*** and **3-*R*** enantiomers.

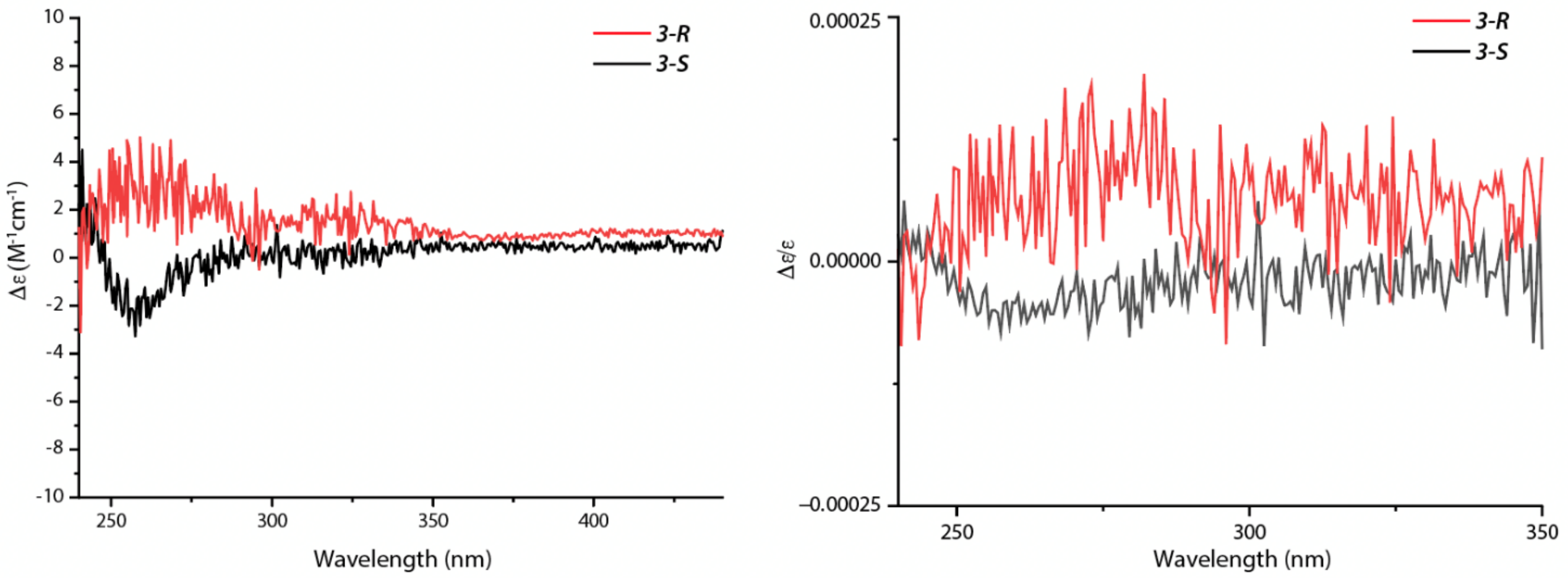

Figure S14. Electronic circular dichroism spectra of **3-*R*** (red) and **3-*S*** (black) enantiomers in DCM.

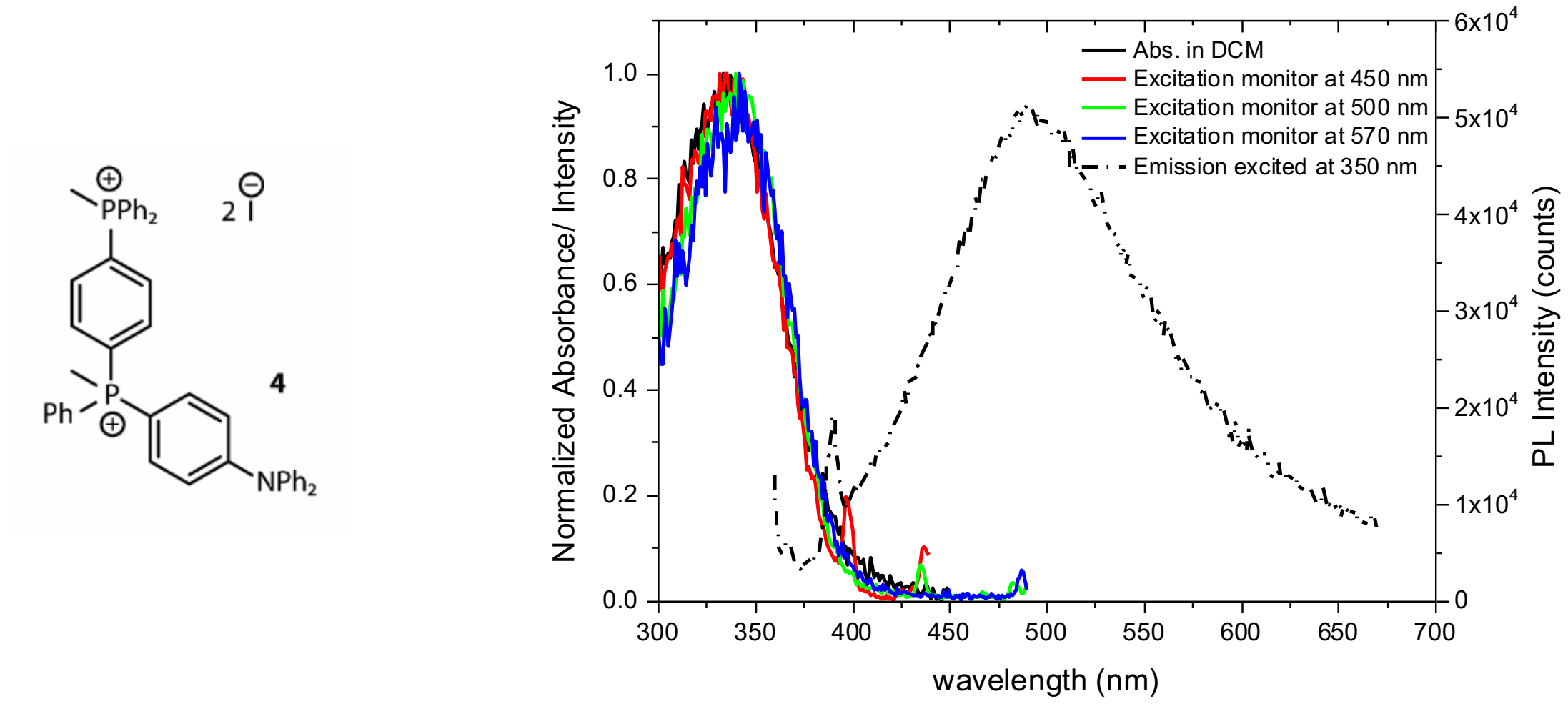

Figure S15. Structure of **4** and its normalized absorption, excitation, and emission spectra in DCM solution.

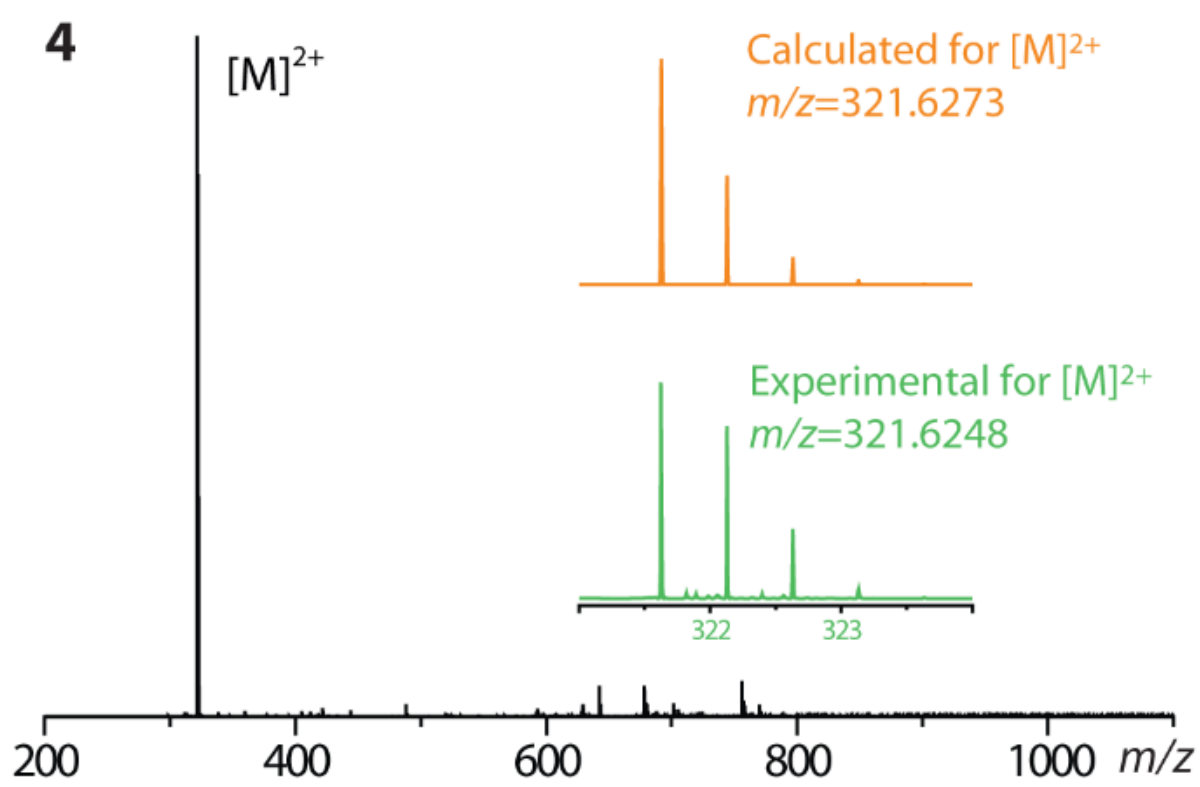

Figure S16. ESI-MS of compound **4** and the calculated isotopic distribution for molecular cations.

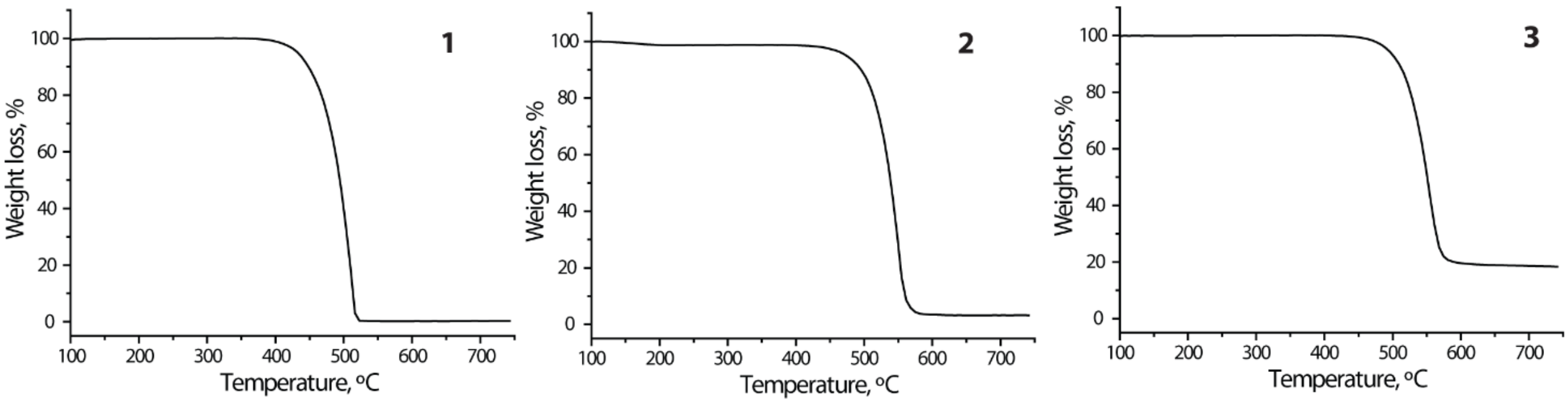

Figure S17. TGA curves of compounds **1–3**.

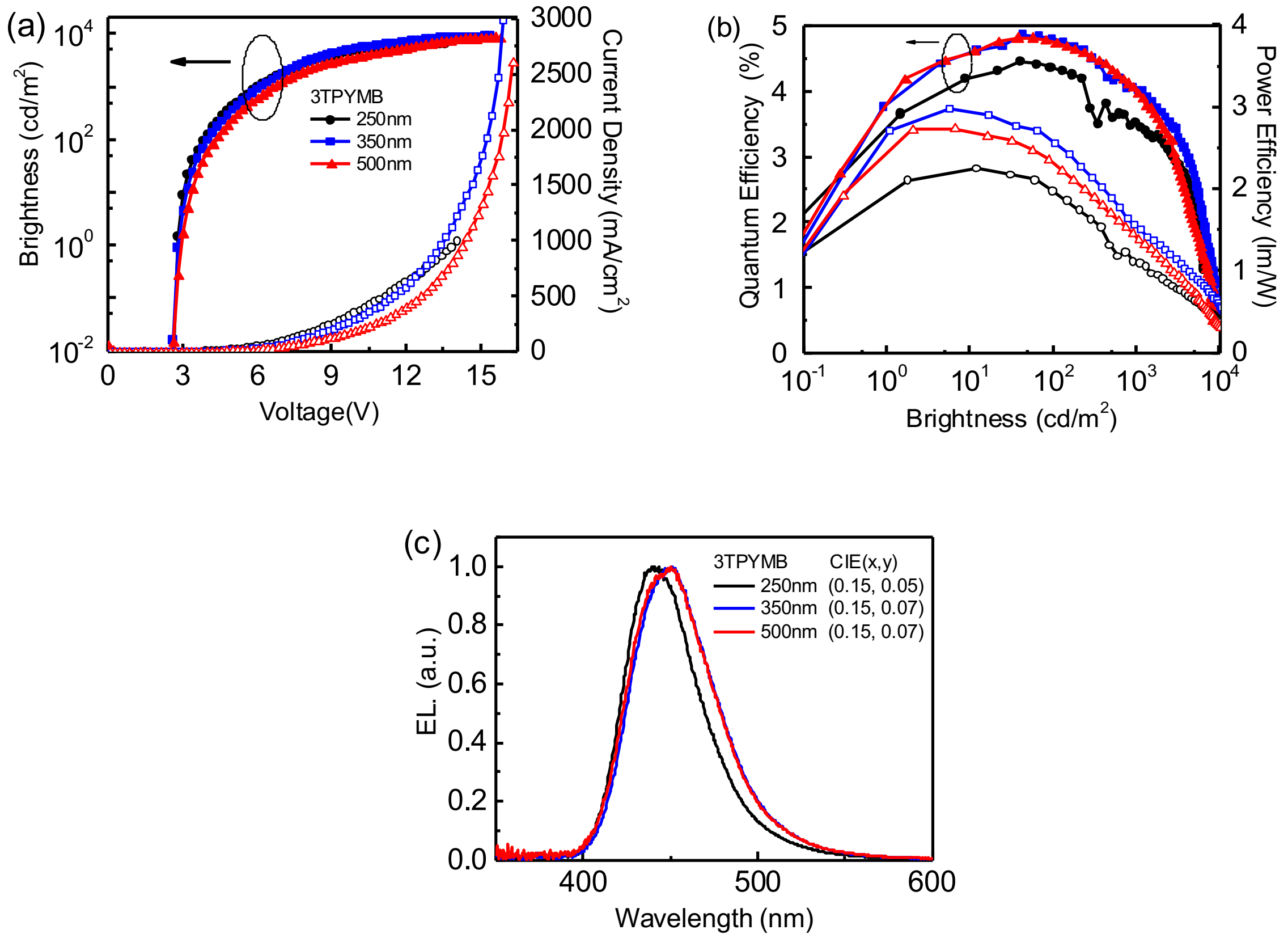

ETL	V_{on} [V]	L_{max} [cd/m^2]	I_{max} [mA/cm^2]	η_{ext} max [%, cd/A]	η_p max [lm/W]	At 100 nit[%, V]
25 nm	2.8	6517 (13.6V)	993	4.45, 2.3	2.10	4.4%, 3.8
35 nm	2.8	9553 (15.4V)	2977	4.86, 3.1	2.71	4.8%, 4.0
50 nm	2.8	8266 (15.8)	2607	4.80, 2.99	2.47	4.75%, 4.3

Figure S18. (a) Current density–voltage–luminance (*J*–*V*–*L*) characteristics of nondoped devices. (b) External quantum (EQE) and power efficiencies (PE) as a function of brightness. (c) EL spectra for nondoped device with architecture of ITO/Tris-PCz:ReO3(60nm)/Tris-PCz(15 nm)/**2**(250 nm)/ 3TPYMB(X nm)/Liq/Al.

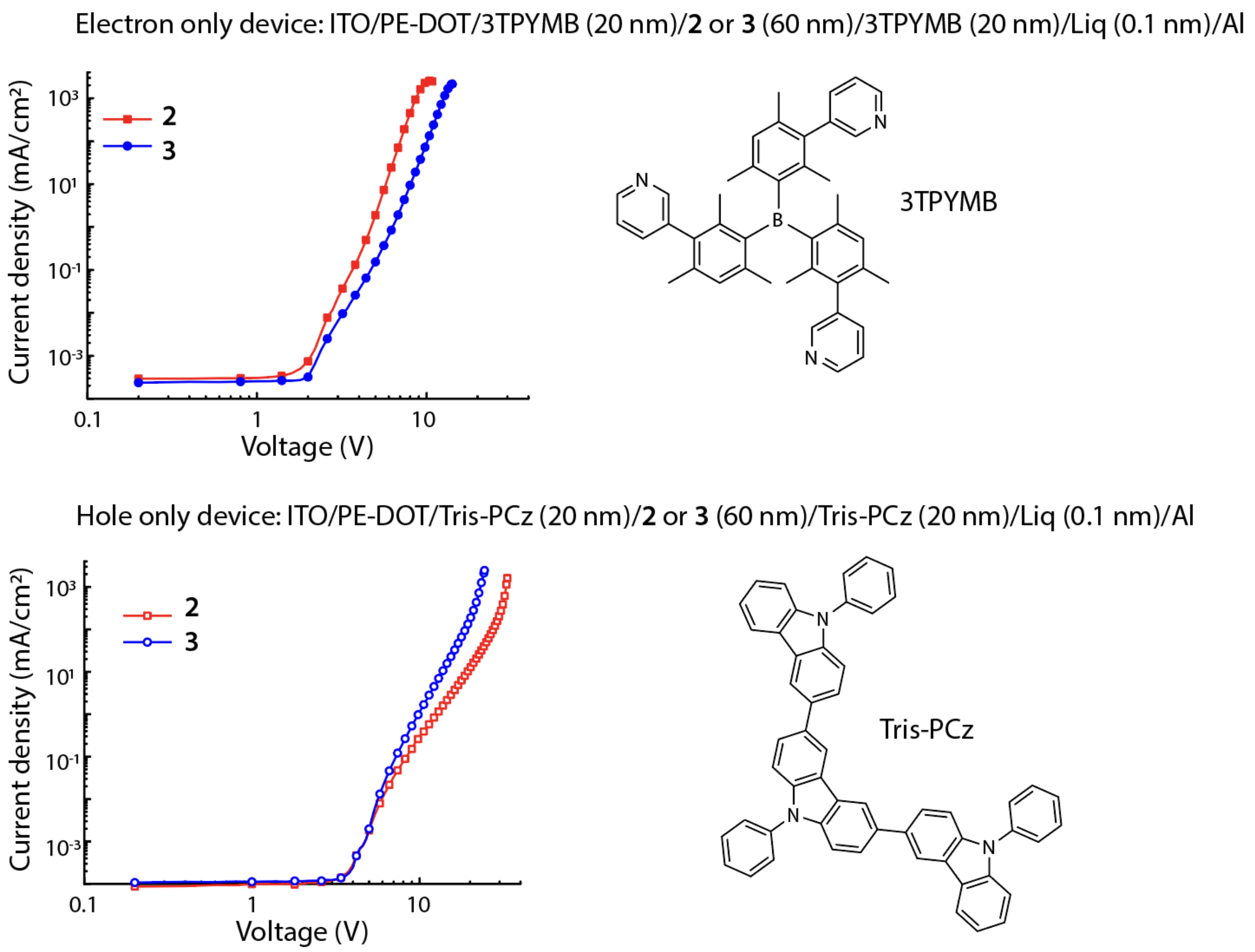

Figure S19. Current density versus voltage curves of the electron-only and hole-only devices for the compounds **2** and **3**.

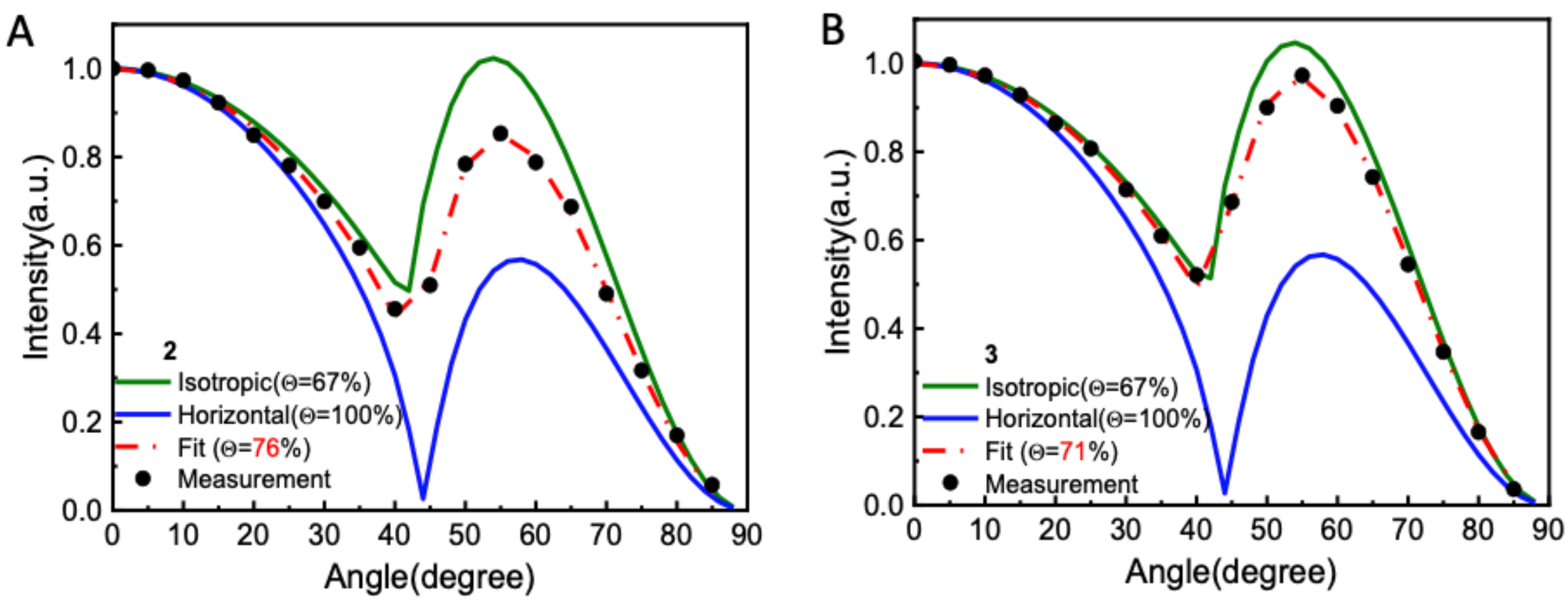

Figure S20. Angle-dependent PL intensity of *p*-polarized light at peak emission for (A) **2** and (B) **3** compared to simulated profiles (lines) with a different ratio of horizontal dipoles (Θ) [dashed line for fit, solid line for Θ = 1 (fully horizontal), and Θ = 0.67 (isotropic)].

NMR Appendix

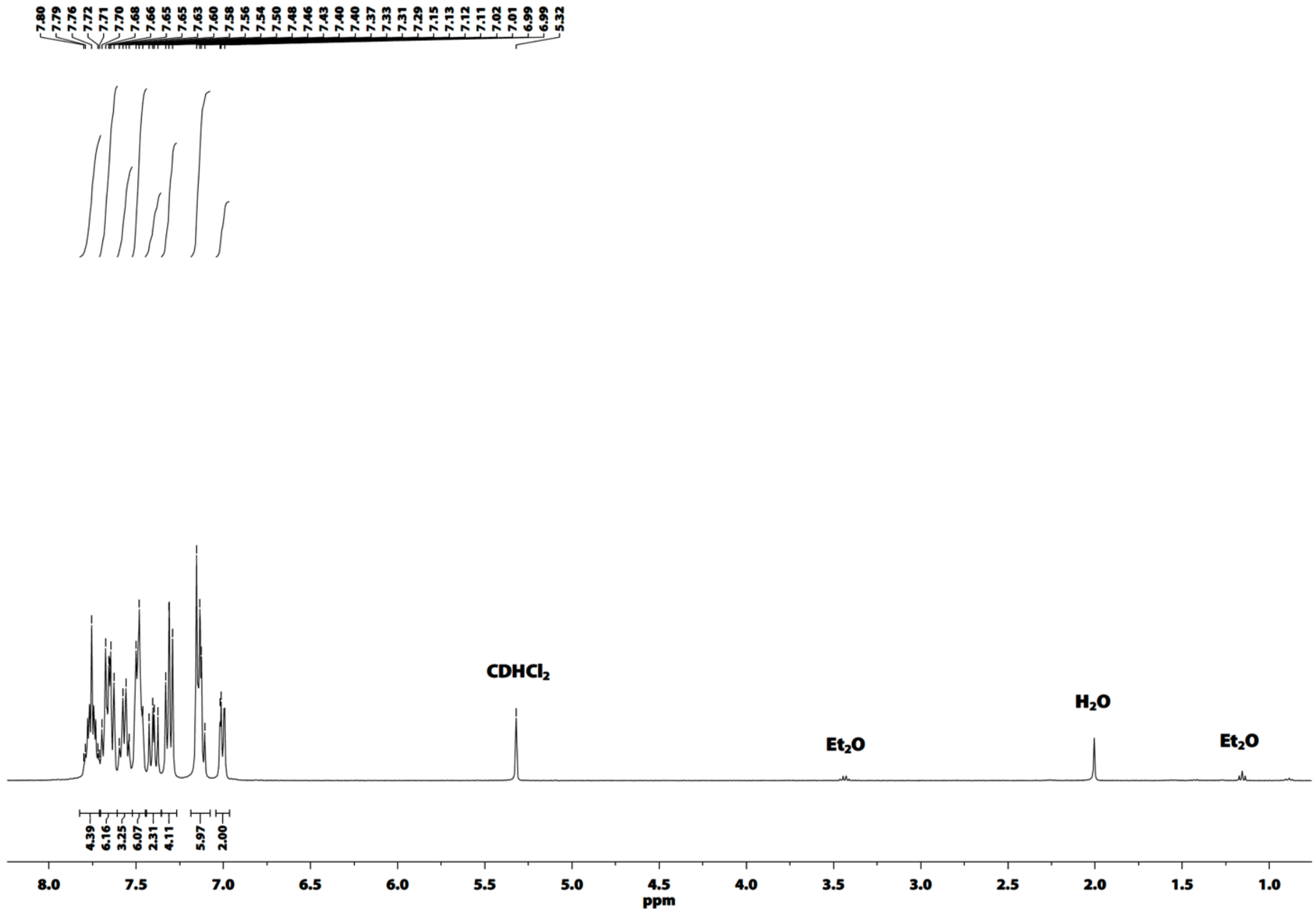

Figure S22. ^{1}H NMR spectra of **1** in CD_2Cl_2 (298 K).

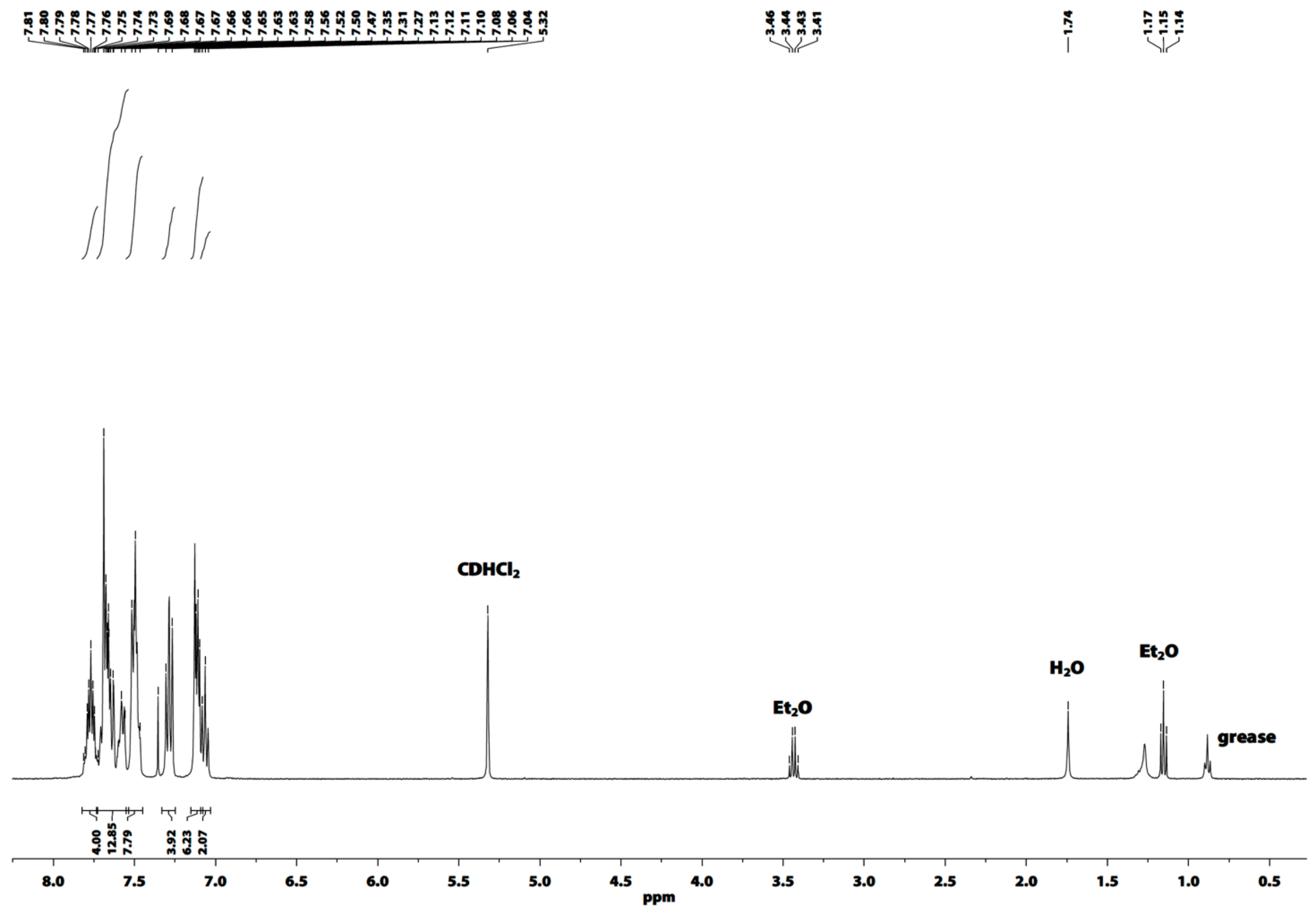

Figure S23. ^{1}H NMR spectra of **2** in CD_2Cl_2 (298 K).

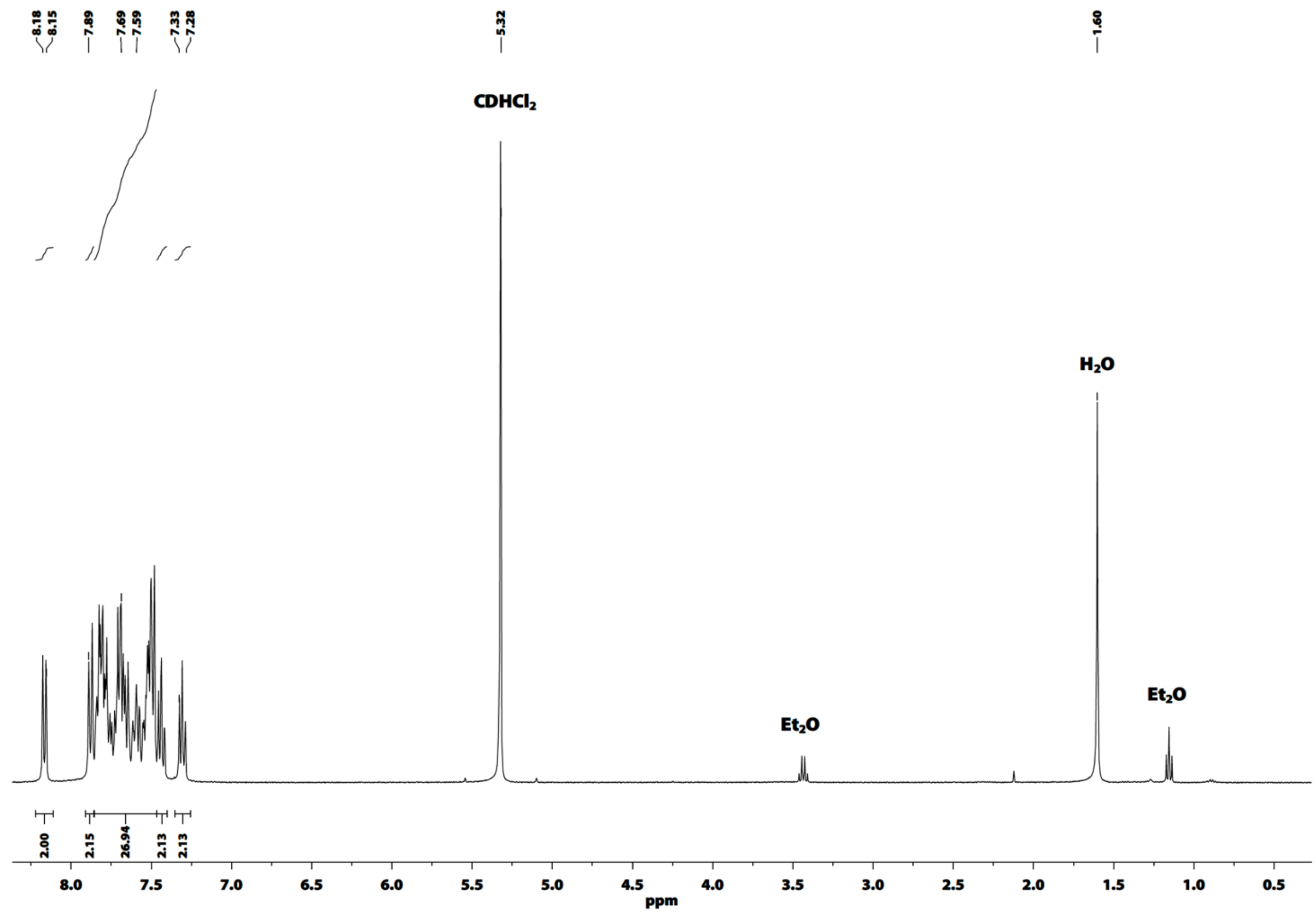

Figure S24. ^{1}H NMR spectra of **3** in CD_2Cl_2 (298 K).

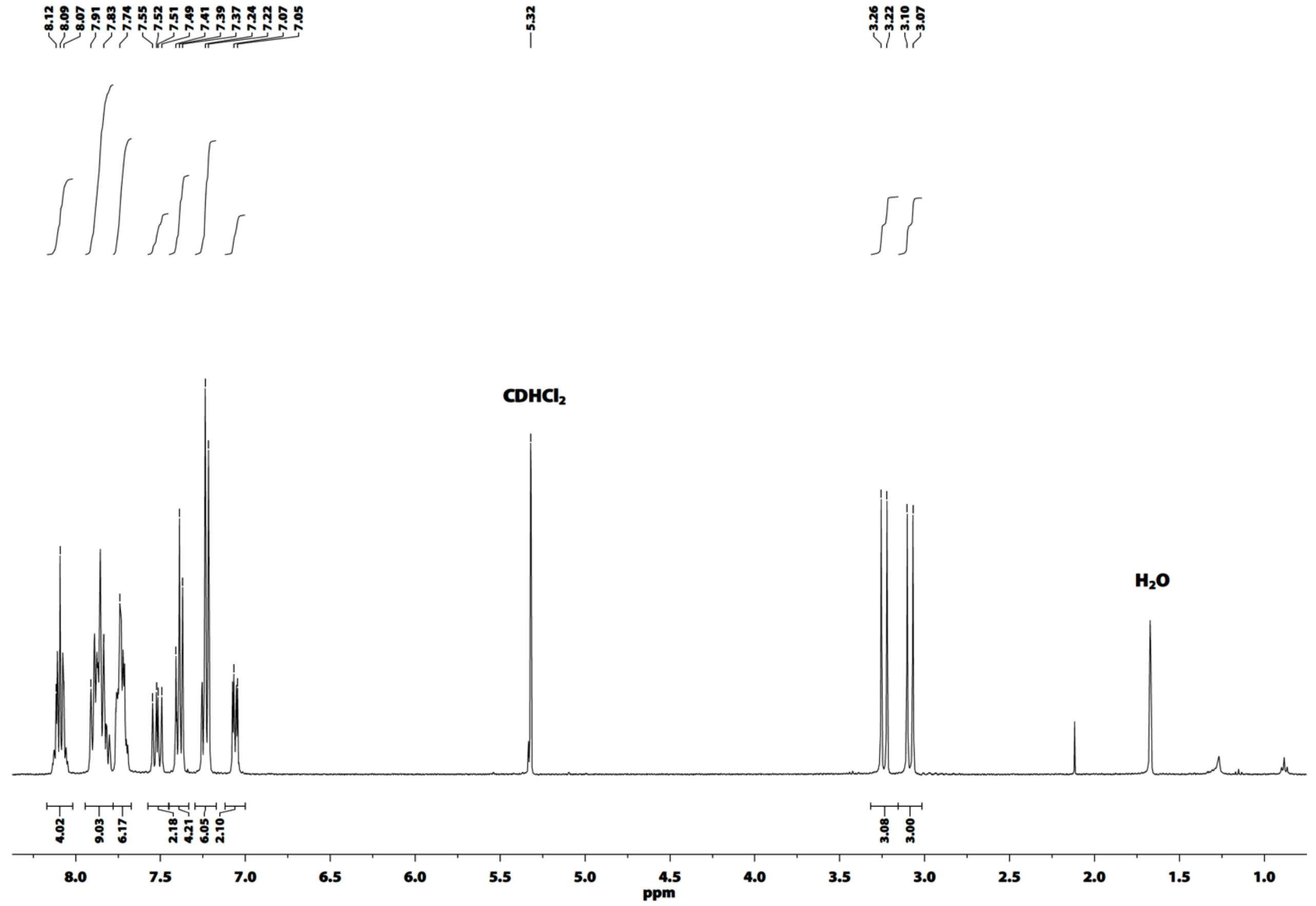

Figure S25. ^{1}H NMR spectra of **4** in CD_2Cl_2 (298 K).

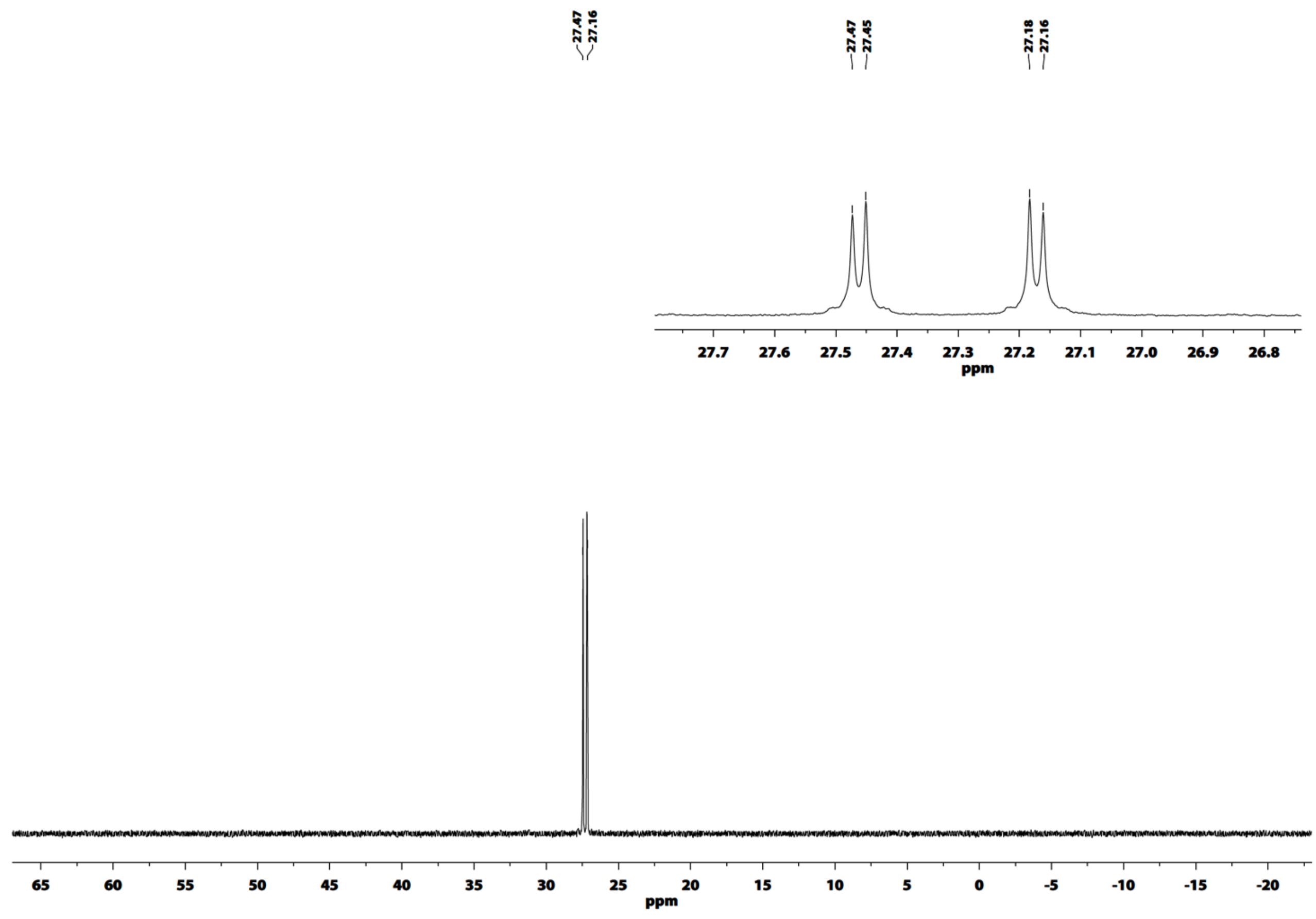

Figure S26. ^{31}P NMR spectra of **1** in CD_2Cl_2 (298 K).

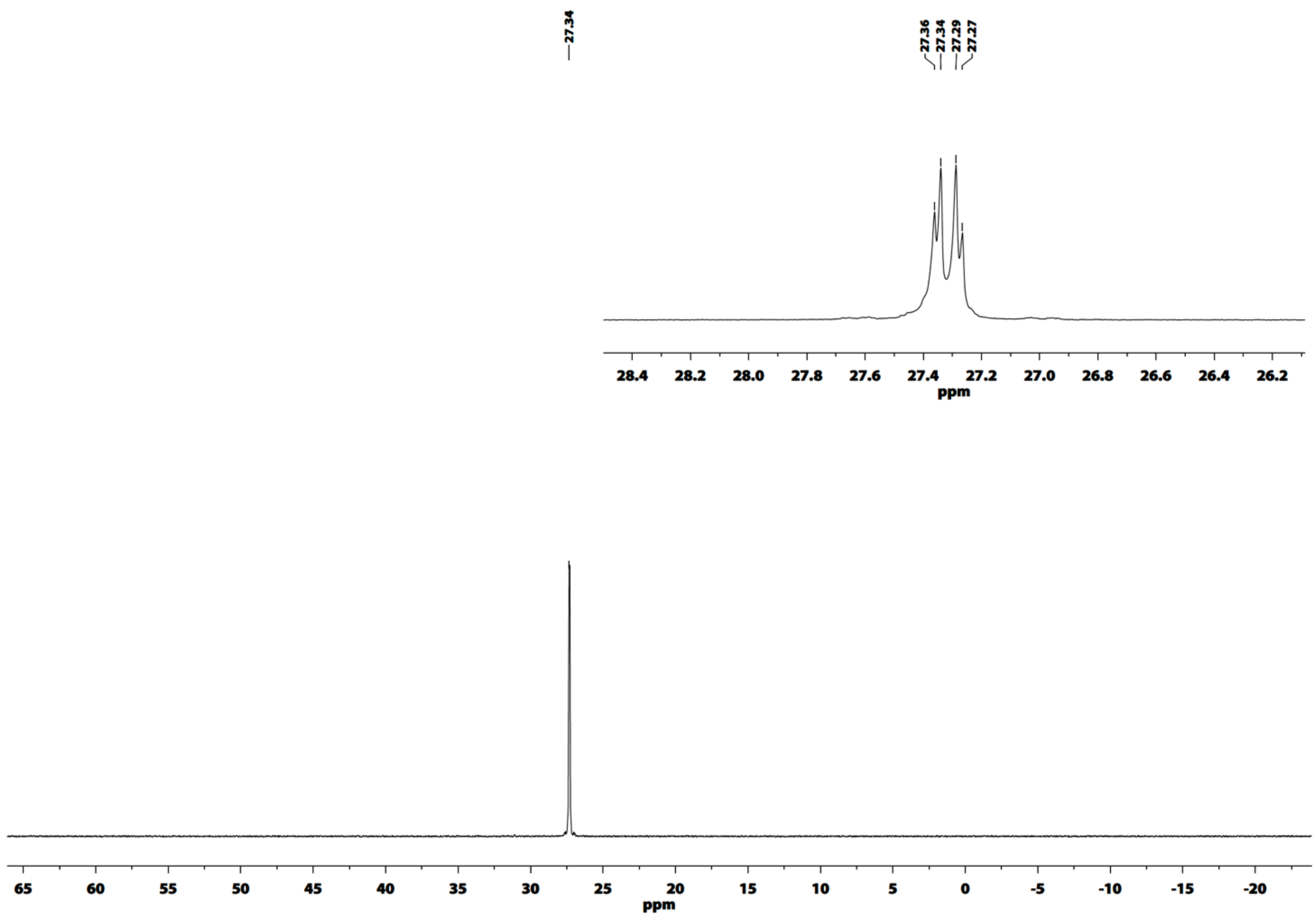

Figure S27. ^{31}P NMR spectra of **2** in CD_2Cl_2 (298 K).

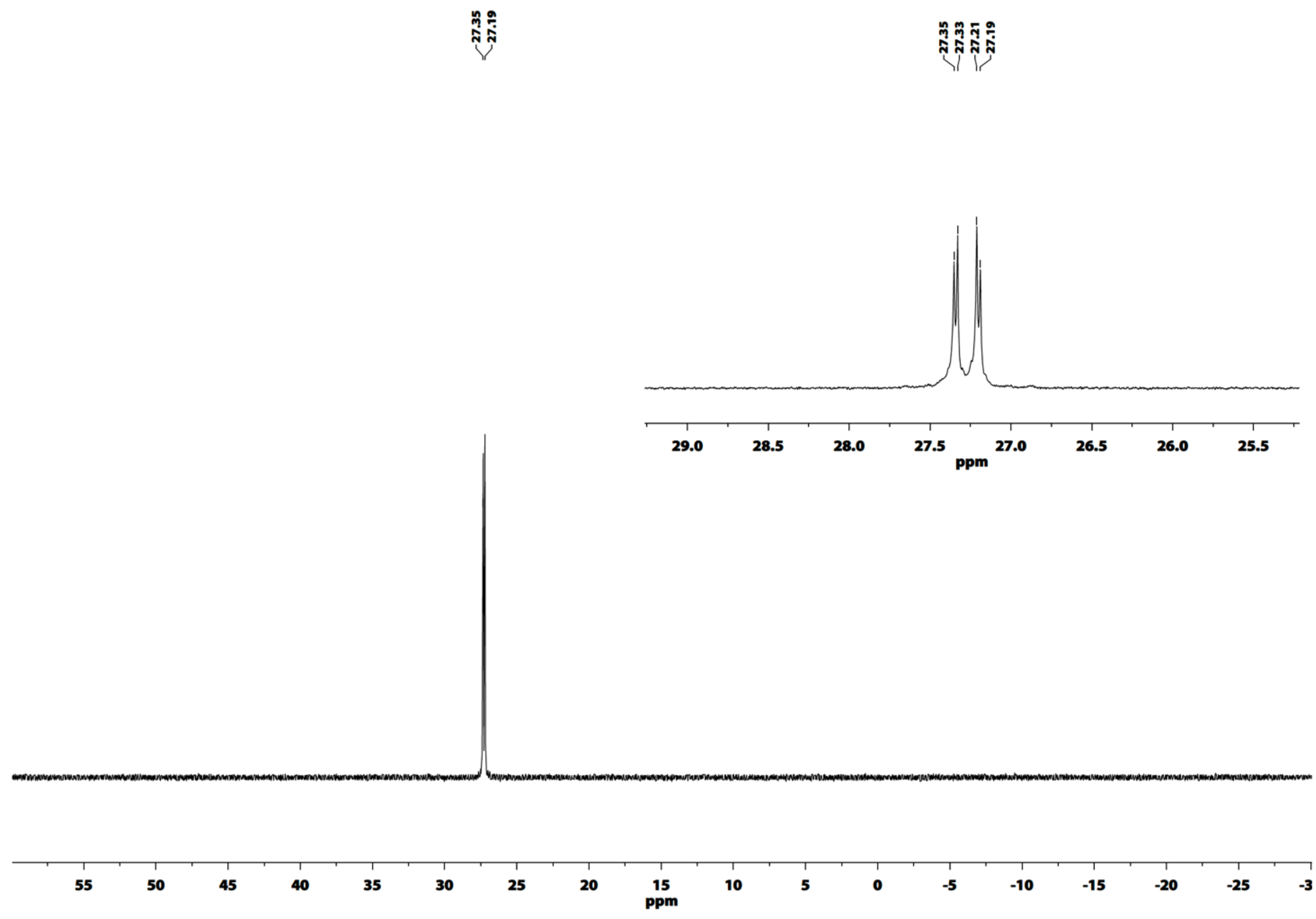

Figure S28. ^{31}P NMR spectra of **3** in CD_2Cl_2 (298 K).

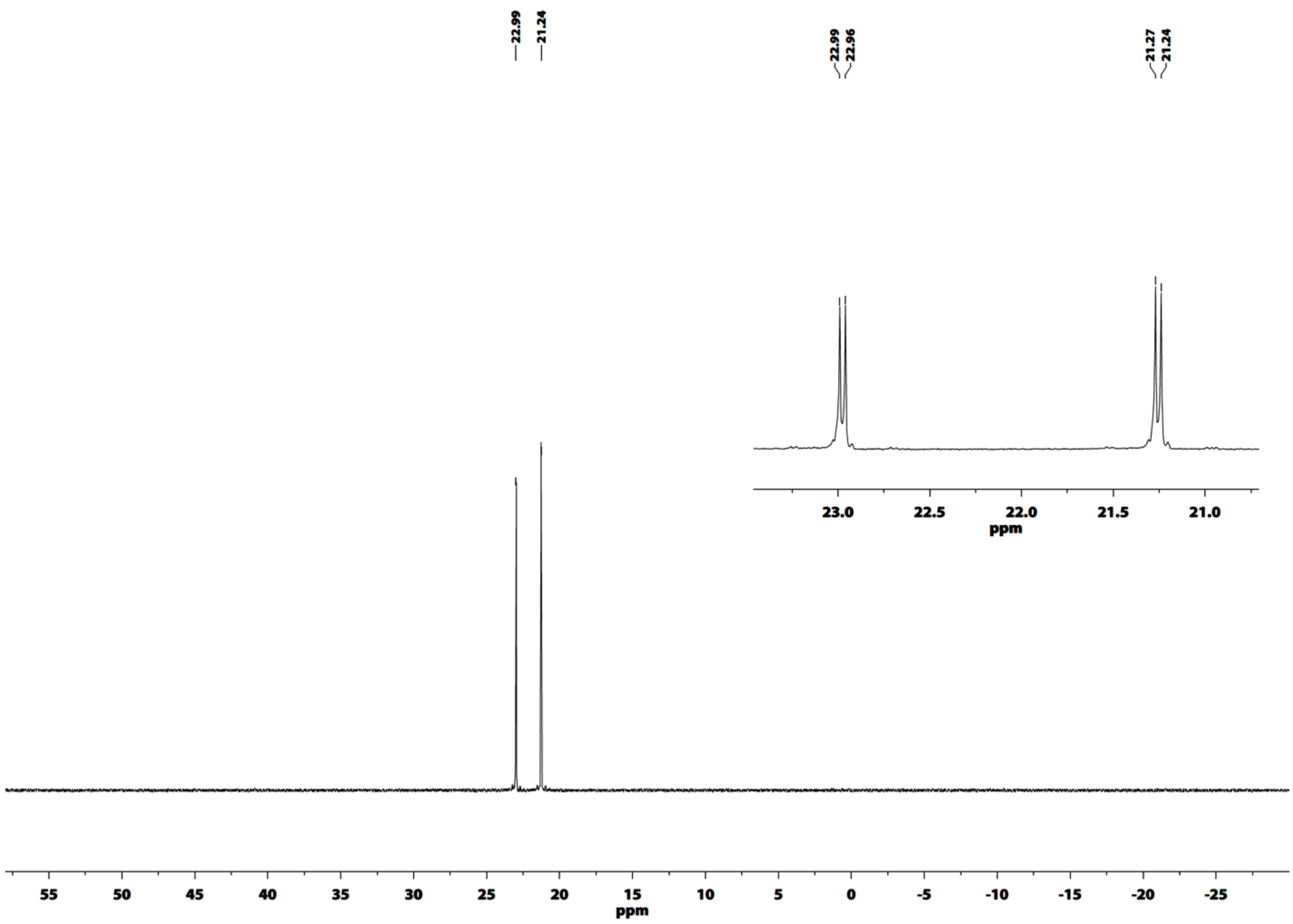

Figure S29. ^{31}P NMR spectra of **4** in CD_2Cl_2 (298 K).

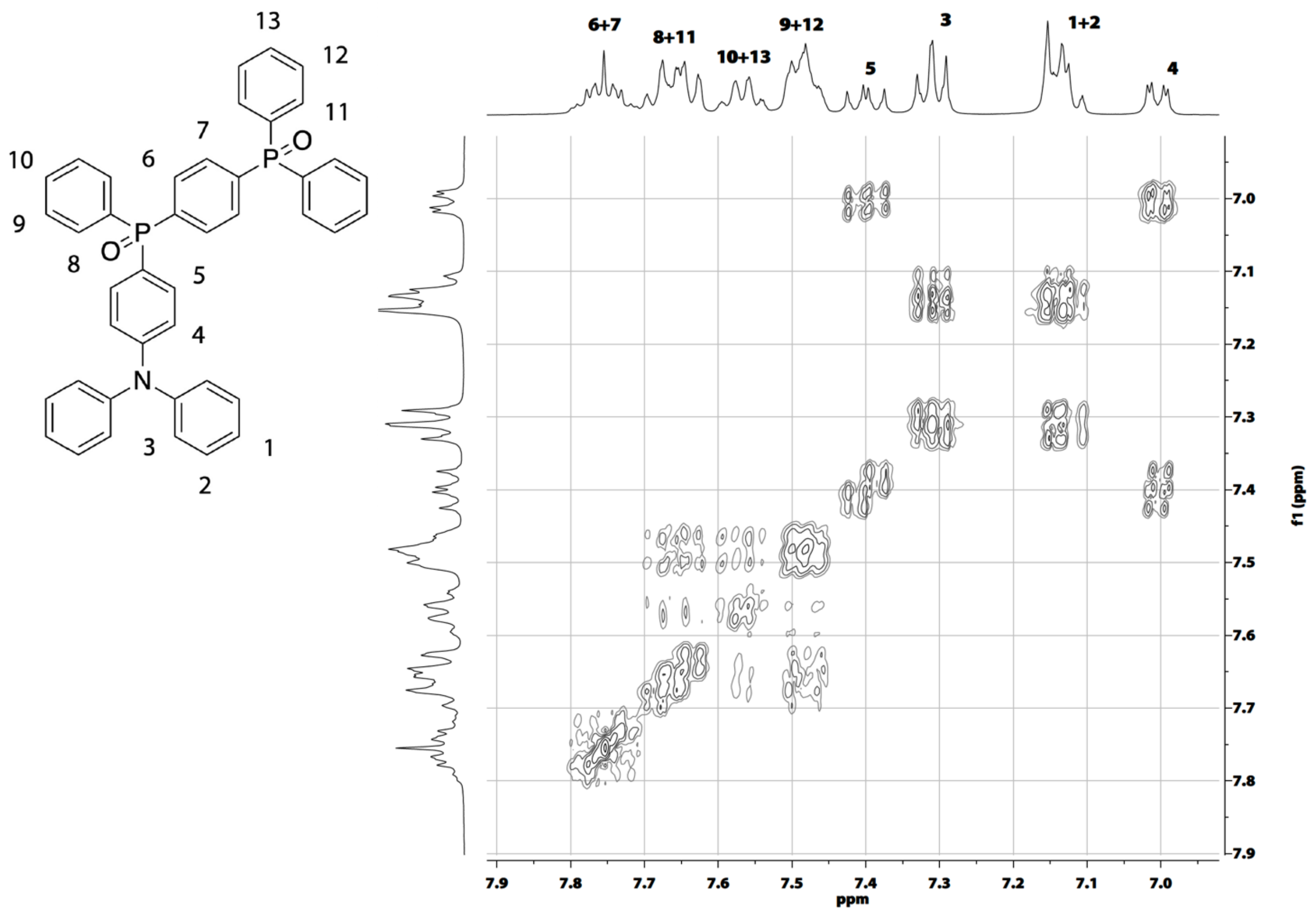

Figure S30. ^{1}H-^{1}H NMR spectra of **1** in CD_2Cl_2 (298 K).

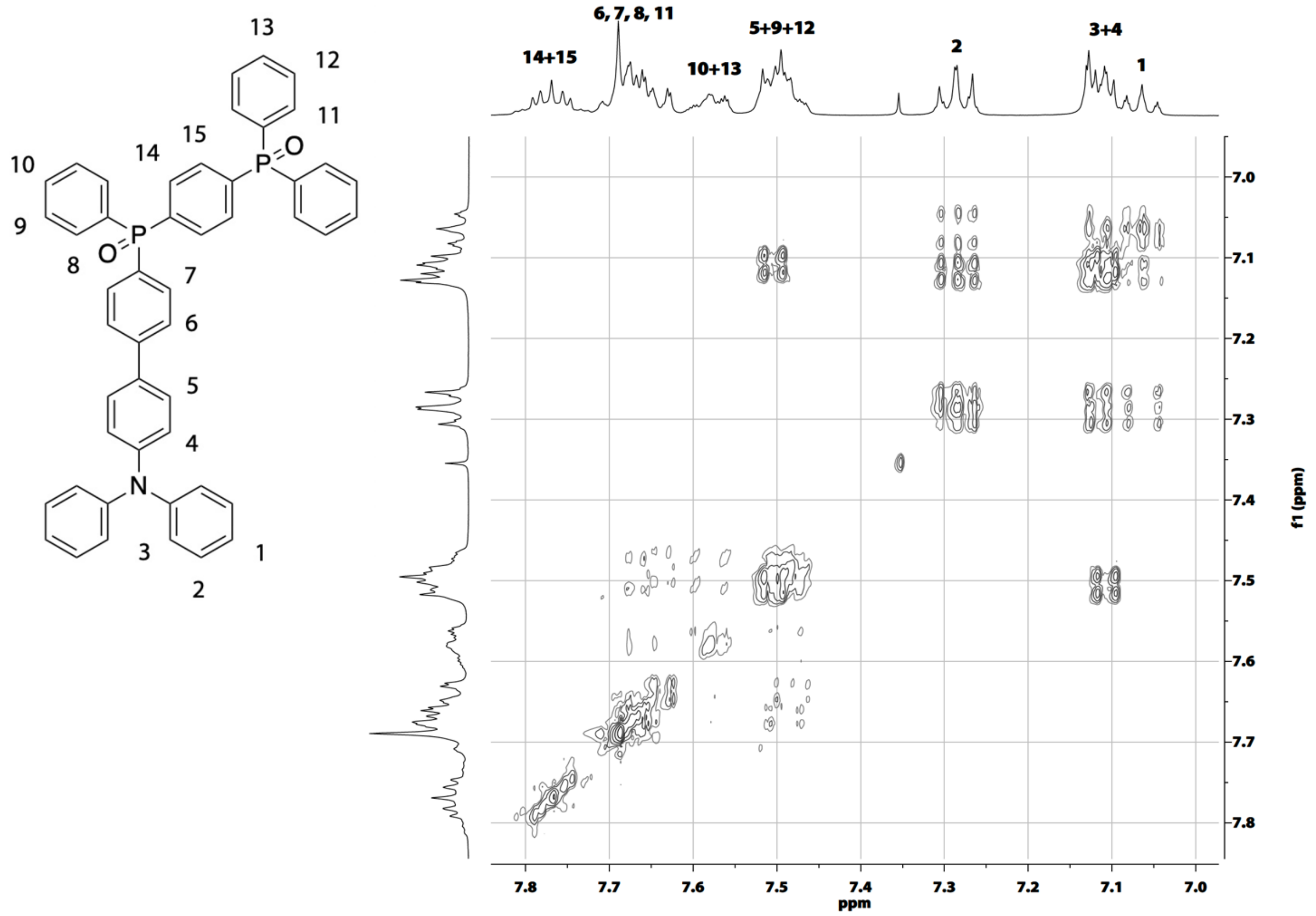

Figure S31. ^{1}H-^{1}H NMR spectra of **2** in CD_2Cl_2 (298 K).

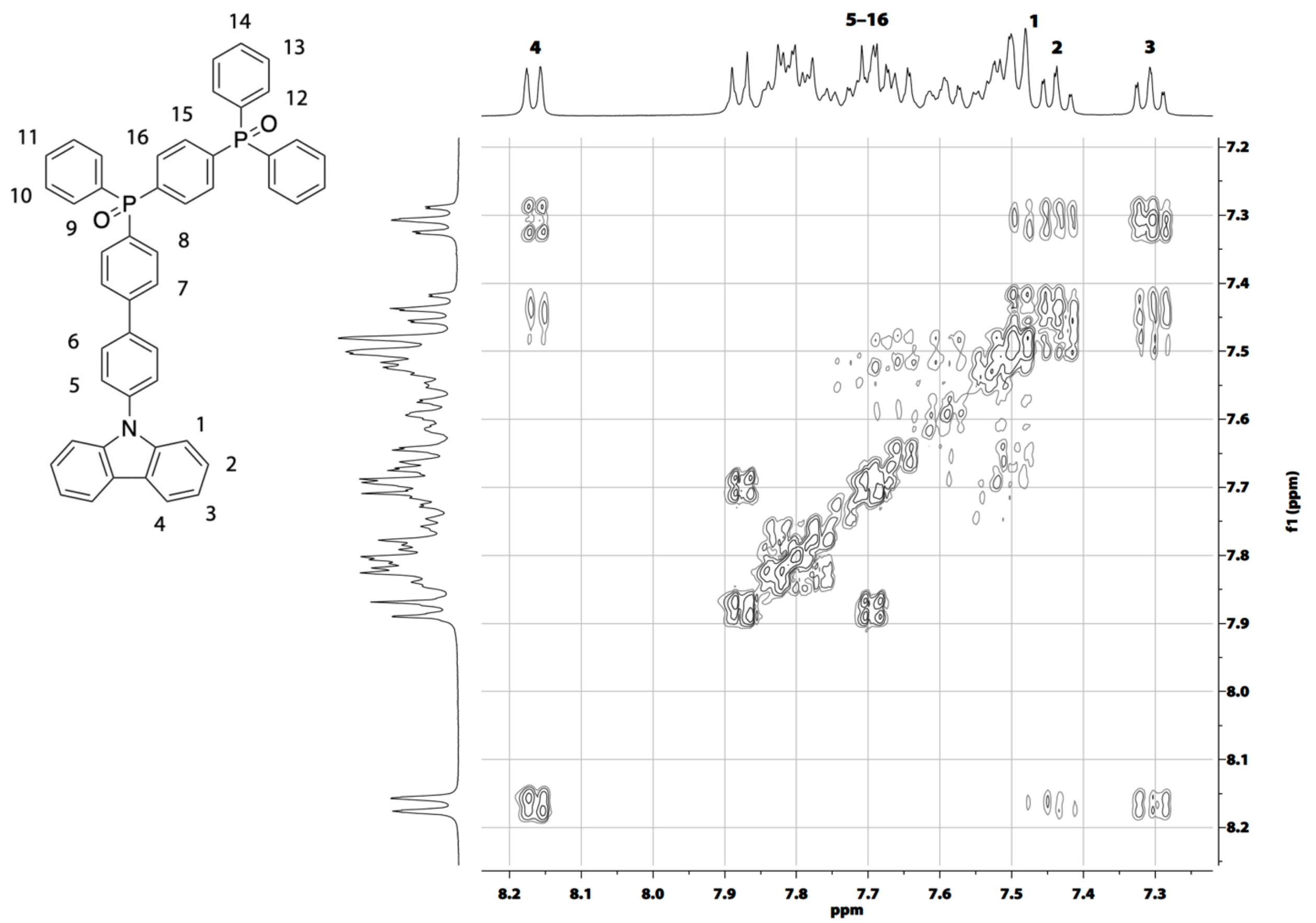

Figure S32. ^{1}H-^{1}H NMR spectra of **3** in CD_2Cl_2 (298 K).

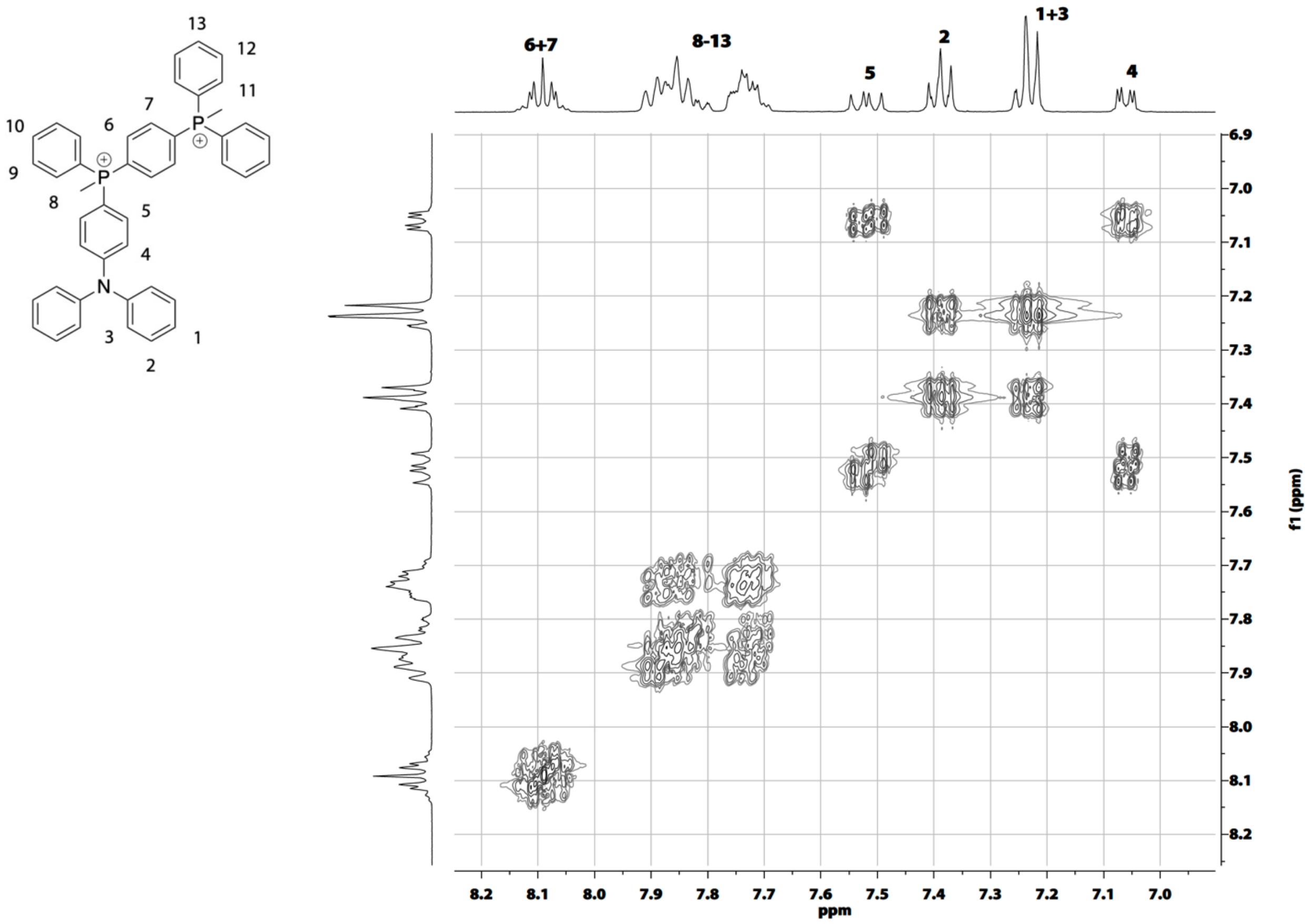

Figure S33. ^{1}H-^{1}H NMR spectra of **4** in CD_2Cl_2 (298 K).

Table S11. Cartesian coordinates of the ground state geometry of **1**.

P	12.31443207150070	4.60947295582973	8.29430252168161
P	11.57846819366251	3.09434287563032	2.11975050816074
O	12.95117354635205	3.50251152153931	9.06364865917106
O	10.91962729505556	4.19774248278350	1.36547881888881
C	12.13426096661517	4.21070811533718	6.53780023365065
C	12.24724625158680	5.16871946267999	5.53393248025704
H	12.47335730183502	6.19860863625010	5.78453587481223
C	12.07029971454737	4.80959784480631	4.20811203515711
H	12.15006164949208	5.55849416288727	3.42838577794542
C	11.78396891476330	3.48832495614575	3.87297413713331
C	11.67443097799640	2.52952501690031	4.87625159952745
H	11.44794348288216	1.49972563720959	4.62577851268794
C	11.85041692291107	2.88930953078144	6.20222776046163
H	11.76862897599853	2.14105017289790	6.98235269187923
C	13.27659340262702	6.13488315289603	8.35424620949238
C	12.67884607113989	7.39246957717307	8.34105871296149
H	11.59853486923300	7.48021285051749	8.31659145537697
C	13.46479897668822	8.53542500195528	8.36536006158956
H	12.99551467718698	9.51242925770448	8.35715500131902
C	14.84775318061134	8.42673299589522	8.40626428585093
H	15.46053500650879	9.32078255281963	8.42825487450521
C	15.44799778129336	7.17411952753144	8.42487247319136
H	16.52792002979395	7.08879329474843	8.46233613832536
C	14.66590857974155	6.03035384545869	8.39878783184641
H	15.13001742834674	5.05067297905377	8.42090420326397
C	10.64435596765716	1.55326424425114	2.06308520310542
C	9.25340720986276	1.63895567657723	2.03030077838971
H	8.77572493780998	2.61219263226150	2.01417938613632
C	8.48732842743864	0.48438436951793	2.00880042068938
H	7.40608279742039	0.55470099419203	1.98083207990295
C	9.10534177871791	-0.75952827170467	2.01988230979486

H	8.50511230264608	-1.66211417883134	2.00149831917438
C	10.49002351283943	-0.84901876825138	2.04869032407607
H	10.97297177936756	-1.81932903119086	2.05100453370881
C	11.26032641727830	0.30446615297437	2.06871085611233
H	12.34175482737803	0.23038700451495	2.08429241550527
C	13.22822681149901	2.70274986088680	1.50575451141514
C	14.28933083288694	2.38075117561309	2.34730517569059
H	14.15162061210371	2.37520000046558	3.42238653242290
C	15.53073962074370	2.06966234819131	1.81109399033993
H	16.35479959261024	1.82171886723707	2.47001577214726
C	15.71639055926993	2.08068939639790	0.43619680465314
H	16.68747181895096	1.83924584491072	0.01914488410095
C	14.66105318619536	2.40539542816708	-0.40707720277496
H	14.80685984202152	2.41830585583273	-1.48103607617129
C	13.42009623307914	2.71600307954132	0.12441447985790
H	12.59616805991637	2.97545191224948	-0.53111506822959
C	10.66639226234633	5.02887447982592	8.86516605742970
C	9.62302548135708	5.36338697142199	8.00440776619173
H	9.77034583258037	5.33606468719121	6.93087962177161
C	8.38272102763345	5.71727142205228	8.49876972723916
H	7.58103421446946	5.96219658570579	7.81361555765392
C	8.15104652924437	5.75387298288094	9.87699982941171
C	9.19685445703740	5.41222015654090	10.74274256610716
H	9.03728892632661	5.43711248117094	11.81334075707870
C	10.43112187931706	5.05439261150309	10.24111653049624
H	11.22997487713315	4.80068443051075	10.92942354546976
N	6.90490938069462	6.12512201243671	10.37652015015205
C	6.41863956325301	5.58105460596671	11.58454265625002
C	6.54344383711303	4.21866131944995	11.84784192615947
C	5.79119998628449	6.40322915636460	12.51771792045172
C	6.05837504887436	3.69253961249332	13.03402085722858
C	5.29430483919470	5.86685322528306	13.69467175393161

C	5.42855497407609	4.51107958275259	13.96239704554515
H	7.02080633200443	3.57463202423641	11.11876486684502
H	5.69253619205463	7.46295498637864	12.31416995276531
H	6.16121961019119	2.63053128834861	13.22628044810152
H	4.80860880017297	6.51787528012776	14.41276624914704
H	5.04376959269967	4.09512015004881	14.88607538166250
C	6.10193205615971	7.05325656023187	9.67742953845550
C	4.74126178758194	6.81104140197598	9.50682210832362
C	6.65853599349090	8.22401317052592	9.16753168713457
C	3.95002165317370	7.73103940981556	8.83756573138543
C	5.86437442642697	9.13169765011095	8.48604138830751
C	4.50637449683259	8.89298954521731	8.31982057548823
H	4.30717246809222	5.90044301556997	9.90281363289262
H	7.71539531118930	8.41809502974352	9.30795128673535
H	2.89213058161890	7.53083558879038	8.71046406166884
H	6.30920521904039	10.03953419163782	8.09440976893118
H	3.88635658526969	9.60810932545213	7.79213272440139

Table S12. Cartesian coordinates of the S_1 geometry of **1**.

P	12.32262719643421	4.89560932065275	8.15502436220352
P	11.68582971631890	2.95258654308285	2.11612823769605
O	12.91901287973303	3.85613750973332	9.08572105217074
O	11.12573039743091	4.01821564360360	1.19066361720660
C	12.16689386186213	4.40652164290068	6.47908949421245
C	12.05967470223434	5.31860980707850	5.37138342711739
H	12.11589263088448	6.39303530664833	5.54174287489774
C	11.90258677834642	4.85437188103863	4.08779363984983
H	11.82257099653006	5.56088441187408	3.26189687546989
C	11.83537680185048	3.45488247352448	3.80038477678570
C	11.94206164325541	2.54440567811757	4.90533925566486
H	11.88806608349889	1.46997374468888	4.73119010015102

C	12.10063068663763	3.00265425252797	6.19002203941860
H	12.18451327898417	2.29317918475095	7.01268497968702
C	13.22917254948250	6.47580250449202	8.22757072579196
C	12.67830979632373	7.70810402163614	7.85285939778794
H	11.63923937555445	7.77457640050688	7.53054291612631
C	13.45688168916258	8.86495991883993	7.90193641161697
H	13.02573218978947	9.82067967115264	7.60607944456809
C	14.78198624447276	8.79912588051754	8.33589120143995
H	15.38702017529897	9.70418607162550	8.37635497098279
C	15.32928453657893	7.57441941973065	8.72583531293564
H	16.36015541457816	7.52264516647550	9.07439403044746
C	14.55491803498679	6.41638329207578	8.67326822029409
H	14.96561748070446	5.45536471335712	8.98209366887822
C	10.65269948256921	1.44582809241409	2.08933610625604
C	9.30495356011503	1.60775785961757	1.74673012895906
H	8.94451979184545	2.60083611594143	1.47893045098919
C	8.44569113901636	0.50999148668027	1.73808768407700
H	7.39875631588884	0.64154147506787	1.46638346562229
C	8.92859492601501	-0.75780638032485	2.07194114187709
H	8.25772361961809	-1.61622615743281	2.06485203534307
C	10.27369139522668	-0.92599534911860	2.40486932413744
H	10.65492643721515	-1.91549995802236	2.65500993832228
C	11.13630444905131	0.17127182817970	2.40797382680484
H	12.19044367618325	0.02910720575060	2.64674089883957
C	13.31039784540982	2.37896444217974	1.49678442276174
C	14.32282903230693	1.93167461401432	2.35269832536142
H	14.15728632646137	1.92448468772029	3.43022633202935
C	15.54699872709744	1.51128711845722	1.83006434065515
H	16.33210791079163	1.16242347176041	2.49998645074336
C	15.76827304722945	1.54861235537177	0.45212184114098

H	16.72606183239253	1.22569302642606	0.04529207845831
C	14.76558682794301	2.00915339730426	-0.40446236472944
H	14.94183199934992	2.04977659047811	-1.47897439607174
C	13.54021281661086	2.42325791784635	0.11617378179845
H	12.75507782863584	2.79690111481208	-0.54068860858432
C	10.65952636659508	5.34715103828939	8.81055795690098
C	9.61636876278438	5.77493024127801	7.97740710928572
H	9.77773354838340	5.85300548165955	6.90274598927267
C	8.36737136512022	6.06044474713579	8.50490252939064
H	7.54605883144023	6.36334977151974	7.85873224953716
C	8.14475515724027	5.89033678639720	9.88607851952259
C	9.18179841467294	5.44055556764745	10.72630581384627
H	9.00409240780069	5.33483063207009	11.79467048829557
C	10.42924692429724	5.18072545912911	10.18497059056571
H	11.24506089702478	4.84060929945514	10.82073680682897
N	6.88406257838192	6.15889744083743	10.42263230976864
C	6.37036305220057	5.34558814692697	11.44207351366808
C	6.57943273730042	3.95524762736993	11.39535512113610
C	5.65164548140466	5.92450620466847	12.50349955680280
C	6.06920000727929	3.15534967328012	12.40789362714017
C	5.15236906500050	5.11108469737577	13.51061582097021
C	5.35776227447385	3.72826098604487	13.46662811806205
H	7.10845476955917	3.51686879008222	10.55158613230030
H	5.52444694499446	7.00434348362406	12.54410023738895
H	6.21505433237428	2.07778917625423	12.36576093872040
H	4.61264611747456	5.55736157574215	14.34358920091016
H	4.96238756603863	3.09584572194170	14.25939393583330
C	6.13586409066427	7.24102949210454	9.94471478765864
C	4.74018142998045	7.12233779804950	9.80967652790139
C	6.78637956700638	8.44095149027162	9.60109860575336

C	4.00863792468586	8.19867227326657	9.32984523467290
C	6.03922922900682	9.50936542702941	9.12664542846871
C	4.65268894750394	9.39272055071258	8.98824215588882
H	4.25281910902493	6.17916913271223	10.04754435110327
H	7.86108203778502	8.53217084716235	9.74421621613450
H	2.93172044578192	8.10353666181808	9.20594083216317
H	6.53745151698892	10.44397985454628	8.87678834261158
H	4.07221159081940	10.23396245083977	8.61401967330184

Table S13. Cartesian coordinates of the T_1 geometry of **1**.

P	12.52318292400198	5.32715734290332	8.22309722185251
P	11.35077834813256	2.54804687926557	2.54376735109226
O	13.28324080348876	4.37710767537196	9.12485729751994
O	10.45493660813907	3.42507369145016	1.70013330809827
C	12.25961758919716	4.58910658681008	6.57929643618018
C	11.82525957696756	5.32631803692402	5.46638441346725
H	11.70945160116861	6.40790524130341	5.53079463304890
C	11.55320131708397	4.67990608336579	4.26510063018484
H	11.21288086608837	5.24382536689160	3.39735336809033
C	11.70926169318003	3.29114082903153	4.15999289820166
C	12.12562441835535	2.55072840396899	5.27539353758526
H	12.22996393099950	1.46771286941528	5.21296549165571
C	12.40507435055518	3.19866541432939	6.47447113789962
H	12.74135768219092	2.63752067958739	7.34558859554350
C	13.37690351809161	6.90266421458195	7.91043980436443
C	13.10174654145886	8.02388756161328	8.70252430293020
H	12.31573328990622	7.97481624833800	9.45629232517660
C	13.82667408225496	9.20190345039522	8.52501045264762
H	13.60422057719421	10.07334729462979	9.13940000264263
C	14.83401696805419	9.26283134923260	7.56078478779256

H	15.39842559635041	10.18402591362254	7.42086644039444
C	15.11960928196654	8.14408433098065	6.77521520109389
H	15.90744977793272	8.18996403820023	6.02444816580003
C	14.39419847657129	6.96677706479710	6.94926065988264
H	14.61695384299382	6.09604302037481	6.33229215658934
C	10.60387507525393	0.92532597371292	2.87516907341262
C	9.20521801226672	0.86176445343208	2.85250067118721
H	8.63428461016390	1.76070543019152	2.62252864544350
C	8.55897460725583	-0.34630644798381	3.10817937251135
H	7.47095545989552	-0.39427168401757	3.08782808289840
C	9.30651988999652	-1.49318029031001	3.38300329643244
H	8.80087663565661	-2.43766721977298	3.58030958311414
C	10.70117777634420	-1.43394047461543	3.39610064610783
H	11.28445536009108	-2.33084742747464	3.59988363069960
C	11.35240791668211	-0.22762028821053	3.13851501453469
H	12.44155267547839	-0.19126857325938	3.13204344786742
C	12.94783886781506	2.22833070178705	1.73888269271431
C	14.12683209262594	1.95150748010160	2.44040296833488
H	14.13104863543473	1.93737458430654	3.52975001181830
C	15.30875461817576	1.70508717354387	1.74188790649320
H	16.22548506906907	1.49050697243918	2.28935214660260
C	15.31811998157840	1.74259292204345	0.34655767750565
H	16.24356779426402	1.55338662683468	-0.19598139909602
C	14.14550770118936	2.03125949317001	-0.35432531923803
H	14.15422146904970	2.07081446548603	-1.44281221926849
C	12.96180086790250	2.27622199507827	0.33925306686772
H	12.04360277057893	2.51695648793851	-0.19543789870215
C	10.89884955286053	5.72091196451548	8.84294634700155
C	10.04489460275000	6.72382264856239	8.24274102188202
H	10.42424288741360	7.35035649244920	7.43700505851811

C	8.76045907664982	6.89059686635806	8.67693592115798
H	8.11413640986788	7.63161040692888	8.20838819757955
C	8.22659885307784	6.06213225390077	9.72215039324553
C	9.07770326393385	5.05421645235000	10.32180633160400
H	8.69762580901427	4.46569833099532	11.15510121056824
C	10.36764905384575	4.90495233261617	9.89439028760596
H	11.02404423266764	4.17229082057220	10.36183603692806
N	6.92017269616748	6.22202200208438	10.16013513500931
C	6.14835162918810	5.09374280549529	10.46134172287716
C	6.32416538707720	3.91212614499828	9.71291154927310
C	5.23886170546283	5.11086856846668	11.53813056510945
C	5.59117119265467	2.77617620627564	10.03395408762802
C	4.51407074336871	3.96899457161028	11.84505077020549
C	4.68447644617625	2.79550267628700	11.09744207035703
H	7.01199473062641	3.91564129517749	8.86953305369058
H	5.12973198633130	6.01366908287894	12.13600594851960
H	5.71964492467876	1.87137252553097	9.44184271103203
H	3.81957988086657	3.98494092924754	12.68365324398934
H	4.11381828944730	1.90238793318255	11.34618245792653
C	6.38165939125795	7.51057139479320	10.29272406534939
C	5.04401733671941	7.76680544276674	9.93540386166503
C	7.20068361616848	8.56426765955454	10.74034444325967
C	4.53805951614013	9.05535490327095	10.04060001001363
C	6.68034950292505	9.84842818002335	10.84068392489322
C	5.34987700000608	10.10230585126448	10.49295621348164
H	4.42326082198585	6.95672346273843	9.55746236544532
H	8.22874470629842	8.35217113322757	11.02796468086559
H	3.50568450912667	9.25014907165846	9.75408789195375
H	7.31395575913934	10.65628633040285	11.20347127197912
H	4.94748055600897	11.11093926100745	10.57043239140609

Table S14. Cartesian coordinates of the ground state geometry of **2**.

P	11.86612076049116	4.49515707052917	7.98406903102388
P	11.51958702667508	2.12536011496251	2.05015035495738
O	12.47806573173414	3.52525457866834	8.93572749505646
O	10.72656585443754	3.01312296582746	1.15380027499481
N	2.29696365529434	7.03754545173262	10.81077504550464
C	11.79327080115504	3.84996041978376	6.29526675240655
C	11.89966016303032	4.67182684266546	5.17576273871799
H	12.05818538412492	5.73765749415813	5.29398448494346
C	11.80720349174856	4.12919763185144	3.90547468743408
H	11.88418091112655	4.77213651291235	3.03579970798119
C	11.61444326463256	2.75886026293399	3.74176292859452
C	11.51193673534856	1.93746664249160	4.86056824135617
H	11.35987461538974	0.87092259565813	4.74366036134951
C	11.60117301329504	2.48112664596918	6.13194809085355
H	11.52607138220129	1.84019002031158	7.00300214260106
C	12.78332803408353	6.04349446315557	7.86464986610472
C	12.16361688497971	7.28935468319107	7.87379172182392
H	11.08607647766286	7.36114670943110	7.96400408049875
C	12.92570925536807	8.44490316805445	7.77117496967771
H	12.43876600660053	9.41318231438587	7.78119114453525
C	14.30568458236055	8.35998297416858	7.65957525542031
H	14.89923133589089	9.26365732689581	7.58008863935716
C	14.92907481777713	7.11797330596217	7.65301866311604
H	16.00760289760282	7.05071907517697	7.56897984459378
C	14.17196977390233	5.96294748171703	7.75563700626762
H	14.65836892550031	4.99344881345144	7.75562360614171
C	10.82917639940129	0.46515068243522	2.17983770449802
C	9.44317884971320	0.33266654753515	2.11293023999842

H	8.82655927517736	1.21143079399352	1.96107136656188
C	8.85941700071442	-0.91867023057343	2.23091292331262
H	7.78155252366646	-1.01875369643959	2.17610455800393
C	9.65539566598702	-2.04173931885793	2.41604627746490
H	9.19773172353799	-3.02028360823790	2.50666916499668
C	11.03598592261375	-1.91428356096681	2.47971545684314
H	11.65796383519565	-2.79104273339528	2.61806220617400
C	11.62402170358915	-0.66367847939738	2.36078708714301
H	12.70291684874318	-0.56867418563756	2.40510247825705
C	13.22899128943227	1.92543898986243	1.51327280894031
C	14.28557472569888	1.79174231549027	2.41022947035780
H	14.10299634470905	1.82771006991207	3.47818245585316
C	15.57831904807312	1.61753565014926	1.93816614097198
H	16.39853417355080	1.51624399042454	2.63937423147522
C	15.81991742275399	1.57957165032666	0.57210754930198
H	16.83124240406324	1.44656068426606	0.20501490848540
C	14.76911577637146	1.71758473304348	-0.32589272943846
H	14.95839928069018	1.69326190737296	-1.39284310082946
C	13.47623737334602	1.89030397364347	0.14171511630978
H	12.65396143749457	2.00604463338327	-0.55557415910044
C	10.17225288725649	4.92386260075405	8.41395492759932
C	9.22616423983107	5.30963848886207	7.46732852343286
H	9.48333262022785	5.33322792569915	6.41445286896414
C	7.94447541364771	5.65071426224394	7.85947044484587
H	7.21610106320084	5.92485384481606	7.10539989975025
C	7.56879768435882	5.61431489423279	9.20504013976380
C	8.52397255282710	5.21896909784963	10.14552024872148
H	8.26457284956863	5.19628036899796	11.19748961258451
C	9.80701088859054	4.87829771033925	9.75782572299044
H	10.53509191125148	4.57400935931619	10.50124022075502

C	6.20459808884270	5.97876031111848	9.61912100903093
C	5.48258105888325	6.96364794620970	8.94197810947724
H	5.93296376700993	7.48029842661210	8.10211912748728
C	4.20203262420331	7.31082624405876	9.32632677300126
H	3.66731643692483	8.08021405674127	8.78283854502449
C	3.59306063210898	6.68391640967507	10.41471405887843
C	4.30692089960855	5.69896388976127	11.09924484658179
H	3.85218422716177	5.19680861116474	11.94439437834875
C	5.58530651900018	5.35519908904666	10.70407895313061
H	6.10755714595497	4.57651524237996	11.24822967883599
C	1.87180164181715	8.37820730942228	10.72722562124471
C	2.72076778138154	9.41457663565076	11.11239161875819
H	3.71343760691039	9.18268827086481	11.47976833615144
C	2.29789675735950	10.73089738679423	11.02552651718066
H	2.96901063758738	11.52611739419835	11.33020582040784
C	1.02118253600910	11.03297830171015	10.57027915240947
H	0.69101135763208	12.06326357933039	10.50946806735147
C	0.17182010013994	10.00047593414776	10.19482581218899
H	-0.82563588706964	10.22151211317334	9.83175829271689
C	0.59280326020093	8.68237416880622	10.26422087527357
H	-0.06960044559991	7.88018198528932	9.96098727886154
C	1.41317952210681	6.06083353841868	11.30910766341775
C	0.60134427468223	6.34205947446760	12.40689563879734
H	0.66163448152139	7.31511463611770	12.87975570859007
C	-0.27737238586208	5.38511392214055	12.88804820982192
H	-0.90154400442006	5.61878439589392	13.74328635686065
C	-0.34985486862102	4.13190182330032	12.29449590743320
H	-1.03377536348848	3.38331325143132	12.67668198197811
C	0.46336656031180	3.84866398927515	11.20491645905585
H	0.41187481479364	2.87740781018230	10.72569040395070

C	1.33329615303766	4.80539663119710	10.70798988001532
H	1.95665615217140	4.58364029392907	9.84983901743462

Table S15. Cartesian coordinates of the S_1 geometry of **2**.

P	11.96428020003380	4.74235592494936	8.01633192945332
P	11.41069325838086	1.96300338199797	2.24405677099294
O	12.57451674458882	3.76517865192653	9.00047308536335
O	10.53559289900769	2.80228221450660	1.34200940382120
N	2.22867751675258	7.01497465526856	10.72059289597871
C	11.85823051459386	3.99407491301470	6.35799849548283
C	11.54732666247224	4.73124893174562	5.20511173213619
H	11.41802807591874	5.81194263706389	5.25842497795926
C	11.41222843184293	4.08690132701582	3.98085857885904
H	11.16180149595348	4.65069406436843	3.08294021248445
C	11.59122335663614	2.69912156284017	3.89122407866153
C	11.89122781286651	1.95940162740081	5.04257822125572
H	12.01467148355938	0.87794409216681	4.98824269183522
C	12.02880927239915	2.60669662607540	6.26741241646762
H	12.27149223995485	2.04739686000087	7.17018263392578
C	12.94843307931141	6.25608895771393	7.76838418852414
C	12.65904604360027	7.41858929630835	8.49232657758145
H	11.78959367811195	7.44353785473910	9.14907654156576
C	13.47437768047642	8.54360720338433	8.36824914618261
H	13.24042654274516	9.44732573925099	8.92955904258772
C	14.58543435820041	8.51106295800154	7.52425377633264
H	15.21982106840759	9.39112026283418	7.42462931719972
C	14.88423687760052	7.35102909359345	6.80612321100565
H	15.75191579031430	7.32362717130993	6.14810423595525
C	14.06974833345392	6.22671080796225	6.92823555356091
H	14.30385881704885	5.32404377493812	6.36347308551210

C	10.74086873286321	0.29214646966377	2.49093041565438
C	9.35322385645623	0.14747405450195	2.37103645528165
H	8.74567480507732	1.01656124092795	2.12099298857067
C	8.76488656153720	-1.10208434912798	2.55782148441451
H	7.68549669776078	-1.21296710623546	2.46244684612448
C	9.55945184480492	-2.20978079868866	2.86037717489875
H	9.09903360623557	-3.18649687584409	3.00417237566260
C	10.94394410511869	-2.06966368693128	2.97028315457318
H	11.56502215113284	-2.93533755278833	3.19627220686611
C	11.53732855765071	-0.82144310607844	2.78192315580376
H	12.62013605826208	-0.72075428616171	2.85241421857407
C	13.08601258711541	1.75466238439487	1.57204204233535
C	14.21486748185279	1.53507752315123	2.36995768222402
H	14.12437141073374	1.49966387027832	3.45531765382981
C	15.46579095558984	1.37405808302393	1.77412645940693
H	16.34334307019501	1.20400575028044	2.39636058936982
C	15.59325590060936	1.44007036700380	0.38556436846006
H	16.57199286260186	1.31783637667367	-0.07679805446861
C	14.47000295089062	1.67193915113758	-0.41081422134966
H	14.57052189671930	1.73371846940111	-1.49368019466803
C	13.21777401205394	1.83124184921561	0.17998519035185
H	12.33615534807164	2.02672665812406	-0.42950688472805
C	10.31371204433523	5.23947943546655	8.47299332069505
C	9.56701366311886	6.23249748605039	7.79064832797763
H	10.02212152064038	6.79877412681282	6.97787057610644
C	8.26517282802285	6.51228542326919	8.14803025464077
H	7.74477905648250	7.29088547276289	7.59482826568192
C	7.60351557154808	5.82710321162481	9.21539743256733
C	8.37646919726829	4.82682472546076	9.88727801451590
H	7.94401312843108	4.26384811555311	10.71103793512130

C	9.67704136262316	4.54332272701785	9.52605632583664
H	10.23855418955563	3.77578519092979	10.05820360177799
C	6.24490972402872	6.12890693066689	9.59422276236446
C	5.45534455648202	7.09558042108536	8.89408996876640
H	5.86993654515911	7.61749040496466	8.03579655897648
C	4.15775840019943	7.38520943906288	9.25526570590172
H	3.57905627518016	8.10995894643745	8.68384956312115
C	3.55711945239526	6.72409742820530	10.34943124946945
C	4.30687950510842	5.76489252616144	11.06424581749602
H	3.85936786505379	5.26765130993803	11.92401361874990
C	5.60354453605189	5.47897305125159	10.69653956966675
H	6.14665692544896	4.74478601684440	11.28544428625742
C	1.83197700261286	8.34477383974298	10.84480985682480
C	2.76057606011585	9.31275280431723	11.28212201971403
H	3.76434674435488	9.00045695857659	11.56190244048714
C	2.37337528702332	10.64085313967394	11.38457660347724
H	3.08780802464966	11.38064114834513	11.74160086449861
C	1.07345433602530	11.02885223900088	11.04277129474981
H	0.77822956292352	12.07387975000645	11.11888497983216
C	0.15575773294082	10.07211564222208	10.59354957551960
H	-0.84963354557131	10.37350828732881	10.30446745918621
C	0.52405779469600	8.73920581882760	10.49037406455124
H	-0.17527348903076	8.00037892589505	10.10461588541741
C	1.34411823617714	5.96482163858208	10.95867555438377
C	0.35802077437794	6.07117509507794	11.96269624756830
H	0.29595104407340	6.97667488841075	12.56266149000029
C	-0.49245808156696	5.00354985572434	12.20744037682046
H	-1.24056459910383	5.08481984974455	12.99417052821538
C	-0.37706272048732	3.82064514479529	11.46804731552748
H	-1.04596072427521	2.98539493851132	11.66788768149984

C	0.60604354526958	3.71052649371589	10.47874262934777
H	0.69428074958732	2.79503883333888	9.89627875455245
C	1.46906207660204	4.76634841929214	10.22467638472654
H	2.22559515390297	4.69415682300173	9.44633892953275

Table S16. Cartesian coordinates of the T_1 geometry of **2**.

P	11.77665079261252	4.34801830892728	8.03317584390911
P	11.53331303750462	2.15144075522198	1.99055497686142
O	12.31992829766829	3.28847935908994	8.96589890142836
O	10.68906417380730	3.05667885710517	1.12528511999679
N	2.22670098960314	7.11173791926441	10.87270019977123
C	11.73544040403882	3.76346590756361	6.30537303052918
C	11.73886282137169	4.62765951870104	5.20352044157750
H	11.80275503783800	5.70611644525759	5.34767267558139
C	11.66808971361581	4.10970766695185	3.91351053942470
H	11.65162606479915	4.77905580659490	3.05390978581727
C	11.61554128003764	2.72405110473234	3.71527453527469
C	11.62134860482347	1.86015483855360	4.81536836739084
H	11.57162854286434	0.78176890877531	4.66963667794082
C	11.68334987575541	2.38012321909491	6.10687647952252
H	11.69610659129427	1.71701402740335	6.97109041816840
C	12.82833738786799	5.83291603898476	8.00538639207468
C	12.32686606290851	7.12700390624910	7.82741404741294
H	11.25609003630577	7.29066739655629	7.71156530037480
C	13.20030454529711	8.21517290373581	7.81128363891574
H	12.80785075933417	9.22174578553588	7.67299294673475
C	14.57093106834203	8.01489709353245	7.98098842442165
H	15.25030171713221	8.86643763505782	7.97133389030625
C	15.07195849906780	6.72515750091071	8.17216189580603
H	16.14055336991446	6.56916204366071	8.31487158924926

C	14.20340016263616	5.63561458890624	8.18685707736244
H	14.58276298213256	4.62710582610428	8.34927483315054
C	10.89771285735562	0.45219140177856	2.03043213455828
C	9.53673274099916	0.28385535943846	1.74880170571208
H	8.93141205299279	1.15640513394289	1.50586435174909
C	8.97556962930415	-0.99193528555650	1.76601091894019
H	7.91703879663639	-1.12165105094094	1.54436539303630
C	9.77159482678430	-2.10042901846146	2.06027015345100
H	9.33289607766245	-3.09743633790628	2.07070269195893
C	11.13117184870637	-1.93528876431391	2.33199365038757
H	11.75394370632577	-2.80128595695777	2.55170065928363
C	11.69764108764029	-0.66113622550832	2.31431894524152
H	12.76226283906214	-0.53922275463924	2.51216690250040
C	13.25444964161176	2.05899605982985	1.42173892657101
C	14.32584391739085	1.77128182227237	2.27562382984145
H	14.15355245169729	1.60828824395496	3.33951160223672
C	15.62182433348590	1.70675350857208	1.76429770234995
H	16.45546109481400	1.48367190298391	2.42869739339643
C	15.85006148748137	1.93729464123775	0.40640851073449
H	16.86387401300605	1.89067065359354	0.01062394550824
C	14.78346223215436	2.23737593311123	-0.44338321929269
H	14.96332500192284	2.42767174540129	-1.50071019138962
C	13.48617548929395	2.30032836105588	0.06230834222763
H	12.64671301130439	2.54780873464170	-0.58656595033031
C	10.10097421798078	4.84288756235640	8.43929633610488
C	9.23572662830477	5.53010528220821	7.55103175014661
H	9.56352115028634	5.76597463023045	6.53882578010022
C	7.96832492229222	5.89588621716822	7.94054916868238
H	7.34185038930183	6.41133247778695	7.21734585697848
C	7.45453364604006	5.59027690492937	9.25037880672313

C	8.34453467709370	4.86341492272873	10.11873568477799
H	8.02357018848948	4.58823450419975	11.11989500290517
C	9.61503866484214	4.51014670475226	9.72642495137766
H	10.26502644371204	3.96010566114279	10.40613029487902
C	6.15045343562666	5.97511724023306	9.65713110214263
C	5.27918306948093	6.77326365180434	8.81486588788373
H	5.61806511827108	7.08699695784572	7.83151262368724
C	4.02682147143348	7.14593024253615	9.20989618987036
H	3.39845117197911	7.72996649358049	8.53932618215795
C	3.50353569703030	6.74502881747844	10.47921450445894
C	4.33817950614229	5.95465402001586	11.32947794854792
H	3.96842162402962	5.66459311207744	12.31174839768729
C	5.59847923204421	5.59754187721473	10.94525441922221
H	6.19893553679769	5.01786866827022	11.64071343633246
C	1.71790985576042	8.37831221935762	10.52556669526609
C	2.55534618934973	9.50426001485497	10.57187016625430
H	3.58467969712394	9.39037845853926	10.90751198835112
C	2.05922391525709	10.75297196809639	10.21124975912063
H	2.71297649338092	11.62271130206921	10.25862168014023
C	0.72955355297516	10.89551262138617	9.80779303971950
H	0.34395413390613	11.87477963169348	9.52874279700117
C	-0.10422361486697	9.77408561852618	9.76524479222528
H	-1.13960359093848	9.87548369285578	9.44311242687405
C	0.38189989626303	8.51984227122782	10.11719794715242
H	-0.26092003891876	7.64283268606194	10.06694579740356
C	1.43399261922023	6.22012952136255	11.62041202378652
C	0.63569875977829	6.69230482496916	12.67491491136435
H	0.63542224453687	7.75383616036550	12.91557071685909
C	-0.13440051448941	5.80016870881908	13.41261340302922
H	-0.74598199696228	6.17280489524411	14.23307627715878

C	-0.11444908761270	4.43369879370956	13.11766639378593
H	-0.71748087019421	3.73915711313443	13.70029540745004
C	0.68284228275457	3.96463472676502	12.07101780932827
H	0.69618574916853	2.90344198059416	11.82656700650143
C	1.45243180229205	4.84830041110688	11.32126233151472
H	2.05834877482824	4.49100296069797	10.49044787736868

Table S17. Cartesian coordinates of the ground state geometry of **3**.

P	11.88433462916901	4.54387297156965	8.12442210724526
P	11.84222209612653	2.94731748231771	1.93061955428348
O	12.43148631792067	3.44303615272778	8.96645854520106
O	11.13351458799057	3.96525924940136	1.10474667955195
N	2.29164497735849	7.03943353899505	10.87006000230913
C	11.88322903194746	4.12312458342259	6.36561579291634
C	11.91521567411947	5.09287472194903	5.36611419984803
H	11.96115463312466	6.14440643932477	5.62528182691351
C	11.89121905994358	4.71693190339655	4.03429545173725
H	11.90897106634905	5.47111337576168	3.25586575334480
C	11.83954240911029	3.36786081592901	3.68979099624005
C	11.80900370371060	2.39921453316932	4.68841536722961
H	11.76276367648659	1.34803426177535	4.42809569313405
C	11.83256250766148	2.77564918216069	6.02194185330296
H	11.81474081182755	2.02345245620543	6.80195806702786
C	12.83359591144561	6.07015780297349	8.26566405722098
C	12.24741564250554	7.32823768710712	8.15171752922095
H	11.17759922948350	7.41930224510356	8.00096718863874
C	13.03158679924004	8.46915337194412	8.23931138799882
H	12.57178758981887	9.44681665717977	8.15277759969371
C	14.39980983035779	8.35771866549631	8.44374101149319
H	15.01052402792334	9.25055753609803	8.51523810990594

C	14.98735968231435	7.10464872537861	8.56252137573962
H	16.05509157714808	7.01781682946379	8.72756113047307
C	14.20771482473233	5.96253995608027	8.47348473624807
H	14.66008769151224	4.98219636479573	8.57257602827248
C	11.10075269381864	1.30676347038977	1.82941500402299
C	9.71906837269675	1.21231233094040	2.00145349112713
H	9.13447533062540	2.10718011882687	2.18634119495945
C	9.09368860696692	-0.02120098826422	1.93416186595264
H	8.02046525429827	-0.09044532070720	2.06893878851839
C	9.84211980098136	-1.16729620981494	1.69327940506276
H	9.35121586246606	-2.13240579447368	1.64035834960502
C	11.21500559528030	-1.07770683566890	1.51818130935222
H	11.79901845784844	-1.97039808589892	1.32646652253328
C	11.84561092145756	0.15701415477102	1.58613548745071
H	12.91820108585869	0.22251311312118	1.44700454374655
C	13.57678246706183	2.76133130470347	1.47539125268307
C	14.54657439104626	2.33970889492317	2.38199575710381
H	14.27848887546688	2.12985516818863	3.41125454867649
C	15.86216729053859	2.19109378443306	1.97084557964402
H	16.61452686676036	1.86487735914324	2.67951827566045
C	16.21461156301621	2.46534782224532	0.65613549613572
H	17.24444763872158	2.35146365242413	0.33722278873071
C	15.25180618913673	2.89105614535736	-0.24908204647344
H	15.52747641504573	3.11035380416758	-1.27413598778177
C	13.93485609573469	3.03977647325797	0.15801810144399
H	13.17955747122361	3.37911940846899	-0.54161816193396
C	10.18504698631433	4.98057315248508	8.53401740624426
C	9.17282169165457	5.08085025105495	7.58494571463296
H	9.38172269918743	4.88674458969798	6.53970782602258
C	7.88555591109033	5.41847427139423	7.96838099597241

H	7.10593797270258	5.47090937679605	7.21749294419984
C	7.57654082439887	5.66275264841072	9.30634727459831
C	8.59841642530969	5.55551321599309	10.25408731099842
H	8.38715258213520	5.75723967950754	11.29766903830294
C	9.88383428650942	5.21990314890283	9.87531511567438
H	10.66246427234337	5.14782880526661	10.62704921712268
C	6.20622110271760	6.02165419961107	9.71251656317796
C	5.39246246125993	6.79625429881070	8.88450384370608
H	5.77424142643493	7.15359952705849	7.93530717189661
C	4.10311470169534	7.12870179245843	9.25870653975007
H	3.47869474430979	7.71998695324826	8.59988209524558
C	3.60245219742678	6.70026426262276	10.48268918746242
C	4.40256479873438	5.93369004781889	11.32219249224787
H	4.01588575923753	5.60534791549268	12.27939959340194
C	5.68661129442772	5.59527841642748	10.93549044562266
H	6.29025301224017	4.98112907811802	11.59331367073985
C	1.76911083674057	8.32248579469078	10.91987284264627
C	2.38607003593941	9.53841775239914	10.64985544316815
H	3.42008245761529	9.58978731409210	10.33275076805370
C	1.63092111836942	10.68909683170199	10.80493787572556
H	2.08805933574249	11.65035801026306	10.59938233615960
C	0.29678359776234	10.63841779773752	11.22155828179619
H	-0.26370877760940	11.55958061805809	11.32973500019508
C	-0.30899950442057	9.42590728847665	11.50123101949429
H	-1.34037067219455	9.38673248870304	11.83397695816126
C	0.42847769349153	8.25456040781700	11.35383949533790
C	1.30867900424443	6.14462257866884	11.26350354980106
C	0.13320637231850	6.86132478043379	11.57126941075152
C	-1.00720202824621	6.17415949475813	11.97747215363480
H	-1.91672719591914	6.71397041567943	12.21689062767854

C	-0.96165986760767	4.79374171670692	12.06506905551886
H	-1.84025637975818	4.24365698828276	12.38106226446669
C	0.20789126516182	4.09750872587624	11.74316795809410
H	0.21865542456334	3.01556815092001	11.81051741724683
C	1.35593420387784	4.75748429207290	11.33722992615500
H	2.25387707341719	4.20793407222304	11.08414941248114

Table S18. Cartesian coordinates of the S_1 geometry of **3**.

P	12.01573369514180	4.66961954398909	8.22640619299145
P	11.82043880745035	3.02479280160089	2.01067547042480
O	12.57939967762776	3.55293852810346	9.08037296292002
O	11.16840340543044	4.12357149731895	1.20404938831001
N	2.25342311043243	6.94833012321026	10.81918801626936
C	12.02607230139745	4.23559761340225	6.45345667422714
C	12.03946247173654	5.19496462703226	5.43374635489263
H	12.10748199932035	6.25560226772337	5.67498655932238
C	11.96995380665205	4.79682068706653	4.10130948743355
H	11.95762606402092	5.53802575331125	3.30294051779268
C	11.90660616696480	3.43649643750233	3.77843947511541
C	11.90538178218765	2.47506884583843	4.79667894597954
H	11.84535924528505	1.41434270782161	4.55463383159737
C	11.96861117200921	2.87554771463996	6.12896264639476
H	11.97149050020954	2.13681930894721	6.92982579924734
C	13.04298737006284	6.17268533347784	8.33407763121955
C	12.51152051438020	7.46251832487248	8.22566981446173
H	11.43906367077794	7.60096371835059	8.09012900643445
C	13.35358175418031	8.57261357770114	8.30362416985393
H	12.93729814239001	9.57552060608742	8.21727230685325
C	14.72432176905083	8.39765833074129	8.50032510201711
H	15.37984643537932	9.26544502538301	8.56448497312688

C	15.25530251588766	7.11160597863012	8.62366127747419
H	16.32366084500666	6.97510121650136	8.78744646596848
C	14.41725477603689	6.00073587887996	8.54223130666277
H	14.81971672278414	4.99368975357414	8.64836419891472
C	10.92289679219891	1.45065488643938	1.89300265107045
C	9.54917018812859	1.52422996314549	1.63247495705162
H	9.08390239468081	2.49906367546112	1.49047403444848
C	8.79456623523711	0.35570001152992	1.54465253098723
H	7.72619671507811	0.41404172786086	1.34067331044915
C	9.40933415840892	-0.88669973301946	1.71285894461133
H	8.81900753614721	-1.79953244327096	1.64263102982250
C	10.78075933560547	-0.96228221215800	1.96216816633256
H	11.26204098487411	-1.93176917111235	2.08326757699279
C	11.54053958604610	0.20417133293057	2.04856540056893
H	12.61347908621524	0.13983763432298	2.22785048368120
C	13.52382675139282	2.68716266165304	1.47980537631792
C	14.50769678523597	2.18384049506916	2.33894291872414
H	14.27986191013414	1.99593614991930	3.38789363935036
C	15.79070548612412	1.93264322667873	1.85279542091787
H	16.55588202959656	1.54165321802896	2.52196917663620
C	16.09468613327786	2.19051084598282	0.51501377767020
H	17.09853198543978	1.99665903420191	0.13900563437010
C	15.11750421627209	2.70465117884065	-0.33975643284420
H	15.35745175768367	2.91475737194403	-1.38125370490577
C	13.83350621394104	2.95558443290921	0.14091782682315
H	13.06624653673131	3.36930751891591	-0.51264944919815
C	10.32635200518688	5.09115014665157	8.61067362967170
C	9.45421574227474	5.75973456901012	7.71661005716075
H	9.79830904293911	6.03555486942148	6.71986146306418
C	8.15695445933424	6.05714220958916	8.07418896003918

H	7.53350754382636	6.56167193045870	7.33985727488103
C	7.61885442785142	5.70973622514090	9.35676334322529
C	8.51552817380085	5.01775063879108	10.23674921713825
H	8.17962169120347	4.70953287096615	11.22417933635089
C	9.81240278931070	4.71981738302153	9.87632207650060
H	10.45891123292933	4.18297958501749	10.57037010380916
C	6.26832469512336	6.02506574406002	9.72794806462205
C	5.37624037295516	6.72179719528211	8.84538428753305
H	5.71265132964955	7.01660648182605	7.85500142377871
C	4.08103949037306	7.02559409973144	9.19564267618537
H	3.42624304562970	7.52515363798479	8.48283498112637
C	3.57543633951805	6.64529549147183	10.46124676294140
C	4.42368292386694	5.95540091423338	11.35919128559239
H	4.05679562791051	5.69216840220272	12.35044122187264
C	5.72070567872031	5.66318596130636	11.00445760868462
H	6.34104069993910	5.15127540767283	11.73554447593813
C	1.66283769301721	8.19851594265431	10.73496272189741
C	2.24026199838130	9.41091122559935	10.34046960330784
H	3.28470223532058	9.47045376441290	10.04419270692067
C	1.42409184369571	10.54104600222479	10.35551097042843
H	1.83760237951378	11.50042268939157	10.05067503751121
C	0.08735201006876	10.46269027385786	10.76652873393239
H	-0.52447934035004	11.36298945468008	10.76967805715426
C	-0.47581143154494	9.24706749037531	11.18978685233700
H	-1.50986309115577	9.20471910678032	11.52756622417205
C	0.31609342336083	8.10958188794848	11.17622332733234
C	1.32470273439445	6.04551825501247	11.31046098889816
C	0.10120754586600	6.72229652749375	11.55807712440777
C	-0.98551916121166	6.01398232075220	12.04647115721256
H	-1.93521621139787	6.50984726763510	12.23972387288694

C	-0.83977570202153	4.63589809625156	12.27792392566137
H	-1.68470186722518	4.06821877071465	12.66345870643700
C	0.36586938523613	3.97617662822234	12.00770164729062
H	0.44259536382496	2.90438553877574	12.17985178778677
C	1.46923208486654	4.66781375613009	11.50971852946390
H	2.40012363376034	4.15670566026457	11.27649778405824

Table S19. Cartesian coordinates of the T_1 geometry of **3**.

P	11.94319224637282	4.45665542093126	8.21116373448781
P	11.87324367316985	3.10282298284186	1.92673468200955
O	12.45949158328100	3.29529543482037	9.02995686762292
O	11.21726073859545	4.23183258272081	1.16746099904575
N	2.28324299747436	6.99858808297470	10.86633153602757
C	11.97684311960213	4.10714286160401	6.42314788147940
C	12.03925765431281	5.11157620435188	5.45041120951664
H	12.11473111997295	6.15908987201060	5.74062042550992
C	12.00317394914789	4.77414544920518	4.10050707683202
H	12.02684957682953	5.55096411098157	3.33722592826346
C	11.92465163244529	3.43066811087109	3.71570103509623
C	11.87595131326183	2.42541162111193	4.68730218386913
H	11.80371500006382	1.37806791066774	4.39596441791090
C	11.90567320761799	2.76425722778113	6.03843339535128
H	11.87231419790888	1.98995428763045	6.80396262447657
C	12.94482571506449	5.95684228363351	8.43773859333967
C	12.42128669914455	7.24572600016969	8.28156231920459
H	11.36804104611460	7.38512162162908	8.03805993585429
C	13.24814334142436	8.35638800730650	8.45006976682681
H	12.84005567979975	9.35881247042221	8.32804127194778
C	14.59271702776465	8.18250759744865	8.78277580816340
H	15.23586072686220	9.05140936599292	8.91714956803006

C	15.11270641388893	6.89760033349548	8.95269400725325
H	16.15955965134678	6.76293474001978	9.22197896613868
C	14.29083465726896	5.78470705082533	8.78253460454684
H	14.68163625022048	4.77758961201761	8.92407087288528
C	10.99966198447360	1.52582809439949	1.71817363965660
C	9.63424993779132	1.59636248716720	1.41635544181852
H	9.16290488450131	2.57158280043298	1.29964672687316
C	8.89665342100900	0.42486122768683	1.25496030459958
H	7.83497022632661	0.48084559404094	1.01816132951769
C	9.52016048108579	-0.81697640653767	1.39129808122596
H	8.94327805276100	-1.73204317516079	1.26300660624537
C	10.88351123619022	-0.88893850196333	1.68289703731017
H	11.37182478425569	-1.85764980861922	1.77919351690230
C	11.62647051922788	0.28039531066205	1.84295903656034
H	12.69369410182249	0.21915848969775	2.05438888050404
C	13.59238938226320	2.82465609621455	1.41558543924720
C	14.56200539045799	2.27195969481654	2.26069582756866
H	14.31103084574977	2.00281496013586	3.28651965605702
C	15.86074137745175	2.07681972930166	1.79123458880359
H	16.61515154068615	1.64775412384320	2.44917234888132
C	16.19431207862025	2.43952186327316	0.48509815879921
H	17.21053329490882	2.28953245738116	0.12258063413465
C	15.23098960122230	3.00262025936890	-0.35433013916040
H	15.49388801334355	3.29459285376380	-1.37023763234698
C	13.93118254849616	3.19796082339960	0.10941617627019
H	13.17397438004445	3.64937173222980	-0.53076131779827
C	10.23945174017201	4.89098864978280	8.59109709242943
C	9.34343088790131	5.46861130531096	7.65524223529553
H	9.66529144428710	5.64410932034063	6.62949408331198
C	8.06215692011427	5.80378281288364	8.01736672179347

H	7.41500936957843	6.24027445522149	7.26182444314878
C	7.56627501193390	5.58784779284293	9.35858781619531
C	8.49687345324877	4.97825708882311	10.28358982265004
H	8.18804903688773	4.76639085233982	11.30359414588785
C	9.77364391642416	4.64161652965642	9.90915830027777
H	10.44493710197004	4.16977444975274	10.62595913585539
C	6.25408324426666	5.94467987147450	9.73654712846090
C	5.30450755505268	6.51724335126967	8.79493993178746
H	5.58952311411624	6.65348043125695	7.75604153124286
C	4.03534902350610	6.86143465600499	9.16218255459482
H	3.34310006858115	7.24554367638657	8.41544232813832
C	3.57780693936432	6.65881109090681	10.49910074018376
C	4.48363737220718	6.09746378488763	11.44916284136281
H	4.16573216203360	5.98540217332558	12.48393286526689
C	5.75868882253681	5.76362453672325	11.09214983668188
H	6.42154547431221	5.38046487300624	11.86244924245716
C	1.61477124841397	8.17550469506560	10.51831725311262
C	2.08419012523212	9.28571912764022	9.81146673623656
H	3.09925511677696	9.32937081237811	9.42427052047878
C	1.20787899561474	10.35379646764284	9.62946284735203
H	1.54846694436813	11.22812431586872	9.07674903165108
C	-0.09504049378352	10.32967556890692	10.15110256400541
H	-0.75343655448692	11.18201669914657	9.99117257565477
C	-0.54622212537470	9.23423198755976	10.88569241843745
H	-1.54816191222336	9.22471549902020	11.31285258697536
C	0.31346533258108	8.15089999278891	11.07565073786804
C	1.42269229673142	6.21890939587982	11.64401347839529
C	0.19184484963477	6.90144354532583	11.79819111071607
C	-0.84673399238750	6.31015519529771	12.51947332384880
H	-1.79794664847383	6.82684794225464	12.64057699477685

C	-0.64781566559527	5.04474811259168	13.06927143538323
H	-1.44659440604140	4.56988296851800	13.63671412904331
C	0.56701227944000	4.36651269066024	12.88335313207457
H	0.69448024836013	3.36950026223734	13.30247963747115
C	1.61508977678977	4.93589491389228	12.16289118884824
H	2.54078900327879	4.38815858712559	12.00383130228467

Table S20. Cartesian coordinates of the ground state geometry of **4**.

P	12.34264304151731	4.92798296872374	8.20767078130230
P	11.67458272467019	2.91369092599229	2.20368364407990
C	12.18263635292979	4.36323630853941	6.50359868227003
C	12.61916109589177	5.15497590169905	5.44621576697287
H	13.08340122362639	6.11533595795122	5.63168180205217
C	12.45772442299606	4.71490508506203	4.14390024729978
H	12.80882225521703	5.33701017841788	3.32919648452410
C	11.85594696544262	3.48329980954285	3.90021356053091
C	11.41913888298740	2.69059024308407	4.95784555330305
H	10.95139147179433	1.73196846190003	4.77141296517701
C	11.58001803621027	3.13185257159566	6.25918070054797
H	11.22437960539591	2.51358527130664	7.07502796773105
C	13.34691285618658	6.40791553715451	8.22892366096853
C	12.76868348908608	7.63241853410602	8.55195643356314
H	11.71553375553716	7.69037191243588	8.79951455078369
C	13.55033294133084	8.77755313893403	8.56033285024373
H	13.10255685699823	9.73094323704179	8.81365415167740
C	14.89933104053527	8.70006767445493	8.24765017452987
H	15.50786923924009	9.59684914668147	8.25619655089981
C	15.47576969351335	7.47759220131372	7.92455867527775
H	16.52978844689674	7.41941718120165	7.68095682704185

C	14.70468218279768	6.32856519798806	7.91406741390230
H	15.16004080422775	5.37956350922877	7.65397654933697
C	10.59017912281103	1.49525176147326	2.17459835186092
C	9.22708874310432	1.67088768601568	2.41924616884145
H	8.82035655985068	2.65584208157875	2.61834768538996
C	8.38731251998124	0.57119081824549	2.41567534319260
H	7.32872342738111	0.70358231396054	2.60384759502343
C	8.90086208200408	-0.69638548311133	2.17011825119035
H	8.23844077405040	-1.55411692889085	2.16642202228589
C	10.25573892409705	-0.86909896051340	1.92856940008038
H	10.65446528431521	-1.85778795558168	1.73626366644980
C	11.10646499991703	0.22529890691958	1.93028090567206
H	12.16426772889824	0.09031927348492	1.73964071874507
C	13.28635321679257	2.47144206853243	1.57677722681835
C	14.31198922262576	2.14177875832174	2.46079729515623
H	14.15052311663597	2.18303044144683	3.53132787974072
C	15.54714009796368	1.76017479668892	1.96269239049556
H	16.34656690162612	1.50518146740247	2.64781462161144
C	15.75764865467096	1.70790880474297	0.59217730949421
H	16.72595619037903	1.41169780786203	0.20598392752714
C	14.73415024121215	2.03461790261037	-0.28782523334976
H	14.90069755873519	1.99364322683116	-1.35743334683396
C	13.49546347830354	2.41580182276266	0.19858587903085
H	12.70110545961198	2.66340976835742	-0.49536126936965
C	10.71893907609058	5.23771020891329	8.83486595544924
C	9.70830837094264	5.68187728604041	7.97684341840625
H	9.89967063365732	5.79258815201278	6.91549879766154
C	8.45234055064339	5.96680849615506	8.45944991939543
H	7.68086593777698	6.29548928445859	7.77600638496762
C	8.15681375148445	5.81451085903477	9.82447435519367

C	9.17718275186961	5.36998154518899	10.68162494109639
H	8.97875779484191	5.25464122735936	11.73875831977520
C	10.43361635617541	5.09053190954694	10.19545967182967
H	11.19907640092030	4.76583773310763	10.89017707699998
N	6.89761104966766	6.09136270463945	10.30798098345783
C	6.42460905287565	5.51030657159110	11.51187795194259
C	6.52398316003862	4.13808232877715	11.71930329697248
C	5.82699070073723	6.31465460840303	12.47636695022353
C	6.04345707196535	3.58011203545247	12.89301668303545
C	5.33553647126765	5.74772803464112	13.64165017060059
C	5.44572547372856	4.38077781833242	13.85737175217972
H	6.97473516485478	3.51221639944015	10.95794276968710
H	5.74587933022598	7.38197140042149	12.30684389220566
H	6.12305697431898	2.51011271284320	13.04720645176969
H	4.87117680123559	6.38104991293411	14.38872529040837
H	5.06382521100353	3.94041385196756	14.77085592989420
C	6.02270751920899	6.96647684033852	9.61483327791768
C	4.71512490988658	6.57106625933462	9.35472022744690
C	6.45564787159676	8.22910406475712	9.22288237638933
C	3.84837816378114	7.43463347987482	8.70354554195896
C	5.58797818768219	9.08211940225130	8.55993801398240
C	4.28142249757784	8.69031447793334	8.29961514154900
H	4.38127996209492	5.58881424672316	9.66847771032960
H	7.47053921107863	8.53974407177195	9.44263339717837
H	2.83030950071949	7.11995291437053	8.50496200109644
H	5.93138743038252	10.06542140669652	8.25960376544750
H	3.60244884743908	9.36270465972457	7.78862778426599
C	10.96639046386326	4.23031382089661	1.22363321142763
H	11.64230147146356	5.08562599884927	1.21639057328671
H	10.81254629387734	3.87088861642161	0.20572849135861

H	10.00853117982752	4.51777835398993	1.65798722804391
C	13.15856856276710	3.65527392686583	9.16424764307684
H	12.54299647250288	2.75552557302767	9.17890909972370
H	14.12470834377624	3.43605096375509	8.70907435813368
H	13.31095505915316	4.01911146465968	10.18093091316383

Table S21. Cartesian coordinates of the S_1 geometry of **4**.

P	12.21485385901349	4.65633062244272	8.24170892265790
P	11.59647863214518	2.92862390652285	2.18414969166354
C	12.12115118737792	4.15462826604615	6.57843045096974
C	12.18970026106395	5.12941044672588	5.52524236599118
H	12.40663437633140	6.16959212363416	5.76487024911598
C	11.99852818201043	4.76924946502434	4.21736874112845
H	12.07950597731018	5.53284664285832	3.44521156480641
C	11.70431788272789	3.40901455333295	3.86216542924711
C	11.65026696263834	2.43418527151096	4.91093061920792
H	11.45013984303121	1.39168959110461	4.66845462331525
C	11.83577795128766	2.79108174501543	6.22104902113104
H	11.76845567051115	2.02324946848641	6.98945784781772
C	13.30913163992051	6.08578754271783	8.40109172807037
C	12.82117230066369	7.36080765391750	8.70653843580554
H	11.75975852903233	7.51854080699890	8.88961811431137
C	13.70252013868375	8.44031147799496	8.76899743519769
H	13.32263563494175	9.43335208336941	9.00362848684560
C	15.06266657967503	8.24762138844043	8.52980948559763
H	15.74834120958807	9.09222431925702	8.58135818518092
C	15.54849783298640	6.97502892676894	8.21936729297082
H	16.60996220808568	6.82559100201214	8.02839216201260
C	14.67498034213403	5.89346396638041	8.14863822456849
H	15.05524219903210	4.90541017962054	7.88979366393229

C	10.72734964270158	1.34738542907790	2.07290464908659
C	9.36929438789089	1.30228605184586	2.41853397824155
H	8.84750905288001	2.20752104642021	2.72837073233167
C	8.68597712059102	0.08994128768532	2.37574131553908
H	7.63036306686515	0.05454464904416	2.64038820372713
C	9.35447341215061	-1.07694759587458	1.99711017638186
H	8.81747815801334	-2.02400057578232	1.96578509421172
C	10.70720439701905	-1.03284120987676	1.66139659869694
H	11.22967958200957	-1.94263894112330	1.37022615765361
C	11.39806030142077	0.17862148564819	1.69791356106975
H	12.45468518337381	0.21067584536838	1.43709279572193
C	13.22271969335545	2.72013856557026	1.40351324591923
C	14.36147853789373	2.73442619485433	2.21567260351420
H	14.25686917327681	2.88207314571698	3.29035424703483
C	15.62246658716004	2.56463554122239	1.64387810837849
H	16.50939009167120	2.57775855638161	2.27547392331865
C	15.74467308162138	2.38063569313954	0.26676955491119
H	16.72976923882445	2.24907899102178	-0.17882605211109
C	14.60713809949271	2.36464743261098	-0.54447385591029
H	14.70406782926274	2.21896584959675	-1.61924924868207
C	13.34500339978661	2.53399952080488	0.01929970983235
H	12.46272303542528	2.51086462247624	-0.61985366317533
C	10.59366258100866	5.16505065011493	8.92110711725977
C	9.50262205903749	5.22654641318684	8.04889624427927
H	9.63223994778235	4.96828664428287	6.99877136591370
C	8.25059554243865	5.59631205113570	8.52150183209412
H	7.39110032741817	5.61863910808463	7.85467768164650
C	8.09091053378326	5.89823324008977	9.88171907756636
C	9.18241346405260	5.83546451081443	10.76405361267588
H	9.05046887153181	6.10121588486012	11.81100633335047

C	10.42962808634605	5.47366175885991	10.27966797640248
H	11.27735892347487	5.45359409753060	10.96340126743863
N	6.82413702171840	6.26458314286984	10.36694143018974
C	6.38181857695097	5.76370678973832	11.59372679132971
C	6.70532116274590	4.44438020945445	11.96784294168413
C	5.61908140564720	6.58237414223834	12.44997684047282
C	6.25973591168098	3.95443964437604	13.18539829828204
C	5.18618148363761	6.07768163020480	13.66628359263524
C	5.50181779894937	4.76602084287049	14.03709891384674
H	7.26565280255289	3.80995712349783	11.28438143113497
H	5.41228699341569	7.61292477178096	12.16916242875474
H	6.48919454415981	2.92927468110593	13.46885033249487
H	4.61440032662419	6.71346975034202	14.33921329071800
H	5.15804771702936	4.37558570154394	14.99316560814252
C	6.01768464704127	7.12087549512351	9.61596882327396
C	4.62209457497307	6.92369710595476	9.58691482729681
C	6.60522939496629	8.17424776927599	8.88622388790341
C	3.83023275969441	7.77639101782983	8.83437099745136
C	5.79734336727250	9.02060931616215	8.14383209261643
C	4.41164480040378	8.82591872383671	8.11392860640749
H	4.18455386884988	6.08149267864139	10.11881956968698
H	7.67904690754090	8.33890757062704	8.94328496352681
H	2.75509223499728	7.61441531344558	8.79265288779549
H	6.24466159339283	9.84789102606095	7.59666082673393
H	3.78351491673837	9.49284857933539	7.52637567660560
C	10.70316406906682	4.16065784993253	1.20633285230262
H	11.24913369530103	5.10908072187339	1.23897868395083
H	10.62701540380870	3.81984338840128	0.16926594490886
H	9.70194906076473	4.29490121819800	1.62733206630105
C	12.79350204046155	3.31795887044804	9.30745595786536

H	12.07002248633789	2.49667253741892	9.28066455042547
H	13.76578002499133	2.96809767031863	8.94574833327018
H	12.88978732352935	3.68732427911992	10.33305396812511

Table S22. Cartesian coordinates of the T_1 geometry of **4**.

P	12.54480113303577	5.20688135228493	8.30672916298075
P	11.23695579852444	2.31222435858754	2.82049205573586
C	12.37674572657319	4.40806886007676	6.75053100122222
C	11.90140016563671	5.15851227715790	5.62750924708775
H	11.83775749758075	6.24455856522195	5.68035786517431
C	11.53990292857026	4.52750511999909	4.46106918703905
H	11.20944420783415	5.13281797329446	3.61815880757894
C	11.61032730740821	3.10972750946426	4.34818376638599
C	12.08810600137916	2.35993342069742	5.46105510514063
H	12.17373546461910	1.27676559589437	5.39010583484665
C	12.44882136398135	2.98699217684922	6.63105303210868
H	12.80501112910649	2.38114242724593	7.46170828535830
C	13.50924154166102	6.72662085346609	8.14205939179851
C	13.38218253464829	7.74056714927845	9.10031502033496
H	12.65838562008325	7.64680987165029	9.90984477558430
C	14.18536340610746	8.87518605312601	9.01214933289495
H	14.08716866353778	9.66638586691070	9.75370464103889
C	15.11211439166505	8.99661353402407	7.97451074236332
H	15.73625708858093	9.88659627924238	7.90670860838353
C	15.24218339164270	7.98231404238339	7.02468274707247
H	15.96612376714134	8.07809021249110	6.21703410208843
C	14.44427098750019	6.84243172039493	7.10696080200620
H	14.54349957316486	6.04664342136308	6.36947248202626
C	10.86805748393087	0.57375115570919	3.13836750069432
C	9.70239336974282	0.25327217350935	3.84898409195404

H	9.02397962021522	1.03759213564792	4.18369699565421
C	9.41541448533129	-1.07821952163311	4.13638950760675
H	8.50922587276954	-1.32949300372619	4.68514705878718
C	10.28893410527548	-2.08727877373791	3.72323891167009
H	10.06141221559001	-3.12793057241542	3.95017219368654
C	11.45137917371349	-1.76660372679606	3.02293285183235
H	12.13356985671600	-2.55281049975837	2.70386056162534
C	11.74580198221538	-0.43537822871318	2.72818185351338
H	12.65478188265278	-0.18665642393887	2.18295657152079
C	12.62358259709859	2.39777829682094	1.65986196914219
C	13.86256990177304	2.86148557164293	2.11496523680502
H	13.97710376606162	3.17818243551792	3.15112503371995
C	14.94386337883513	2.91872232196768	1.23605694902539
H	15.90825098412355	3.28107371194051	1.58852671655957
C	14.78788627400328	2.51361832323873	-0.08940961947863
H	15.63330148495255	2.55953461901449	-0.77451127501847
C	13.55073572795258	2.04883642939956	-0.54270635492352
H	13.43130553105030	1.73132405396686	-1.57735571725962
C	12.46564887213247	1.98892530278606	0.32778848877385
H	11.50695293894226	1.61576806195538	-0.03149364626581
C	10.92033696914114	5.67971949091356	8.94493995116374
C	10.23967216529397	6.75922022145788	8.33586790214197
H	10.75429650301583	7.40741493869970	7.62815528088396
C	8.91171033283321	7.00378468775205	8.62777850964466
H	8.38270652490816	7.82086416283805	8.14071177265382
C	8.22943729121285	6.17089591828593	9.53648579682781
C	8.90304084529694	5.09765368113056	10.15609733550732
H	8.38436659319579	4.48762826988779	10.89321550881882
C	10.23199885979851	4.85790795372696	9.86145607288106
H	10.73879410417419	4.03495247839198	10.36117110477797

N	6.87934442208791	6.40879339397068	9.82960518329283
C	5.99760028379903	5.33221509042293	9.94563118978472
C	6.15192915835296	4.20930472395206	9.10777907973194
C	4.96805466558803	5.36600847266087	10.90768912836487
C	5.27771710985951	3.14032283117445	9.23238913231162
C	4.10641382747850	4.28629780083633	11.02180052519858
C	4.25556101714071	3.17264927105449	10.18711397371552
H	6.92950020144542	4.20736054341323	8.34696149409256
H	4.88286058442622	6.21906179195481	11.57763357280330
H	5.38236898588729	2.28094663153693	8.57305698549771
H	3.32307696718670	4.30190833042119	11.77705905794537
H	3.57491692153627	2.32863380026816	10.28241532431612
C	6.42845364709385	7.72064444286656	10.00049990765469
C	5.15392232405725	8.09287693786843	9.52953559634567
C	7.25955428592991	8.66818059194233	10.62990102322587
C	4.72178608337740	9.40017436150511	9.69335448846078
C	6.80974479502010	9.97006199340669	10.78959356158937
C	5.54399498184861	10.34119415654123	10.32299803734528
H	4.53659359180814	7.36394820049283	9.00866747043685
H	8.22897976993002	8.36224701447809	11.01759146178608
H	3.74531554442105	9.69460480664409	9.31375535588649
H	7.44162355766627	10.69894440395778	11.29328748258848
H	5.19849090420146	11.36569981690017	10.44851988519842
C	9.80842455991901	3.08386976783394	2.02913077346023
H	10.04451601685376	4.12755487544881	1.79758458287547
H	9.57312617578121	2.54879580647756	1.10378821369899
H	8.95358889877665	3.03909205566793	2.71116693500312
C	13.34832291085050	4.12606801611190	9.50213635978701
H	12.78517986894736	3.19767113205094	9.63813429547925
H	14.35215357695593	3.89850706080269	9.12832117312273

H	13.42379470283788	4.66048454774788	10.45485213982067

References

[1] N. Allefeld, M. Grasse, N. Ignat'Ev, B. Hoge, Synthesis of unsymmetrically substituted phosphane oxides ($R^1R^2P(O)H$) and phosphinous acids (R^1R^2POH), *Chem. Eur. J.* 2014, 20, 8615–8620.

[2] X. Yang, Y. Zhao, X. Zhang, R. Li, J. Dang, Y. Li, G. Zhou, Z. Wu, D. Ma, W.-Y. Wong, X. Zhao, A. Ren, L. Wang, X. Hou, Thiazole-based metallophosphors of iridium with balanced carrier injection/transporting features and their two-colour WOLEDs fabricated by both vacuum deposition and solution processing–vacuum deposition hybrid strategy, *J. Mater. Chem.* 2012, 22, 7136.

[3] A. Belyaev, S. O. Slavova, I. V. Solovyev, V. V. Sizov, J. Jänis, E. V. Grachova, I. O. Koshevoy, Solvatochromic dual luminescence of Eu–Au dyads decorated with chromophore phosphines, *Inorg. Chem. Front.* 2019, 7, 140–149.

[4] A. Belyaev, B.-K. Su, Y.-H. Cheng, Z.-Y. Liu, N. M. Khan, A. J. Karttunen, P.-T. Chou, I. O. Koshevoy, Multiple emission of phosphonium fluorophores harnessed by the pathways of photoinduced counterion migration, *Angew. Chem. Int. Ed.* 2022, e202115690.

[5] G. M. Sheldrick, SHELXT – integrated space-group and crystal-structure determination, *Acta Crystallogr. A* 2015, 71, 3–8.

[6] G. M. Sheldrick, Crystal structure refinement with SHELXL, *Acta Crystallogr. C* 2015, 71, 3–8.

[7] O. V. Dolomanov, L. J. Bourhis, R. J. Gildea, J. A. K. Howard, H. Puschmann, OLEX2: A complete structure solution, refinement and analysis program, *J. Appl. Crystallogr.* 2009, 42, 339–341.

[8] F. Neese, Software update: The ORCA program system—version 5.0, *WIREs Comput. Mol. Sci.* 2022, 12, e1606.

[9] C. Adamo, V. Barone, Toward reliable density functional methods without adjustable parameters: The PBE0 model, *J. Chem. Phys.* 1999, 110, 6158–6170.

[10] F. Weigend, R. Ahlrichs, Balanced basis sets of split valence, triple zeta valence and quadruple zeta valence quality for H to Rn: Design and assessment of accuracy, *Phys. Chem. Chem. Phys.* 2005, 7, 3297–3305.

[11] S. Grimme, J. Antony, S. Ehrlich, H. Krieg, A consistent and accurate ab initio parametrization of density functional dispersion correction (DFT-D) for the 94 elements H–Pu, *J. Chem. Phys.* 2010, 132, 154104.

[12] S. Grimme, S. Ehrlich, L. Goerigk, Effect of the damping function in dispersion corrected density functional theory, *J. Comput. Chem.* 2011, 32, 1456–1465.

[13] F. Weigend, Accurate Coulomb-fitting basis sets for H to Rn, *Phys. Chem. Chem. Phys.* 2006, 8, 1057–1065.

[14] V. Barone, M. Cossi, Quantum calculation of molecular energies and energy gradients in solution by a conductor solvent model, *J. Phys. Chem. A* 1998, 102, 1995–2001.

[15] T. D. Goddard, C. C. Huang, E. C. Meng, E. F. Pettersen, G. S. Couch, J. H. Morris, T. E. Ferrin, UCSF ChimeraX: Meeting modern challenges in visualization and analysis, *Protein Sci.* 2018, 27, 14–25.
